# Subgenome phasing for complex allopolyploidy: case-based benchmarking and recommendations

**DOI:** 10.1093/bib/bbad513

**Published:** 2024-01-06

**Authors:** Ren-Gang Zhang, Hong-Yun Shang, Kai-Hua Jia, Yong-Peng Ma

**Affiliations:** State Key Laboratory of Plant Diversity and Specialty Crops/Yunnan Key Laboratory for Integrative Conservation of Plant Species with Extremely Small Populations, Kunming Institute of Botany, Chinese Academy of Sciences, Kunming 650201 Yunnan, China; University of Chinese Academy of Sciences, Beijing 101408 Beijing, China; State Key Laboratory of Plant Diversity and Specialty Crops/Yunnan Key Laboratory for Integrative Conservation of Plant Species with Extremely Small Populations, Kunming Institute of Botany, Chinese Academy of Sciences, Kunming 650201 Yunnan, China; Institute of Crop Germplasm Resources, Shandong Academy of Agricultural Sciences, Jinan 250100 Shandong, China; State Key Laboratory of Plant Diversity and Specialty Crops/Yunnan Key Laboratory for Integrative Conservation of Plant Species with Extremely Small Populations, Kunming Institute of Botany, Chinese Academy of Sciences, Kunming 650201 Yunnan, China

**Keywords:** subgenome phasing, WGDI, SubPhaser, complex allopolyploidy

## Abstract

Accurate subgenome phasing is crucial for understanding the origin, evolution and adaptive potential of polyploid genomes. SubPhaser and WGDI software are two common methodologies for subgenome phasing in allopolyploids, particularly in scenarios lacking known diploid progenitors. Triggered by a recent debate over the subgenomic origins of the cultivated octoploid strawberry, we examined four well-documented complex allopolyploidy cases as benchmarks, to evaluate and compare the accuracy of the two software. Our analysis demonstrates that the subgenomic structure phased by both software is in line with prior research, effectively tracing complex allopolyploid evolutionary trajectories despite the limitations of each software. Furthermore, using these validated methodologies, we revisited the controversial issue regarding the progenitors of the octoploid strawberry. The results of both methodologies reaffirm *Fragaria vesca* and *Fragaria iinumae* as progenitors of the octoploid strawberry. Finally, we propose recommendations for enhancing the accuracy of subgenome phasing in future studies, recognizing the potential of integrated tools for advanced complex allopolyploidy research and offering a new roadmap for robust subgenome-based phylogenetic analysis.

## INTRODUCTION

Polyploidy, where the genome of an organisms hosts multiple sets of chromosomes, is a pivotal driver in eukaryotic evolution [1, 2]. This process has been a catalyst for key evolutionary innovations, sparking diversification and speciation, especially in flowering plants [3–7]. Allopolyploidy, one of the primary forms of polyploidy, originates from hybridization events culminating in the consolidation of two or more distinct diploid species’ genomes within a single organism. Compared to autopolyploidy, which is triggered by chromosome duplication within a single species, allopolyploidy may amalgamate beneficial traits from the parent species, thus conferring a greater genetic diversity and adaptive potential [3]. Following polyploidy, the allopolyploid genome is able to undergo an evolutionary process of diploidization and genic fractionation, which involves chromosomal rearrangements and gene losses, potentially reverting to a diploid state in the end [8]. It has been noted that this process, as well as frequent homoeologous exchanges (HEs), could obscure the accurate detection of the origin and evolutionary footprints of these allopolyploid species, particularly following complex allopolyploidization [9].

Subgenome-aware phylogeny, a phylogenetic methodology based on a comprehensive gene set derived from the subgenomes of a polyploid, can offer a more robust and insightful framework than phylogenetic approaches at the gene or syntenic block level for deciphering the origin and evolutionary trajectories of polyploids. This subgenome-aware phylogenetic approach has been used to probe the evolutionary history, including diversification and polyploidization processes, not only in early angiosperms [10, 11] but also in recently formed allopolyploids and interspecific hybrids, including those found in cereals [12], trees [13], fruits [14], vegetables [15], herbs [16] and fish [17]. A vital step in these studies is the phasing of a polyploid’s subgenomes, which involves sorting the subgenomes according to their parental origin with the highest possible precision.

The subgenome phasing can be divided into two distinct categories: absolute phasing and relative phasing. To illustrate, we assume an allotetraploid (AABB) originating from two closely related diploid species, designated as AA and BB, each comprising two chromosomes: A1, A2 and B1, B2, respectively. Upon assembling these four chromosomes of the allotetraploid, the homoeologous pairings between A1 and B1 and between A2 and B2, are discerned based on synteny or genome alignments. Provided we have robust evidence, typically genomic data from the two diploid progenitors AA and BB, we can confidently segregate these into two sets: A1 + A2 and B1 + B2, a process referred to as ‘absolute phasing’. However, while this state is ideal, it is often unattainable. In some instances, such as the absence of diploid progenitors and other credible evidence, we arbitrarily segregate the chromosomes into either the A1 + A2 and B1 + B2 sets or the A1 + B2 and B1 + A2 sets. This is referred to as ‘relative phasing’. Despite this ambiguity in progenitor origins, the subgenome phylogeny can still reflect the true sister relationship between the two subgenomes A and B. However, relative phasing is not reasonable for subgenome phylogeny from complex allopolyploidization scenarios that involved more than two diploid progenitors or at least one intermediate allopolyploid progenitor. This is because the arbitrary phasing shuffles the relationships of the multiple subgenomes and would therefore lead to an inaccurate phylogeny.

Although the implementation of absolute phasing for an allopolyploid is feasible when extant diploid progenitors are present [9], many allopolyploids have no known progenitors, such as the allotetraploid frog *Xenopus laevis* [18] or the sage *Salvia splendens* [19]. In scenarios where extant or sampled progenitors are unknown or absent, SubPhaser has demonstrated its robustness and accuracy in phasing the subgenomes in dozens of neoallopolyploids (including tetra-, hexa- and octoploids) and homoploid interspecific hybrids [20]. It accomplishes this by utilizing repetitive sequences, primarily transposable elements (TEs), which have burst activity across the whole genome during the independent evolutionary periods of the progenitors, as ‘differential signatures’. Moreover, it can identify exchanges between subgenomes, including HEs and other inter-subgenomic translocations. A similar strategy has also been documented in a recent study [21]. This approach is able to produce absolute phasing by applying progenitor-specific evidence from before the hybridization event of an allopolyploid. However, this TE-based approach has its own limitations. Because it relies on the detection of progenitor-specific TE relics, (i) when these relics have been eliminated, as in paleoallopolyploids whose hybridization events occurred a long time ago, the subgenomes are not phased [21] and (ii) if the TEs are distributed unevenly across a chromosome/subgenome, the regions with sparse TEs are difficult to confidently identify as sites of potential inter-subgenomic exchanges [20].

WGDI represents another recent toolkit developed for the analysis of genomic polyploidization [22]. WGDI can be used to phase subgenomes for both neo- and paleo-allopolyploids based on the similarity and synteny of inter- and intra-genomes, as well as phylogeny [10, 11]. However, we were concerned that this evidence (similarity, synteny and phylogeny) would not guarantee absolute phasing when diploid progenitors were lacking, and that the resulting subgenome phylogeny might be misleading under a complex allopolyploidization scenario. Up to now, the absence of benchmark testing for subgenome phasing in complex polyploidization scenarios precludes the evaluation of methodological consistency. A recent study utilizing the WGDI subgenome system for phylogenetic analysis [23] caused us more concern. In the study, *Fragaria iinumae* was surprisingly dismissed as one of the progenitors of the allooctoploid cultivated strawberry, as the authors discovered that none of the four subgenomes of *F.* × *ananassa* was sister to *F. iinumae* on the subgenome phylogeny generated by WGDI [23]. However, this contradicts the consensus from multiple previous studies, in which *F. vesca* and *F. iinumae* are the two progenitors of the allooctoploid strawberry. The established consensus was based on genome-wide data and diverse non-subgenome phylogeny-based methodologies, including transposon [21], phylogeny [24, 25], distance [26] and alignment-based [27] approaches.

In this study, we evaluate and compare the effectiveness of WGDI and SubPhaser in resolving subgenome structures using four complex allopolyploidization cases, each with well-documented inter-subgenome evolutionary relationships, as benchmarks. Following affirmation of the methodologies through these four illustrative instances, we then revisited the subgenome phylogeny of the allooctoploid cultivated strawberry (*F.* × *ananassa*). We reaffirm that two of the four octoploid strawberry subgenomes are derived from diploid *F. vesca* and *F. iinumae* with subgenome phylogenies from both WGDI and SubPhaser. We evaluated WGDI as being an effective tool with regard to genome phasing, although it is not without its limitations. Drawing upon our experiences in these five cases, we provide recommendations for refining the use of WGDI for subgenome phasing in cases involving complex allopolyploidy (summarized in Box 1). These suggestions aim to address the current limitations of WGDI and bolster its accuracy and efficiency. Our study thus not only enhances the methods used to understand of the complex genomic landscapes of allopolyploid organisms but also provides practicable insights for future research.

**Box 1. Guidelines for phasing subgenomes in an allopolyploid complex: using wheats as an example**

For detailed files and pipeline codes used in the following guidelines, access at https://github.com/zhangrengang/subgenome_phasing_example/.

1.1 Subgenomes phasing with WGDI

1.1.1 Data preparation

Gather genomic data, including protein sequences in fasta format and gene coordinates in a custom gff format (see https://github.com/SunPengChuan/wgdi).

- Prepare genomic data for the allopolyploid complex. In this example, we utilize genomic data from *T. aestivum* (AABBDD) and *T. turgidum* (AABB).
- It is recommended to include genomic data of potential diploid progenitors, although we have omitted them for comparison purposes.
- Essential to have genomic data from an outgroup or ancestral karyotype. In our case, we employ *H. vulgare* as the outgroup reference.

1.1.2 Performing BLAST search

We employ DIAMOND for aligning protein sequences:

diamond blastp -q ^*^.pep -d ^*^.pep -o ^*^.blast …

1.1.3 Synteny detection and *Ks* calculation

To detect synteny and compute *Ks*, we utilize the ‘-icl’ option in WGDI for synteny identification, ‘-ks’ for *Ks* calculation, and ‘-bi’ to integrate the obtained information:

wgdi -icl ^*^.conf

wgdi -ks ^*^.conf

wgdi -bi ^*^.conf

1.1.4 [Optional] Exploring *Ks*-colored dot plots for similarity/orthology-based evidence

We can visualize *Ks*-colored dot plots with the ‘-bk’ option to identify similarity/orthology evidence that may help distinguish between subgenomes:

wgdi -bk ^*^.conf

From the dot plots (Figure S1A), it becomes evident that the A + B subgenomes of *T. aestivum* exhibit a lower *Ks* value when compared to the *T. turgidum* genome. Conversely, the D subgenome of *T. aestivum* stands as a singleton. Therefore, it is advisable to prioritize the initial phasing of the D subgenome. But there is no evidence to distinguish the A and B subgenomes by comparing their *Ks* distance to the D subgenome (Figure S1B).

1.1.5 Preliminary subgenome assignment

We obtain orthologous synteny using the ‘-c’ option, and then visually verify the orthology through *Ks*-colored dot plots employing the ‘-bk’ option:

wgdi -c ^*^.conf

wgdi -bk ^*^.conf

In cases when there are out-paralogous syntenic blocks that exhibit higher *Ks* values than orthologous syntenic blocks in the dot plots, it is necessary to fine-tune the parameters for the ‘-c’ option. Occasionally, manual removal of out-paralogous blocks from the output file might be required.

We obtain the karyotypes by mapping to the chromosomes of the outgroup reference using the ‘-km’ option:

wgdi -km ^*^.conf

**Figure 1:**
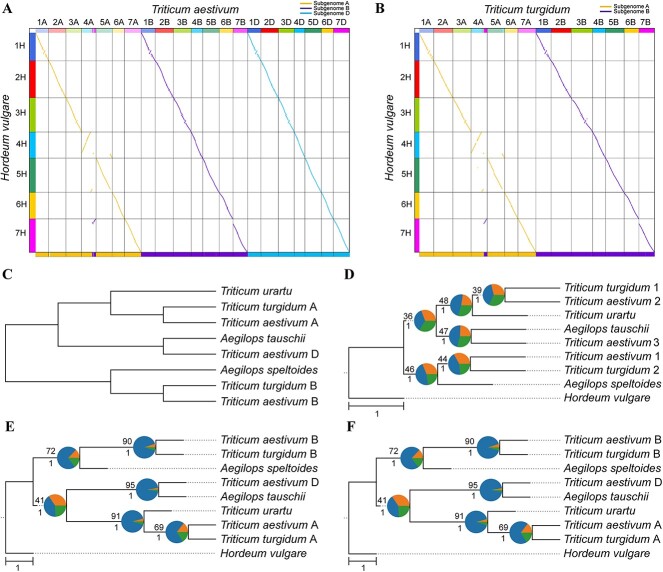
Subgenome phasing of the wheat genomes. (**A**, **B**) Comparison of subgenome assignments in *T. aestivum* (**A**) and *T. turgidum* (**B**) genomes between WGDI and SubPhaser. The above colored dot plots are from WGDI (further details in Figures S1 and Figure S2), and the colored bars at the bottom are from SubPhaser (further details in Figures S3 and Figure S4). (**C**) Subgenome phylogenetic topology as predicted from the literature. (**D**) Subgenome/species phylogeny from random sorting. The randomly sorted subgenomes were randomly labeled using numbers 1–3. (**E**) Subgenome/species phylogeny based on assignments of WGDI. (**F**) Subgenome/species phylogeny based on partition of SubPhaser. In (**D**–**F**), *H. vulgare* served as the outgroup. Numbers above the branches represent the percentages of concordance between gene and species/subgenome trees, and numbers below the branches represent the local posterior probabilities calculated in ASTRAL. Pie plots at the nodes represent the percentages of three gene tree topologies (q1, q2 and q3) calculated in ASTRAL. Bar, 1.0 coalescent unit.

We acquire karyotype files containing unassigned subgenomes and proceed with the following assignments. Subgenome D is assigned to number 3 based on *Ks* evidence, while subgenomes A/B are randomly designated as 1 or 2 due to a lack of substantial evidence. For fragmented segments characterized by broken synteny, assignments are made based on complementarity in synteny. As an illustrative example, we assign the large-scale translocation at the 3′-end of chr4A together with chr7B, as they exhibit complementary patterns, as depicted in Figure 1A.

We implement the assignments using the ‘-pc’ option and proceed to generate synteny alignments with the ‘-a’ option:

wgdi -pc ^*^.conf

wgdi -a ^*^.conf

1.1.6 Refining subgenome assignments through chromosome phylogeny (phylogeny-based evidence)

In this step, we construct phylogenetic trees for each chromosome, which provides a more robust framework than single gene-based phylogenies. This approach helps us gather additional robust evidence for distinguishing between subgenomes.

wgdi -at ^*^.chr^*^.conf

astral-pro -i ^*^.trees.nwk -o ^*^.trees.nwk.astral …

We proceed to manually adjust the assignments based on the consistent phylogenetic positions (Figure S2), In this refinement, we assign subgenome A (number 1) as the sister of D, and subgenome B (number 2) as the sister of the A + D clade for each chromosome. It’s worth noting that due to the robustness of the phylogeny-based evidence, we highly recommend considering this line of evidence for subgenome phasing, even if the absolute subgenome assignments were conducted solely based on the above *Ks* evidence.

1.1.7 [Optional] Exploring evidence through biased fractionation patterns (biased fractionation-based evidence)

We visualize gene fractionation patterns using the ‘-r’ option:

wgdi -r ^*^.conf

In our current study case, we have not identified biased fractionation patterns capable of distinguishing between subgenomes (Figure S28). However, when such evidence is available, it can serve as secondary evidence for subgenome assignments in future studies.

1.1.8 [Optional] Subgenome phylogeny reconstruction

As an optional final step, we proceed to reconstruct the subgenome phylogeny using ASTRL-Pro:

astral-pro -i ^*^.trees.nwk -o ^*^.trees.nwk.astral …

We strongly recommend users provide the detailed evidence outlined above as the basis for their subgenome assignments. This practice not only facilitates the evaluation of the results but also enhances the reproducibility of the study.

1.2 Phasing subgenomes with SubPhaser

1.2.1 Genomic data preparation

- To begin with, prepare the genomic assembly data, which should be in fasta format. In this example, we utilize genomic assemblies from *T. aestivum* (AABBDD) and *T. turgidum* (AABB).
- Additionally, the information of homoeologous relationships among chromosomes is required. This information can be obtained from the synteny analyses or whole-genome alignments conducted previously.

1.2.2 Executing SubPhaser

subphaser -i ^*^genome.fasta.gz -c ^*^sg.configure

Subsequently, it is crucial to verify the accuracy of subgenome phasing and assess the confidence of the identified potential inter-subgenomic exchanges. Here are the steps for validation: (i) The clustering heatmap and PCA plot (e.g. Figure S3B and Figure S3C) should be examined to determine if the subgenomes are well phased. Look for clear and distinguishable patterns of differential *k*-mers and homoeologous chromosomes. These patterns indicate that each subgenome possesses unique subgenome-specific features. (ii) Analyze the circos plot (e.g. Figure S3D) to identify windows where the enrichments (2nd circle from outer to inner circles) do not match the subgenome assignments of the chromosomes (1st circle). These discrepancies are identified as potential inter-subgenomic exchanges by SubPhaser. However, further manual verification is required to confirm these as true exchanges. For instance, in the case of the 3′ end of chr4A, look for significant enrichments of subgenome B-specific *k*-mers that are continuous (2nd circle). Compare the abundance of these *k*-mers with those found on the chromosomes of subgenome B (5th circle), which is contrasted with other subgenomes (4th and 6th circles). The evidence strongly supports the presence of exchanges, and we can confidently conclude that there has been an exchange at the 3′ end of chr4A, assuming no assembly errors. It is important to note that subgenome-specific *k*-mer distributions may not be evenly distributed across the genome. In cases where distributions are uneven (e.g., *Brassica* allopolyploids in Figures S19–S21), exercise caution when drawing inferences to avoid erroneous conclusions.

1.2.3 [Optional] Converting to WGDI format and subgenome phylogeny reconstruction

For comparative purpose, the phasing results from SubPhaser can be converted to the WGDI format using a custom script. Following this conversion, the subgenome phylogeny can be reconstructed using the same pipeline as above, if desired:

wgdi -pc ^*^.conf

wgdi -a ^*^.conf

wgdi -at ^*^.conf

astral-pro -i ^*^.trees.nwk -o ^*^.trees.nwk.astral …

Please note that the detailed codes and parameter files for the described pipeline can be accessed at https://github.com/zhangrengang/subgenome_phasing_example/. This repository will be continuously updated to provide the latest information and resources for subgenome phasing.

## RESULTS

### Overview

We undertook a comprehensive study to evaluate and compare the WGDI and SubPhaser methodologies when applied to the phasing of complex allopolyploid subgenomes. This study encompasses five instances, all of which are uniquely characterized by their complex allopolyploidization landscapes: four established ones and the controversial case of the cultivated strawberry. The subjects included allotetraploid emmer wheat (*Triticum turgidum*, AABB, 2*n* = 4*x* = 28) and allohexaploid bread wheat (*T. aestivum*, AABBDD, 2*n* = 6*x* = 42) [28], allotetraploid oat (*Avena insularis*, CCDD, 2*n* = 4*x* = 28) and allohexaploid common oat (*A. sativa*, AACCDD, 2*n* = 6*x* = 42) [12], neoallotetraploid opium poppy (*Papaver somniferum*, AACC, 2*n* = 4*x* = 22) and neoallooctoploid Troy poppy (*P. setigerum*, AABBCCDD, 2*n* = 8*x* = 44) [16], along with the three allotetraploids in the U’s triangle (allotetraploid brown mustard, *Brassica juncea*, AABB, 2*n* = 4*x* = 36; allotetraploid rapeseed, *B. napus*, AACC, 2*n* = 4*x* = 38; and allotetraploid Ethiopian mustard, *B. carinata*, BBCC, 2*n* = 4*x* = 34) [15] and the allooctoploid strawberry (*Fragaria × ananassa*, 2*n* = 8*x* = 56).

**Figure 2:**
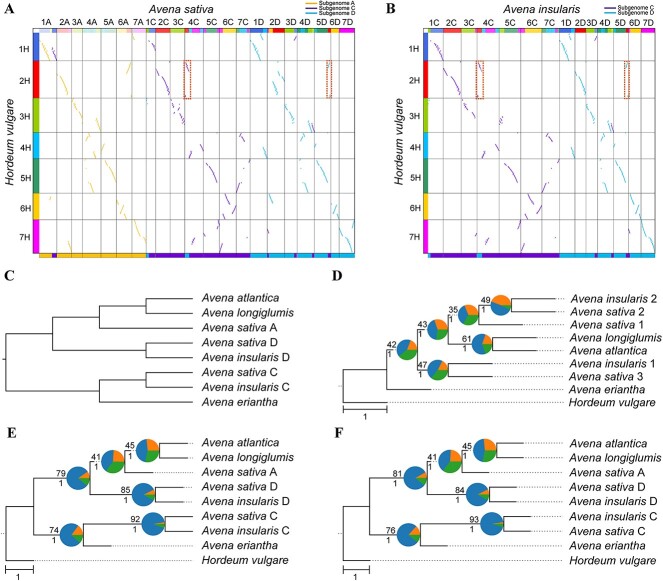
Subgenome phasing of the oat genomes. (**A**, **B**) Comparison of subgenome assignments in *A. sativa* (**A**) and *A. insularis* (**B**) genomes between WGDI and SubPhaser. The above colored dot plots are from WGDI (further details in Figures S6 and S7), and the bottom colored bars are from SubPhaser (further details in Figures S8–S11). Discrepancies between the two methods are highlighted with dashed squares. (**C**) Subgenome phylogenetic topology as predicted from the literature. (**D**) Subgenome/species phylogeny from random sorting. The randomly sorted subgenomes were randomly labeled using numbers 1–3. (**E**) Subgenome/species phylogeny based on assignments of WGDI. (**F**) Subgenome/species phylogeny based on partition of SubPhaser. In (**D**–**F**), *H. vulgare* served as the outgroup. Numbers above the branches represent the percentages of concordance between gene and species/subgenome trees, and numbers below the branches represent the local posterior probabilities calculated in ASTRAL. Pie plots at the nodes represent the percentages of three gene tree topologies (q1, q2 and q3) calculated in ASTRAL. Bar, 1.0 coalescent unit.

**Figure 3:**
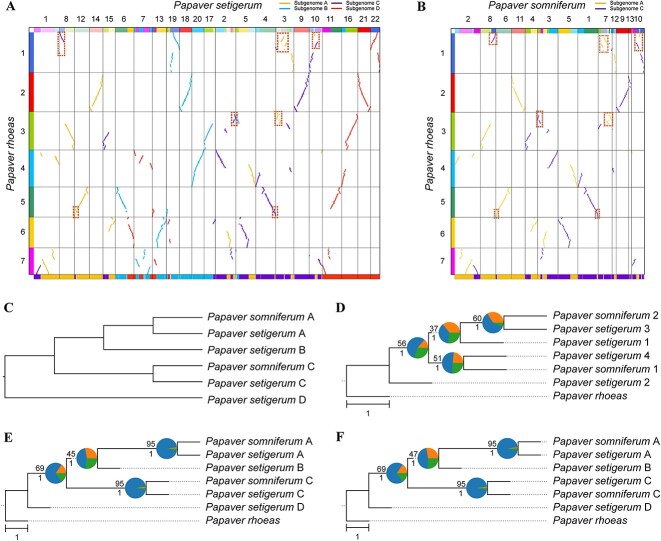
Subgenome phasing of the poppy genomes. (**A**, **B**) Comparison of subgenome assignments in *P. setigerum* (**A**) and *P. somniferum* (**B**) genomes between WGDI and SubPhaser. The above colored dot plots are from WGDI (further details in Figures S13 and S14), and the bottom colored bars are from SubPhaser (further details in Figures S15 and S16). Discrepancies between the two methods are highlighted with dashed squares. (**C**) Subgenome phylogenetic topology as predicted from the literature. (**D**) Subgenome/species phylogeny from random sorting. The randomly sorted subgenomes were randomly labeled using numbers 1–4. (**E**) Subgenome/species phylogeny based on assignments of WGDI. (**F**) Subgenome/species phylogeny based on partition of SubPhaser. In (**D**–**F**), *P. rhoeas* served as the outgroup. Numbers above the branches represent the percentages of concordance between gene and species/subgenome trees, and numbers below the branches represent the local posterior probabilities calculated in ASTRAL. Pie plots at the nodes represent the percentages of three gene tree topology (q1, q2 and q3) calculated in ASTRAL. Bar, 1.0 coalescent unit.

**Figure 4:**
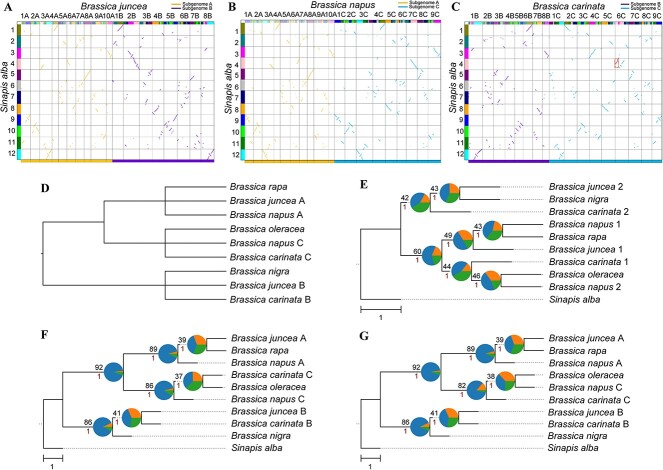
Subgenome phasing of the tetraploid *Brassica* genomes. (**A**–**C**) Comparison of subgenome assignments in *B. juncea* (**A**), *B. napus* (**B**) and *B. carinata* (C) genomes between WGDI and SubPhaser. The above colored dot plots are from WGDI (further evidence in Figures S17 and S18), and the bottom colored bars are from SubPhaser (further details in Figures S19–S21). Discrepancies between the two methods are highlighted with dashed squares. (**D**) Subgenome phylogenetic topology as predicted from the literature. (**E**) Subgenome/species phylogeny from random sorting. The randomly sorted subgenomes were randomly labeled using numbers 1 and 2. (**F**) Subgenome/species phylogeny based on assignments of WGDI. (**G**) Subgenome/species phylogeny based on assignments of SubPhaser. In (**E**–**G**), *S. alba* served as the outgroup. Numbers above the branches represent the percentages of concordance between gene and species/subgenome trees, and numbers below the branches represent the local posterior probabilities calculated in ASTRAL. Pie plots at the nodes represent the percentages of three gene tree topologies (q1, q2 and q3) calculated in ASTRAL. Bar, 1.0 coalescent unit.

**Figure 5:**
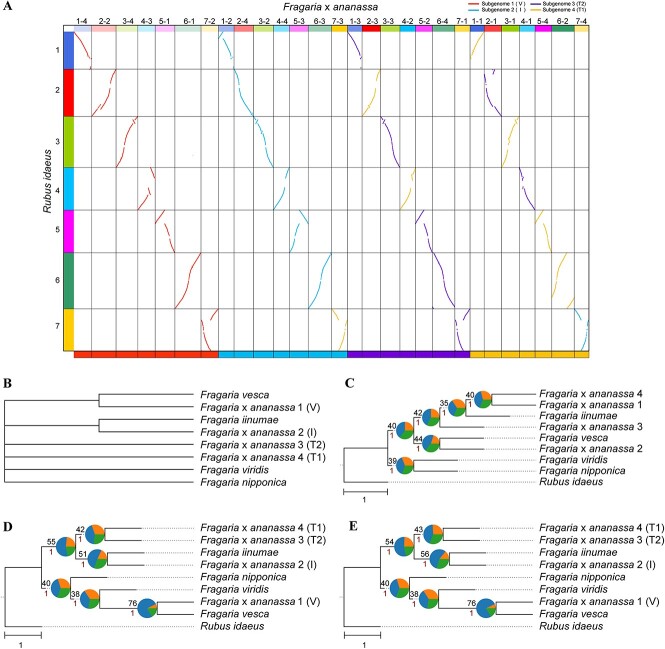
Subgenome phasing of the allooctoploid strawberry genome. (**A**) Comparison of subgenome assignments in *F.* × *ananassa* genomes between WGDI and SubPhaser. The above colored dot plots are from WGDI (Figures S23 and S24), and the bottom colored bars are from SubPhaser (further details in Figure S26). (**B**) Subgenome phylogenetic topology as common consensus from the literature. (**C**) Subgenome/species phylogeny from random sorting. The randomly sorted subgenomes were randomly labeled using numbers 1–4. (**D**) Subgenome/species phylogeny based on assignments of WGDI. (**E**) Subgenome/species phylogeny based on assignments of SubPhaser. In (**C**–**E**), *R. idaeus* served as the outgroup. Numbers above the branches represent the percentages of concordance between gene and species/subgenome trees, and numbers below the branches represent the local posterior probabilities calculated in ASTRAL. Pie plots at the nodes represent the percentages of three gene tree topologies (q1, q2 and q3) calculated in ASTRAL. Bar, 1.0 coalescent unit.

Our study was based on the premise that the diploid progenitors of these study organisms were either extinct or not sampled during the subgenome phasing process. This process was performed on a case-by-case basis using WGDI and was corroborated with synteny, similarity and phylogeny-based evidence. We also incorporated the absolute phasing results from SubPhaser as a control measure. Using these phasing results and genomic data from potential diploid progenitors, we reconstructed the subgenome/species phylogeny in each case to assess its accuracy in representing the genuine phylogeny. The anticipated phylogeny derived from existing literature served as a positive control, while the results from random subgenome sorting were used as a negative control (Figures 1–5). We also evaluate the accuracy by directly quantifying the percentage of optimal matches (best hits) between the phased subgenomes and the corresponding diploid progenitors.

### The wheat complex (tetraploid–hexaploid reticulate allopolyploidization)

Emmer wheat, an allotetraploid species (AABB, *T. turgidum*, 2*n* = 4*x* = 28), arose from the hybridization of two distinct ancestral species. It has two sets of chromosomes, with each set composed of two subgenomes (A and B) [28]. Bread wheat, a more prevalent and important wheat species globally, is an allohexaploid (AABBDD, *T. aestivum*, 2*n* = 6*x* = 42) and consists of three homoeologous subgenomes (A, B and D). Less than 0.8 million years ago (mya), a hybridization event between A (*T. urartu*) and B (a close relative of *Aegilops speltoides*) genomes gave rise to the allopolyploid emmer wheat (AABB). Subsequently, less than 0.4 mya, emmer wheat (AABB) hybridized with another wild wheat species carrying the D genome (*Ae. tauschii*), resulting in the allohexaploid bread wheat (AABBDD) [28].

We generated dot plots colored by synonymous substitution rate (*Ks*) distance using WGDI. We found that the allotetraploid emmer wheat shares a closer relationship (indicated by a notably lower *Ks*) with two of the three subgenomes of allohexaploid bread wheat, specifically the A and B subgenomes (Figure S1A). As a consequence, the D subgenome was the first to be separated out in the allohexaploid wheat. We then asked which of the remaining subgenomes (A or B) was genetically closer to the D subgenome, which could provide a key distinction between A and B. Unfortunately, the *Ks*-colored dot plots did not reveal any pattern (Figure S1B). This is likely to be due to the subtle differences in *Ks* distances that are difficult to discern visually. Therefore, we turned to a phylogeny-based method implemented in WGDI to investigate this further. Using the barley (*Hordeum vulgare*, 2*n* = 2*x* = 14) genome as an outgroup reference, we constructed phylogenetic trees for each chromosome using the ‘-pc’, ‘-a’ and ‘-at’ options in WGDI, in conjunction with the ASTRAL [29] tool. All seven generated trees showed identical phylogenetic topology (i.e. [[A, D], B]), with the D subgenome having the closest relationship to the A subgenome (Figure S2). As a consequence, both the A and B subgenomes were assigned based on the phylogeny of each chromosome (Figure 1A and B), assigning subgenome A as the sister of D and B as the sister of the A + D clade for each chromosome. Finally, using the phased subgenomes, we reconstructed the species/subgenome tree with the maximum number of genes. This tree was consistent with our expectations from the literature review [28] and also with the results from SubPhaser (Figure 1C–F). Indeed, the findings from WGDI and SubPhaser were nearly identical (Figure 1, Figure S5).

We therefore demonstrate using this case that absolute phasing is accessible for an allotetraploid–allohexaploid complex with similarity, synteny and phylogeny-based evidence implemented in WGDI. However, in other allotetraploid–allohexaploid cases, for example, assuming a phylogeny [D, [B, A]], where A and B are sisters, absolute phasing is not accessible, as A and B cannot be distinguished with WGDI because of their equivalent phylogenetic positions.

### The oat complex (tetraploid–hexaploid reticulate allopolyploidization)

The genomic structures and interrelationships of the tetraploid oat (*A. insularis*, CCDD) and the hexaploid common oat (*A. sativa*, AACCDD) have gained significant attention [12, 30]. The D-genome diploid progenitor is thought to be more closely related to the A-genome than to the C-genome and may be extinct. Cultivated ACD-genome hexaploid common oat (*A. sativa*) is believed to have originated around 0.5 mya from the hybridization between an A-genome diploid ancestor and a CD-genome tetraploid closely related to *A. insularis*, which originated from an allotetraploidy event between a C-genome and a D-genome diploid [12]. Frequent large-scale inter-subgenomic translocations including HEs have occurred among oat subgenomes [30].

In a manner analogous to the wheat scenario, the allotetraploid oat (CCDD, *A. insularis*) exhibits a closer genetic relationship to the C + D subgenomes than to the A subgenome of the allohexaploid common oat (AACCDD, *A. sativa*) as evidenced by the *Ks*-colored dot plots (Figure S6A). But there is an exception, with a large-scale translocation between chr1C and chr1A of the allohexaploid oat (Figure S6A). The evidence derived from the similarity-based analysis permitted the initial phasing of the A subgenome. Subsequently, the A subgenome demonstrated a notably lower *Ks* with the D subgenome (Figure S6B), leading to the assignment of the D subgenome as the close relative of the A subgenome. Taken together, these observations resulted in the phasing of all the subgenomes (Figure 2A and B), as further corroborated by the phylogeny ([[A, D], C]) of each chromosome (Figure S7).

A considerable divergence is apparent between the WGDI and SubPhaser methods, as underscored in Figure 2A and B. This discrepancy is attributed to the balanced (or reciprocal) HEs between subgenomes C and D (the anterior segment of chr4C and the posterior segment of chr5D in both oats), which have been demonstrated by Kamal *et al*. [30], as these exchanges evade detection using synteny-based methods and are omitted from chromosome phylogenies calculated in WGDI. However, the species/subgenome tree aligns with our expectations and is similar to the tree derived from SubPhaser, notwithstanding a marginally lower percentage of concordance between gene trees and the species/subgenome tree (Figure 2C–F) and lower percentage of best hits to corresponding diploid progenitors (Figure S12).

Overall, the above two cases reveal a generic model. When an allotetraploid–allohexaploid complex, denoted as AABB and AABBCC, has an inherent evolutionary scenario in which lineage C is sister to either A or B, absolute phasing is possible. In contrast, if C is sister to A + B clade, absolute phasing may be impossible for A and B, at least when only using WGDI. Under the circumstances, theoretically at least, one diploid progenitor of A or B is essential for absolute phasing with WGDI. Nevertheless, relative phasing of A and B has no effect on their sister relationship.

### The poppy complex (tetraploid–octoploid reticulate allopolyploidization)

The species complex comprising *P. somniferum* (opium poppy, a neoallotetraploid with 2*n* = 4*x* = 22, AACC) and *P. setigerum* (Troy poppy, a neoallooctoploid with 2*n* = 8*x* = 44, AABBCCDD) exemplifies the phenomenon of reticulate allopolyploidization, which can contribute to a complex network of evolutionary relationships [16]. With multiple lines of evidence, *P. setigerum* has been demonstrated to have originated around 0.4 mya as a result of hybridization events involving *P. somniferum* as one of its allotetraploid progenitors and another potentially extinct allotetraploid progenitor (BBDD) [16]. Prior to the allooctoploidy, both the allotetraploid progenitors were independently formed from distant, potentially extinct diploid progenitors (AA and CC, and BB and DD) around 0.5 and 0.9 mya, respectively. Frequent large-scale inter-subgenomic exchanges have also occurred among these subgenomes, especially between subgenomes A and C and between B and D [16].

We utilized *Ks*-colored dot plots to differentiate between the A + C and B + D subgenomes within the neoallooctoploid Troy poppy (AABBCCDD, *P. setigerum*), since these subgenomes exhibited *Ks* patterns distinct from those of the neoallotetraploid poppy (AACC, *P. somniferum*) (Figure S13A). However, similar to wheat, we were unable to achieve further distinctions using similarity-based methods (Figure S13B). We then used the *P. rhoeas* (2*n* = 2*x* = 14) genome as an outgroup reference for reconstructing the phylogenetic tree of each chromosome. All seven trees demonstrated consistent topology, specifically [D, [C, [B, A]]] (Figure S14). Thus homoeologous chromosomes of the same phylogenetic position were categorized into the same subgenome with WGDI. Despite some errors in the phasing results of WGDI when juxtaposed with those from SubPhaser (Figure 3A and B), which were attributable to balanced HEs (e.g. the HE between chr4 and chr12 of *P. setigerum*, as highlighted in Figure 3A) and random sorting of ambiguous syntenic blocks (e.g. the fragmented blocks in chr10 of *P. somniferum*, as highlighted in Figure 3B), the overall subgenome topology aligned both with our expectations and with the SubPhaser results (Figure 3C–F). The discrepancies in the percentage of concordance between gene and subgenome trees was small (Figure 3E and F).

This allotetraploid–allooctoploid case is more complicated than the allotetraploid–allohexaploid (wheats and oats) cases described above. Nevertheless, the unambiguous inherent evolutionary scenario ([D, [C, [B, A]]]) leads to the possibility of absolute phasing. However, assuming that the phylogeny is [[D, B], [C, A]]] which at least the two subgenomes in the tetraploid genome are sister groups, there may not be any efficient way to distinguish A from C, or B from D, in the allooctoploid genome, using WGDI. Thus, this uncertainty should be noted for other allotetraploid–allooctoploid complexes.

### Allotetraploids in the U’s triangle (tetraploid–tetraploid–tetraploid parallel allopolyploidization)

The U’s triangle elucidates the relationships among six species in the genus *Brassica*, comprising three diploid and three allotetraploid species [15]. The diploid species are *B. rapa* (AA, 2*n* = 2*x* = 20), *B. nigra* (BB, 2*n* = 2*x* = 16) and *B. oleracea* (CC, 2*n* = 2*x* = 18). Through natural hybridization and chromosome doubling, these species have independently given rise to three allotetraploid species: *B. juncea* (AABB, 2*n* = 4*x* = 36, brown mustard), *B. napus* (AACC, 2*n* = 4*x* = 38, rapeseed) and *B. carinata* (BBCC, 2*n* = 4*x* = 34, Ethiopian mustard) [15].

By applying *Ks*-colored dot plots to compare every two combinations among the three allotetraploids (Figure S17), all subgenomes were successfully phased with WGDI (Figure 4A–C). The phasing results were substantiated using the chromosome phylogeny (Figure S18). The results from WGDI differ slightly from those from SubPhaser (Figure 4A). Specifically, in *B. carinata*, a translocation from the B subgenome occurred in the gene-rich region of the C subgenome. In this region (5′ end of chromosome 6C), differential signatures, primarily TEs, are too sparse for SubPhaser to be able to identify the translocation confidently (Figure S21). Consequently, the percentages of concordance between gene trees and the subgenome/species tree from WGDI are marginally higher than those obtained from SubPhaser (Figure 4F and G), as is the percentage of best hits to corresponding progenitors (Figure S22), yet both methodologies yield the expected phylogeny (Figure 4D–G).

In this case, when two allotetraploids share a diploid progenitor, simply comparing the two tetraploid genomes led to absolute phasing with WGDI. There appears to be no uncertainty or limitation in such cases.

### The allooctoploid strawberry

Finally, we investigated the contentious subgenome structure of the allooctoploid cultivated strawberry (*Fragaria × ananassa*, 2*n* = 8*x* = 56). Common consensus, based on comparisons of the cultivated strawberry with extant diploid relatives, suggests that one of the four subgenomes (termed V) is closely related to the diploid woodland strawberry (*F. vesca*), while a second subgenome (termed I) is closely related to *F. iinumae* [21, 24–27]. The diploid progenitors for the remaining two subgenomes (termed T1 and T2 referring to [21]) are not known definitively. Through the utilization of the WGDI methodology, Zhou *et al*. [23] contradicted the widely held view that *F. iinumae* was one of the progenitors. In their conclusion, three subgenomes of *F. × ananassa* belong to the *F. vesca* group, and one is sister to *F. viridis* [23]. We reanalyzed the genomic data of allooctoploid strawberry to evaluate this extraordinary claim.

In this case, discernible patterns to distinguish subgenomes in the *Ks*-colored dot plots are absent (Figure S23). Consequently, we adopted the phylogeny-based approach by WGDI, which has been demonstrated to be effective in previous instances, using *Rubus idaeus* (2*n* = 14) as the outgroup. The seven chromosome sets revealed a consistent phylogenetic topology across all four subgenomes, specifically [subgenome V, [subgenome I, [subgenome T1, subgenome T2]]] mostly, with subgenome V occupying the basal position, followed by subgenome I and subgenomes T1 and T2 representing reciprocal sister groups (Figure S24). We assigned chromosome sets with the same phylogenetic position to the corresponding subgenome (Figure 5A). Subgenomes T1 and T2 were assigned arbitrarily because of their equivalent positions.

In this case, there were large differences between the subgenome assignments of WGDI and SubPhaser (Figure 5A), while the results from the latter were identical with those from previous studies in either the V and I subgenomes [24–27] or all the four subgenomes [21]. Firstly, the assignments of subgenomes T1 and T2 deviated from those provided by SubPhaser (chromosomes 2-3 and 4-2 versus 2-1 and 4-1; Figure 5A). This discrepancy is due to the substantially equivalent phylogenetic positions of subgenomes T1 and T2 (Figure S24), resulting in their assignments by WGDI being essentially random and not evidence-supported (i.e. ‘relative phasing’). Second, there is a reversal between chromosomes 7-4 and 7-3 (Figure 5A). This is because of the substantive discordance of chromosome phylogeny between chromosome 7 and other chromosomes. Similar discordance on chromosome 7 has also been observed in a previous study [25]. However, when we included the four diploid potential progenitors (*F. vesca*, *F. iinumae*, *F. viridis* and *F. nipponica*), the reversal could be fixed (Figure S25). This phylogenetic uncertainty can be attributed to widespread incomplete lineage sorting (ILS) and hybridization across strawberry genomes [27], as well as potential HEs between subgenomes [24]. This is in agreement with the quite low percentages (approximately 40%) of concordance between gene and chromosome trees (Figure S24).

We assessed subgenome phylogeny with potential diploid progenitors and discovered slight differences between the two methods (Figure 5B–E), notwithstanding the significant differences in subgenome assignments (Figure 5A). Subgenomes T1 and T2 are sister groups in the phylogeny, and therefore, the inconsistent phasing of chromosomes 2 and 4 has no significant impact on the phylogeny. The impact of the incorrect assignment of chromosome 7 (Figure 5D and E, Figure S27) was mostly counteracted by the large discordance between gene trees and species/subgenome trees.

Nevertheless, our results from both methods validate *F. vesca* and *F. iinumae* as the diploid progenitors (Figure 5D and E, Figure S25), in support of the common consensus [21, 24–27]. The other two subgenomes are sisters and belong to the *F. iinumae* group (Figure 5D and E, Figure S25), supporting some of the previous research [21, 25]. In total, our findings (even from the same WGDI software and the same strawberry genome set) differed largely from those of Zhou *et al*. [23], which could be attributed to the misuse/misinterpretation or poor reproducibility of WGDI due to the need for users to manually assign subgenomes.

## DISCUSSION

### Validation of subgenome phasing by WGDI, its advantages and limitations and future recommendations as to its use

WGDI is a comprehensive toolkit offering multiple lines of evidence, including similarity (*Ks*), synteny and phylogeny-based approaches, for the assignment of subgenomes. It also provides diverse options for integrating and visualizing these pieces of evidence. However, there are a few limitations to its functionality, according to our experience in the cases described above. Firstly, phylogeny by syntenic blocks has not yet been implemented to evaluate HEs or random sorting of some fragmented blocks. This results in some phasing errors (e.g. errors in the poppy case, Figure 3) not being detected with WGDI. Second, the extraction of orthologous synteny or removal of out-paralogous synteny relies heavily on parameter settings and sometimes requires manual removal of out-paralogous syntenic blocks. Thirdly, unlike SubPhaser, WGDI does not provide a definitive determination for assignment. Instead, it requires users to manually determine how to sort the subgenomes, which may weaken the reproducibility. Therefore, we recommend users to provide detailed evidence underlying their subgenome assignment to evaluate the results and to strengthen the reproducibility.

Nevertheless, WGDI remains a convenient and generally accurate toolkit, and in our study, most results obtained from WGDI were in line with those from SubPhaser. A combination of SubPhaser and WGDI is ideal, particularly for assigning subgenomes in neoallopolyploids with complicated evolutionary trajectories. SubPhaser offers subgenome-specific evidence based on TEs, while WGDI offers syntenic gene-based evidence. Thus, this combination is particularly suitable for genomes with uneven distributions of both genes and TEs, such as the *Brassica* allopolyploids.

Although we used multiple lines of evidence to assign subgenomes with WGDI, we found that the phylogeny-based approaches were the most robust. Phylogeny reflects the evolutionary history, leading to an identifiable phylogenetic position for each subgenome except for sisters (e.g. subgenomes T1 and T2 of the allooctoploid strawberry, Figure 5). However, the phylogeny can be complicated by a number of biological factors, such as ILS and introgression [31] within the progenitors or their relatives, as well as HEs that occur between subgenomes [9]. In this study, we use homoeologous chromosome phylogeny to assign subgenomes by WGDI, which is a more robust method than using gene phylogenies, because the use of multiple gene sets can reduce the effects of ILS [32]. On the other hand, minor HEs would not be highlighted by chromosome phylogeny, which leads to WGDI not recognizing HEs.

Another piece of evidence that can be useful in the sorting of retained duplicated genomic regions is the patterns of biased fractionation or subgenome dominance in allopolyploids [33, 34]. However, in the cases studied in this paper, we did not observe such differential patterns with WGDI (Figures S28–S32), perhaps due to the recent formation of these allopolyploids. Moreover, as biased fractionation occurs after the formation of an allopolyploid, there is theoretically no guarantee that homoeologous chromosomes from the same progenitor would share the same biased pattern. Nonetheless, when the evidence is available, it can be used as auxiliary evidence for assigning subgenomes.

With WGDI, translocations from one subgenome to another subgenome that have broken the synteny can be easily identified based the complementarity of syntenic blocks or chromosomal segments. For example, there is a large translocation at 3′-end of chr4A in wheat. This segment is complemented with chr7B to make up an intact chromosome, the chr7H of barley. So the segment has been correctly assigned together with chr7B. However, canonical HEs that do not break the synteny [35] cannot be recognized with complementarity using WGDI, e.g. the reciprocal HE between chr4D and chr5C in the oat genomes. This kind of HE can be identified using phylogenies of homoeologous genes or blocks [9], which have not yet been implemented in WGDI. Thus, an additional evaluation of phylogenies by block or sliding window would be preferable for the WGDI results.

Surprisingly, in most cases (i.e. in wheat, poppy and *Brassica*), we observed a true subgenome phylogenetic topology when randomly sorting subgenomes using WGDI (Figures 1–5). This may be attributed to the presence of unbalanced subgenome assignments, as can be observed in Figures S5 and S22. However, the randomly sorted subgenome phylogeny indicates much shorter branch lengths in coalescence units by ASTRAL, and the concordances between gene trees and species/subgenome trees are much lower than those based on absolute phasing (Figures 1–5). This suggests that the phylogeny is not stable and contains many internal errors. Moreover, the randomly sorted subgenome phylogeny or alignment is in theory not usable in downstream analyses, such as estimating split times. Thus, random sorting is not recommended in practice, unless relative phasing is considered appropriate for sister subgenomes (e.g. subgenomes T1 and T2 of allooctoploid strawberry, Figure 5) when no further evidence is available.

Furthermore, we summarized these above recommendations, as well as our best practices for phasing subgenomes using WGDI and SubPhaser, in Box 1. This box outlines the key guidelines for our methodologies, to serve as a straightforward, easily accessible roadmap for users. To provide practicable, hands-on guidance, we also included an example code repository on GitHub (https://github.com/zhangrengang/subgenome_phasing_example/). This repository offers a step-by-step example of our methods, thereby enhancing accessibility for researchers engaged in the study of allopolyploid genomes.

### Potential applications in complex paleoallopolyploidization scenarios

The presence of complex allopolyploidization scenarios in neoallopolyploids from multiple lineages suggests that similar complex evolutionary scenarios are likely to have occurred in paleoallopolyploidy. For instance, a hypothesis has been proposed that the paleotetraploidy of the order Ranunculales (early-diverging eudicots) contributed to the *γ* paleohexaploidy of the core eudicots via a two-step process [36, 37], although this view has been met with considerable oppositions [22, 38–42]. Due to the lack of well-documented complex paleoallopolyploidy cases as benchmarks, such a complex paleoallopolyploidization scenario was not included in our evaluation. However, with the fast development of genome sequencing, we anticipate that we will be attracted by many ancient and complex allopolyploidization cases. If the paleoallopolyploids retain a large degree of synteny after post-polyploidization diploidization, methods similar to those in this study could also be used to identify homoeologous relationships and to reconstruct the phylogenies of chromosomes or blocks, allowing the subgenome-aware evolutionary history of the complex paleoallopolyploidy to be retraced. Despite the fact that paleoallopolyploidy may involve a wide range of genome fractionation and chromosomal rearrangements, which make subgenome assignment more challenging, the combination of multiple lines of evidence (including synteny, phylogeny and similarity implemented in WGDI) could potentially provide valuable insight into the process of complex paleoallopolyploidy.

## CONCLUSION

Investigating the subgenomic structures and evolutionary histories of complex allopolyploids poses many challenges. However, this study underscores the efficacy of integrating multiple lines of method and evidence in the elucidation of these intricate dynamics. We suggest that while WGDI and SubPhaser are not without limitations, their combined application can provide valuable insights into the evolutionary trajectories of complex allopolyploidy. These findings offer practical considerations for upcoming research from the fast development of genomic sequencing techniques. The refinement and development of the subgenome phasing technique will enable us to move closer to fully deciphering the role of allopolyploidy in plant evolution and diversification.

## METHODS

### Data collection

The genomic data from *T. turgidum* [43], *T. aestivum* v2.1 [44], *T. urartu* [45], *Ae. speltoides* [46], *Ae. tauschii* [47], *Avena insularis*, *A. sativa* and *A. longiglumis* [30], *A. atlantica* and *A. eriantha* [48], *H. vulgare* MorexV3 [49] (the outgroup in the analysis of both wheats and oats), *P. rhoeas* (which served as the outgroup in the analysis of the following two poppy species), *P. somniferum* and *P. setigerum* [50], *B. juncea* [51], *B. napus* zs11 [52], *B. carinata* [53], *B. oleracea* [54], *B. rapa* [55], *B. nigra* and *Sinapis alba* [56] (the outgroup for the analysis of *Brassica* spp.), *F.* × *ananassa* FL15.89–25 v1.0 [57], *F. vesca* v6.0 [23], *F. iinumae* [26], *F. viridis* [27], *F. nipponica* [58] and *R. idaeus* [59] (the outgroup for the analysis of the strawberries), were obtained from public databases or were provided by the corresponding authors, as detailed in Table S1. Because the genome assembly of *F. nipponica* was too fragmented to detect enough syntenic blocks, we constructed its chromosome-scale scaffolds using RagTag [60] v1.1.1 with the *F. vesca* genome as the reference.

### Phasing subgenomes with WGDI

Protein sequences were aligned using DIAMOND [61] v0.9.24. Inter- and intra-genomic syntenic blocks were identified with the ‘-icl’ option of WGDI [22] v0.6.2. The synonymous substitution rate (*Ks*) was calculated with the ‘-ks’ option of WGDI, and synteny and *Ks* were integrated with the ‘-bi’ option of WGDI. Syntenic dot plots colored with *Ks* values were plotted with the ‘-bk’ option of WGDI, and syntenic blocks were filtered using the ‘-c’ option of WGDI to obtain orthologous synteny, sometimes with manual adjustments. The orthologous synteny was visually validated with the ‘-bk’ option of WGDI. Based on the inter- and intra-genomic synteny and *Ks* values, we further tried to phase the synteny blocks into subgenomes (details for each case in Results section). Then, we manually assigned the subgenomes by recording the subgenomic regions on the chromosomes of the reference (from the ‘-km’ option of WGDI) and used WGDI (-pc, -a) to obtain the hierarchical gene list (one subgenome per column), using the outgroup as a reference. The hierarchical gene lists were used to infer maximum likelihood (ML) trees in IQ-TREE [62] v2.2.0.3 with automatic selection of the best-fit substitution mode through WGDI (-at option). These gene trees were then used as input into ASTRAL-Pro [29] v1.10.1.3 to infer a species/subgenome/chromosome phylogeny. The phylogeny was visualized and evaluated using Newick utilities [63] and PhyTop (https://github.com/zhangrengang/phytop). The above process (-pc, -a and -at options of WGDI and ASTRAL-Pro) was iterated until each chromosome from the same subgenome was lying on the same phylogenetic position on the chromosome-level tree. Gene retention or fractionation of subgenomes was visualized using the ‘-r’ option of WGDI. In addition to the phylogeny-based evaluation, we also directly quantified the percentage of best DIAMOND hits between phased subgenomes and the multiple diploid progenitors.

After subgenome assignments using similarity and phylogeny-based evidence, we re-sorted the assignments randomly. In brief, for each allopolyploid genome, we randomly reshuffled the previous assignments within each homoeologous chromosome set according to the outgroup reference genome, producing extensive artificial HEs, and then the random assignments were input into WGDI (-pc, -a, -at options) and ASTRAL-Pro as described above to generate a species/subgenome tree.

### Phasing subgenomes with SubPhaser

SubPhaser [20] is an automated pipeline based on subgenome-specific *k-*mers. Genome assembly and homoeologous relationships of chromosomes were input into SubPhaser v1.2 and the resulting output was the phasing results. Potential exchanges (including inter-subgenomic translocations and HEs) between subgenomes were also identified using SubPhaser and further manually curated following previous studies [16, 20]. Subgenomes were split and grouped according to the confidently identified exchanges, and the partitioned subgenomes were then input into WGDI (-pc, -a, -at options) and ASTRAL-Pro as described above to generate a species/subgenome tree. For the tetraploid *Brassica* spp. and *F.* × *ananassa*, subgenome assignments without identified HEs from SubPhaser were used directly, since there were too few differential signatures to confidently identify HEs with SubPhaser in these genomes (Figures S19–S21 and S26). Additional modifications were made for phasing the hexaploid *A. sativa* genome. The three subgenomes were successfully phased in a default SubPhaser run, but the A and D subgenomes were too closely related to obtain as many specific *k*-mers as the C subgenome (Figure S8). Thus, based on the initial run, we re-ran the A + C and D + C subgenome combinations separately to obtain more subgenome-specific *k*-mers to refine the identified exchanges (Figures S9 and S10). The phasing results from *T. turgidum*, *T. aestivum*, *P. somniferum*, *P. setigerum* and *B. carinata* (Figures S3 and S4, S15 and S16 and S21) were adopted from those generated in previous studies [16, 20].

**Key Points**

- We evaluated and compared the ability to phase subgenomes using WGDI and SubPhaser based on well-documented complex allopolyploidy cases.
- Most results obtained from WGDI were in line with those from SubPhaser, consistent with prior research.
- The results of both methodologies reaffirm *Fragaria vesca* and *F. iinumae* as the progenitors of the octoploid strawberry.
- We propose recommendations for the accurate subgenome phasing by WGDI in future research.

## Supplementary Material

supplement-wgdiwork-11_18_bbad513

## Data Availability

The example code of our methodologies is available at https://github.com/zhangrengang/subgenome_phasing_example/.

## Notes

**Ren-Gang Zhang** is a PhD candidate at the State Key Laboratory of Plant Diversity and Specialty Crops / Yunnan Key Laboratory for Integrative Conservation of Plant Species with Extremely Small Populations, Kunming Institute of Botany, Chinese Academy of Sciences, and University of Chinese Academy of Sciences. His research interests are evolutionary genomics of polyploids, genetics and bioinformatics.

**Hong-Yun Shang** is a Research Assistant at the State Key Laboratory of Plant Diversity and Specialty Crops / Yunnan Key Laboratory for Integrative Conservation of Plant Species with Extremely Small Populations, Kunming Institute of Botany, Chinese Academy of Sciences. His research interests are genomics and population genetics.

**Kai-Hua Jia** is an Assistant Professor at the Institute of Crop Germplasm Resources, Shandong Academy of Agricultural Sciences. His research primarily focuses on the evolution of polyploid genomes, adaptive evolution, and the molecular mechanisms of stress resistance.

**Yong-Peng Ma** is a Professor at the State Key Laboratory of Plant Diversity and Specialty Crops / Yunnan Key Laboratory for Integrative Conservation of Plant Species with Extremely Small Populations, Kunming Institute of Botany, Chinese Academy of Sciences. His research interests are population and conservation genomics.

## References

[1] Comai L . The advantages and disadvantages of being polyploid. Nat Rev Genet 2005;6:836–46. 16304599 10.1038/nrg1711

[2] Otto SP . The evolutionary consequences of polyploidy. Cell 2007;131:452–62. 17981114 10.1016/j.cell.2007.10.022

[3] Van de Peer Y, Mizrachi E, Marchal K. The evolutionary significance of polyploidy. Nat Rev Genet 2017;18:411–24. 28502977 10.1038/nrg.2017.26

[4] Soltis PS, Soltis DE. Ancient WGD events as drivers of key innovations in angiosperms. Curr Opin Plant Biol 2016;30:159–65. 27064530 10.1016/j.pbi.2016.03.015

[5] Landis JB, Soltis DE, Li Z, et al. Impact of whole-genome duplication events on diversification rates in angiosperms. Am J Bot 2018;105:348–63. 29719043 10.1002/ajb2.1060

[6] Tank DC, Eastman JM, Pennell MW, et al. Nested radiations and the pulse of angiosperm diversification: increased diversification rates often follow whole genome duplications. New Phytol 2015;207:454–67. 26053261 10.1111/nph.13491

[7] Eric Schranz M, Mohammadin S, Edger PP. Ancient whole genome duplications, novelty and diversification: the WGD radiation lag-time model. Curr Opin Plant Biol 2012;15:147–53. 22480429 10.1016/j.pbi.2012.03.011

[8] Li Z, McKibben MTW, Finch GS, et al. Patterns and processes of diploidization in land plants. Annu Rev Plant Biol 2021;72:387–410. 33684297 10.1146/annurev-arplant-050718-100344

[9] Edger PP, McKain MR, Bird KA, VanBuren R. Subgenome assignment in allopolyploids: challenges and future directions. Curr Opin Plant Biol 2018;42:76–80. 29649616 10.1016/j.pbi.2018.03.006

[10] Ma J, Sun P, Wang D, et al. The *Chloranthus sessilifolius* genome provides insight into early diversification of angiosperms. Nat Commun 2021;12:6929. 34836967 10.1038/s41467-021-26931-3 PMC8626421

[11] Sun P, Yang Y, Liu L, et al. Early diversification and karyotype evolution of flowering plants. Res Sq 2022. 10.21203/rs.3.rs-1410884/v1.

[12] Peng Y, Yan H, Guo L, et al. Reference genome assemblies reveal the origin and evolution of allohexaploid oat. Nat Genet 2022;54:1248–58. 35851189 10.1038/s41588-022-01127-7 PMC9355876

[13] An X, Gao K, Chen Z, et al. High quality haplotype-resolved genome assemblies of *Populus tomentosa* Carr., a stabilized interspecific hybrid species that is widespread in Asia. Mol Ecol Resour 2021;22:786–802. 34549890 10.1111/1755-0998.13507

[14] Zhang T, Qiao Q, Du X, et al. Cultivated hawthorn (*Crataegus pinnatifida* var. *major*) genome sheds light on the evolution of Maleae (apple tribe). J Integr Plant Biol 2022;64:1487–501. 35748532 10.1111/jipb.13318

[15] Yim WC, Swain ML, Ma D, et al. The final piece of the triangle of U: evolution of the tetraploid *Brassica carinata* genome. Plant Cell 2022;34:4143–72. 35961044 10.1093/plcell/koac249 PMC9614464

[16] Zhang RG, Lu C, Li G, et al. Subgenome-aware analyses suggest a reticulate allopolyploidization origin in three *Papaver* genomes. Nat Commun 2023;14:2204. 37076529 10.1038/s41467-023-37939-2 PMC10115784

[17] Wang Y, Li X, Xu W, et al. Comparative genome anatomy reveals evolutionary insights into a unique amphitriploid fish. Nat Ecol Evol 2022;6:1354–66. 35817827 10.1038/s41559-022-01813-z PMC9439954

[18] Session AM, Uno Y, Kwon T, et al. Genome evolution in the allotetraploid frog *Xenopus laevis*. Nature 2016;538:336–43. 27762356 10.1038/nature19840 PMC5313049

[19] Jia KH, Liu H, Zhang R, et al. Chromosome-scale assembly and evolution of the tetraploid *Salvia splendens* (Lamiaceae) genome. Hortic Res 2021;8:177. 34465761 10.1038/s41438-021-00614-y PMC8408255

[20] Jia KH, Wang Z, Wang L, et al. SubPhaser: a robust allopolyploid subgenome phasing method based on subgenome-specific *k*-mers. New Phytol 2022;235:801–9. 35460274 10.1111/nph.18173

[21] Session AM, Rokhsar DS. Transposon signatures of allopolyploid genome evolution. Nat Commun 2023;14:3180. 37263993 10.1038/s41467-023-38560-z PMC10235133

[22] Sun P, Jiao B, Yang Y, et al. WGDI: a user-friendly toolkit for evolutionary analyses of whole-genome duplications and ancestral karyotypes. Mol Plant 2022;15:1841–51. 36307977 10.1016/j.molp.2022.10.018

[23] Zhou Y, Xiong J, Shu Z, et al. The telomere-to-telomere genome of *Fragaria vesca* reveals the genomic evolution of *Fragaria* and the origin of cultivated octoploid strawberry. Hortic Res 2023;10:uhad27. 10.1093/hr/uhad027 PMC10116950 37090094

[24] Edger PP, Poorten TJ, VanBuren R, et al. Origin and evolution of the octoploid strawberry genome. Nat Genet 2019;51:541–7. 30804557 10.1038/s41588-019-0356-4 PMC6882729

[25] Liston A, Wei N, Tennessen JA, et al. Revisiting the origin of octoploid strawberry. Nat Genet 2020;52:2–04. 31844319 10.1038/s41588-019-0543-3

[26] Edger PP, McKain MR, Yocca AE, et al. Reply to: revisiting the origin of octoploid strawberry. Nat Genet 2020;52:5–07. 31844320 10.1038/s41588-019-0544-2 PMC6960091

[27] Feng C, Wang J, Harris AJ, et al. Tracing the diploid ancestry of the cultivated octoploid strawberry. Mol Biol Evol 2021;38:478–85. 32941604 10.1093/molbev/msaa238 PMC7826170

[28] Marcussen T, Sandve SR, Heier L, et al. Ancient hybridizations among the ancestral genomes of bread wheat. Science 2014;345:1250092. 25035499 10.1126/science.1250092

[29] Zhang C, Mirarab S. ASTRAL-pro 2: ultrafast species tree reconstruction from multi-copy gene family trees. Bioinformatics 2022;38:4949–50. 36094339 10.1093/bioinformatics/btac620

[30] Kamal N, Tsardakas Renhuldt N, Bentzer J, et al. The mosaic oat genome gives insights into a uniquely healthy cereal crop. Nature 2022;606:113–9. 35585233 10.1038/s41586-022-04732-y PMC9159951

[31] Degnan J, Rosenberg N. Gene tree discordance, phylogenetic inference and the multispecies coalescent. Trends Ecol Evol 2009;24:332–40. 19307040 10.1016/j.tree.2009.01.009

[32] Mirarab S, Warnow T. ASTRAL-II: coalescent-based species tree estimation with many hundreds of taxa and thousands of genes. Bioinformatics 2015;31:i44–52. 26072508 10.1093/bioinformatics/btv234 PMC4765870

[33] Schnable JC, Springer NM, Freeling M. Differentiation of the maize subgenomes by genome dominance and both ancient and ongoing gene loss. Proc Natl Acad Sci U S A 2011;108:4069–74. 21368132 10.1073/pnas.1101368108 PMC3053962

[34] Wang X, Wang H, Wang J, et al. The genome of the mesopolyploid crop species *Brassica rapa*. Nat Genet 2011;43:1035–9. 21873998 10.1038/ng.919

[35] Deb SK, Edger PP, Pires JC, McKain MR. Patterns, mechanisms, and consequences of homoeologous exchange in allopolyploid angiosperms: a genomic and epigenomic perspective. New Phytol 2023;238:2284–304. 37010081 10.1111/nph.18927

[36] Aköz G, Nordborg M. The *Aquilegia* genome reveals a hybrid origin of core eudicots. Genome Biol 2019;20:256. 31779695 10.1186/s13059-019-1888-8 PMC6883705

[37] Zhong S, Li B, Chen W, et al. The chromosome-level genome of *Akebia trifoliata* as an important resource to study plant evolution and environmental adaptation in the Cretaceous. Plant J 2022;112:1316–30. 36305286 10.1111/tpj.16011

[38] Shi T, Chen J. A reappraisal of the phylogenetic placement of the *Aquilegia* whole-genome duplication. Genome Biol 2020;21:295. 33292440 10.1186/s13059-020-02212-y PMC7722308

[39] Liu P, Zhang X, Mao J, et al. The *Tetracentron* genome provides insight into the early evolution of eudicots and the formation of vessel elements. Genome Biol 2020;21:291. 33267872 10.1186/s13059-020-02198-7 PMC7709256

[40] Li M, Yang Y, Xu R, et al. A chromosome-level genome assembly for the tertiary relict plant *Tetracentron sinense* Oliv. (Trochodendraceae). Mol Ecol Resour 2021;21:1186–99. 33486895 10.1111/1755-0998.13334

[41] Chanderbali AS, Jin L, Xu Q, et al. *Buxus* and *Tetracentron* genomes help resolve eudicot genome history. Nat Commun 2022;13:643. 35110570 10.1038/s41467-022-28312-w PMC8810787

[42] Wang Z, Li Y, Sun P, et al. A high-quality *Buxus austro-yunnanensis* (Buxales) genome provides new insights into karyotype evolution in early eudicots. BMC Biol 2022;20:216. 36195948 10.1186/s12915-022-01420-1 PMC9533543

[43] Maccaferri M, Harris NS, Twardziok SO, et al. Durum wheat genome highlights past domestication signatures and future improvement targets. Nat Genet 2019;51:885–95. 30962619 10.1038/s41588-019-0381-3

[44] Zhu T, Wang L, Rimbert H, et al. Optical maps refine the bread wheat *Triticum aestivum* cv. Chinese Spring genome assembly. Plant J 2021;107:303–14. 33893684 10.1111/tpj.15289 PMC8360199

[45] Ling H, Ma B, Shi X, et al. Genome sequence of the progenitor of wheat A subgenome *Triticum urartu*. Nature 2018;557:424–8. 29743678 10.1038/s41586-018-0108-0 PMC6784869

[46] Li L, Zhang Z, Wang Z, et al. Genome sequences of five Sitopsis species of *Aegilops* and the origin of polyploid wheat B subgenome. Mol Plant 2022;15:488–503. 34979290 10.1016/j.molp.2021.12.019

[47] Zimin AV, Puiu D, Luo MC, et al. Hybrid assembly of the large and highly repetitive genome of *Aegilops tauschii*, a progenitor of bread wheat, with the MaSuRCA mega-reads algorithm. Genome Res 2017;27:787–92. 28130360 10.1101/gr.213405.116 PMC5411773

[48] Maughan PJ, Lee R, Walstead R, et al. Genomic insights from the first chromosome-scale assemblies of oat (*Avena* spp.) diploid species. BMC Biol 2019;17:92. 31757219 10.1186/s12915-019-0712-y PMC6874827

[49] Mayer KF, Waugh R, Langridge P, et al. A physical, genetic and functional sequence assembly of the barley genome. Nature 2012;491:711–6. 23075845 10.1038/nature11543

[50] Yang X, Gao S, Guo L, et al. Three chromosome-scale *Papaver* genomes reveal punctuated patchwork evolution of the morphinan and noscapine biosynthesis pathway. Nat Commun 2021;12:6030. 34654815 10.1038/s41467-021-26330-8 PMC8521590

[51] Kang L, Qian L, Zheng M, et al. Genomic insights into the origin, domestication and diversification of *Brassica juncea*. Nat Genet 2021;53:1392–402. 34493868 10.1038/s41588-021-00922-y PMC8423626

[52] Song J, Guan Z, Hu J, et al. Eight high-quality genomes reveal pan-genome architecture and ecotype differentiation of *Brassica napus*. Nat Plants 2020;6:34–45. 31932676 10.1038/s41477-019-0577-7 PMC6965005

[53] Song X, Wei Y, Xiao D, et al. *Brassica carinata* genome characterization clarifies U's triangle model of evolution and polyploidy in *Brassica*. Plant Physiol 2021;186:388–406. 33599732 10.1093/plphys/kiab048 PMC8154070

[54] Belser C, Istace B, Denis E, et al. Chromosome-scale assemblies of plant genomes using nanopore long reads and optical maps. Nat Plants 2018;4:879–87. 30390080 10.1038/s41477-018-0289-4

[55] Zhang L, Cai X, Wu J, et al. Improved *Brassica rapa* reference genome by single-molecule sequencing and chromosome conformation capture technologies. Hortic Res 2018;5:50. 30131865 10.1038/s41438-018-0071-9 PMC6092429

[56] Yang T, Cai B, Jia Z, et al. *Sinapis* genomes provide insights into whole-genome triplication and divergence patterns within tribe Brassiceae. Plant J 2023;113:246–61. 36424891 10.1111/tpj.16043

[57] Fan Z, Tieman DM, Knapp SJ, et al. A multi-omics framework reveals strawberry flavor genes and their regulatory elements. New Phytol 2022;236:1089–107. 35916073 10.1111/nph.18416 PMC9805237

[58] Hirakawa H, Shirasawa K, Kosugi S, et al. Dissection of the octoploid strawberry genome by deep sequencing of the genomes of *Fragaria* species. DNA Res 2014;21:169–81. 24282021 10.1093/dnares/dst049 PMC3989489

[59] Davik J, Røen D, Lysøe E, et al. A chromosome-level genome sequence assembly of the red raspberry (*Rubus idaeus* L.). PloS One 2022;17:e265096. 10.1371/journal.pone.0265096 PMC8926247 35294470

[60] Alonge M, Lebeigle L, Kirsche M, et al. Automated assembly scaffolding using RagTag elevates a new tomato system for high-throughput genome editing. Genome Biol 2022;23:258. 36522651 10.1186/s13059-022-02823-7 PMC9753292

[61] Buchfink B, Xie C, Huson DH. Fast and sensitive protein alignment using DIAMOND. Nat Methods 2015;12:59–60. 25402007 10.1038/nmeth.3176

[62] Minh BQ, Schmidt HA, Chernomor O, et al. IQ-TREE 2: new models and efficient methods for phylogenetic inference in the genomic era. Mol Biol Evol 2020;37:1530–4. 32011700 10.1093/molbev/msaa015 PMC7182206

[63] Junier T, Zdobnov EM. The Newick utilities: high-throughput phylogenetic tree processing in the Unix shell. Bioinformatics 2010;26:1669–70. 20472542 10.1093/bioinformatics/btq243 PMC2887050

