# Supplementary material for: Subgenome phasing for complex allopolyploidy: case-based benchmarking and recommendations
Source: Brief Bioinform. 2024 Jan 6;25(1):bbad513. doi: 10.1093/bib/bbad513 (PMC10772947; doi:10.1093/bib/bbad513)
Supplement: supplement-wgdiwork-11_18_bbad513 [file supplement-wgdiwork-11_18_bbad513.pdf]

Subgenome Phasing for Complex Allopolyploidy: Case-based Benchmarking and Recommendations

Zhang et al.

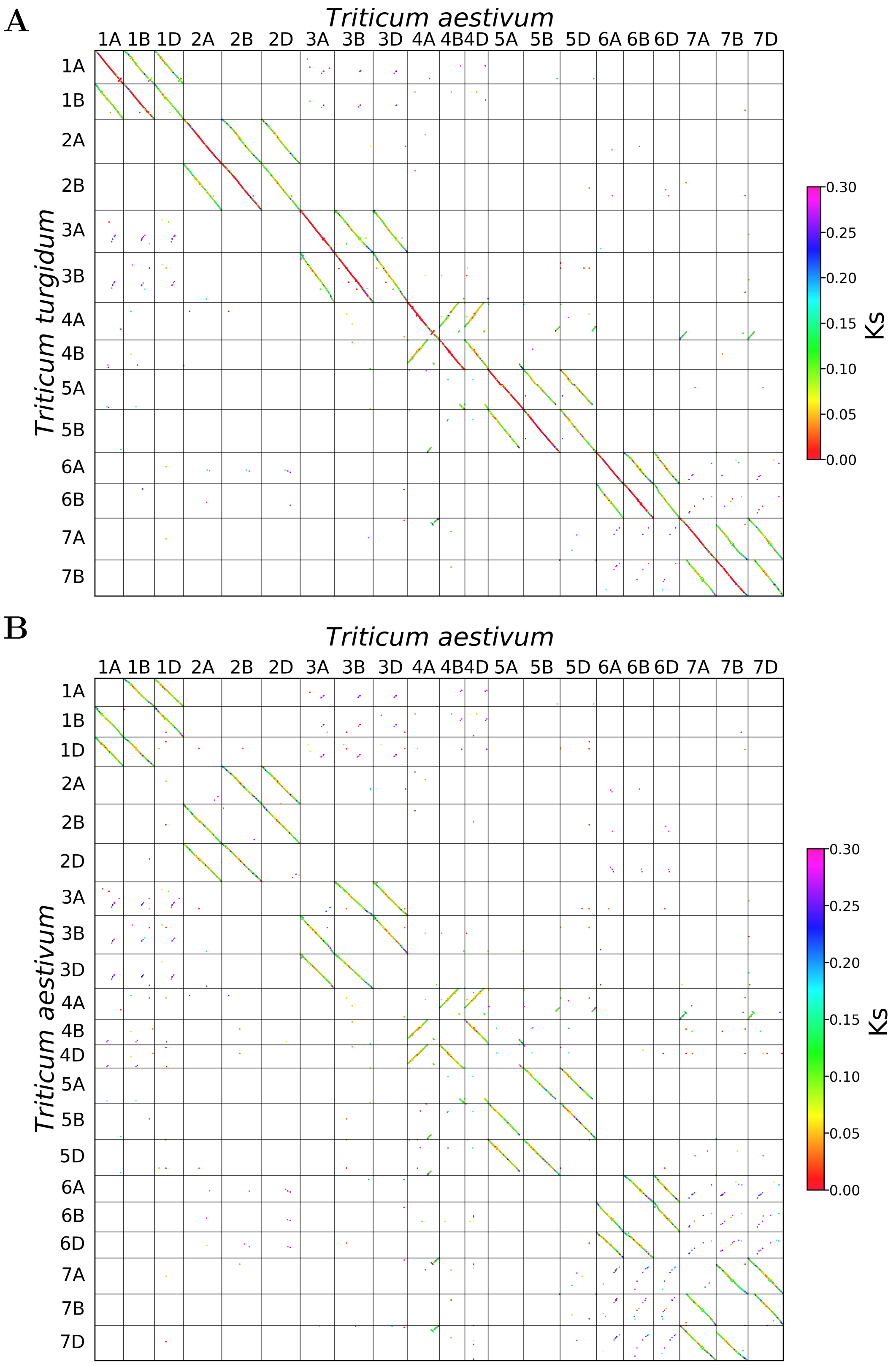

Figure S1. Dot plots colored with Ks distance for syntenic gene pairs. (**A**) Synteny between allohexaploid wheat (AABBDD, *Triticum aestivum*) and allotetraploid wheat (AABB, *Triticum turgidum*). (**B**) Synteny within allohexaploid wheat (AABBDD, *Triticum aestivum*). The smaller the Ks value, the higher the similarity between the genes.

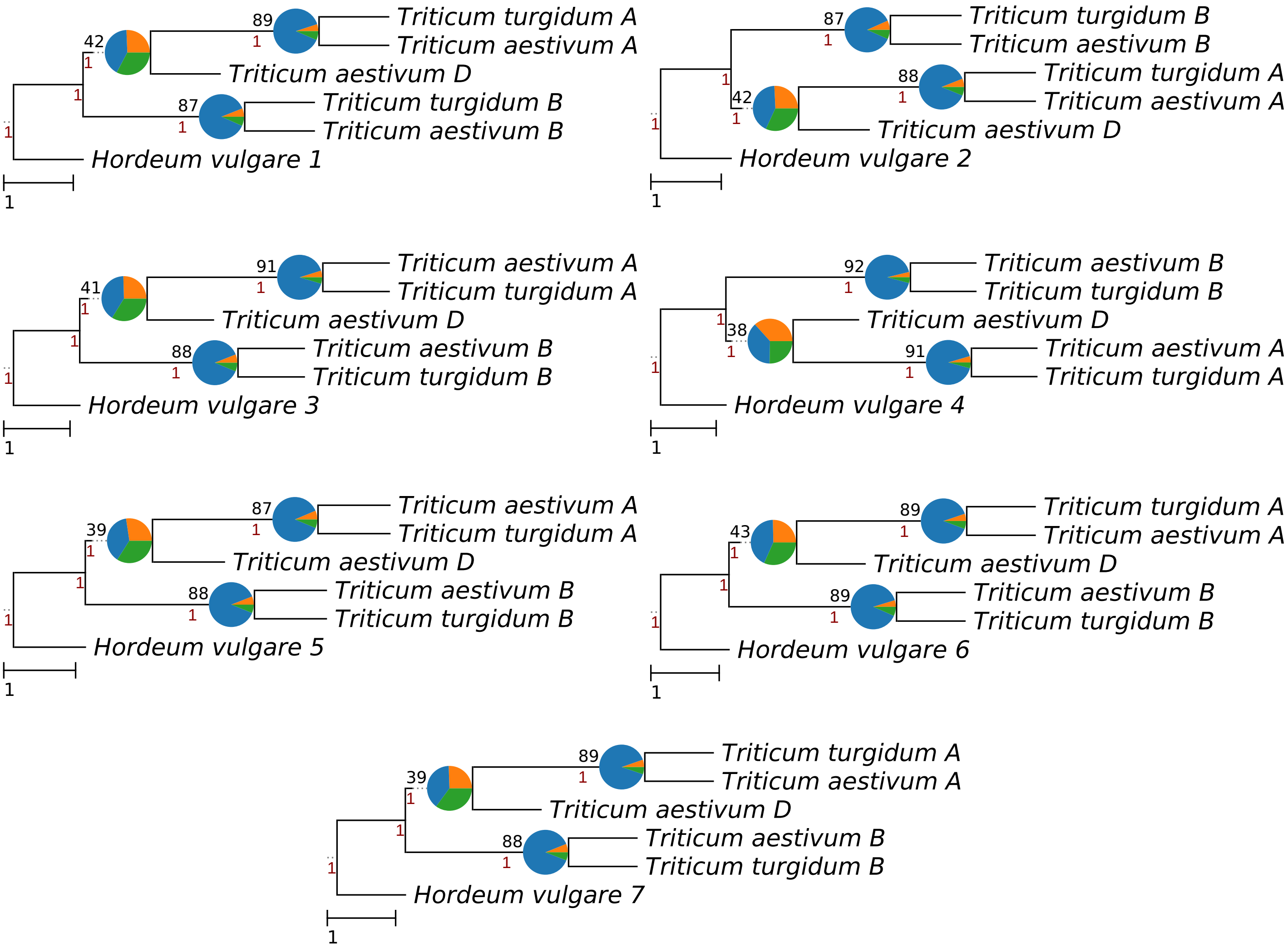

Figure S2. The phylogenetic tree reconstructed from each chromosome of the three subgenomes of allohexaploid wheat (AABBDD, *Triticum aestivum*), and two subgenomes of allotetraploid wheat (AABB, *Triticum turgidum*). *Hordeum vulgare* served as the outgroup. Numbers above the branches represent the percentages of concordance between gene trees and species/subgenome tree, and numbers below the branches represent the local posterior probabilities calculated in ASTRAL. Pie plots at the nodes represent the percentages of three different gene tree topologies (q1, q2 and q3) calculated in ASTRAL. Bar, 1.0 coalescent units.

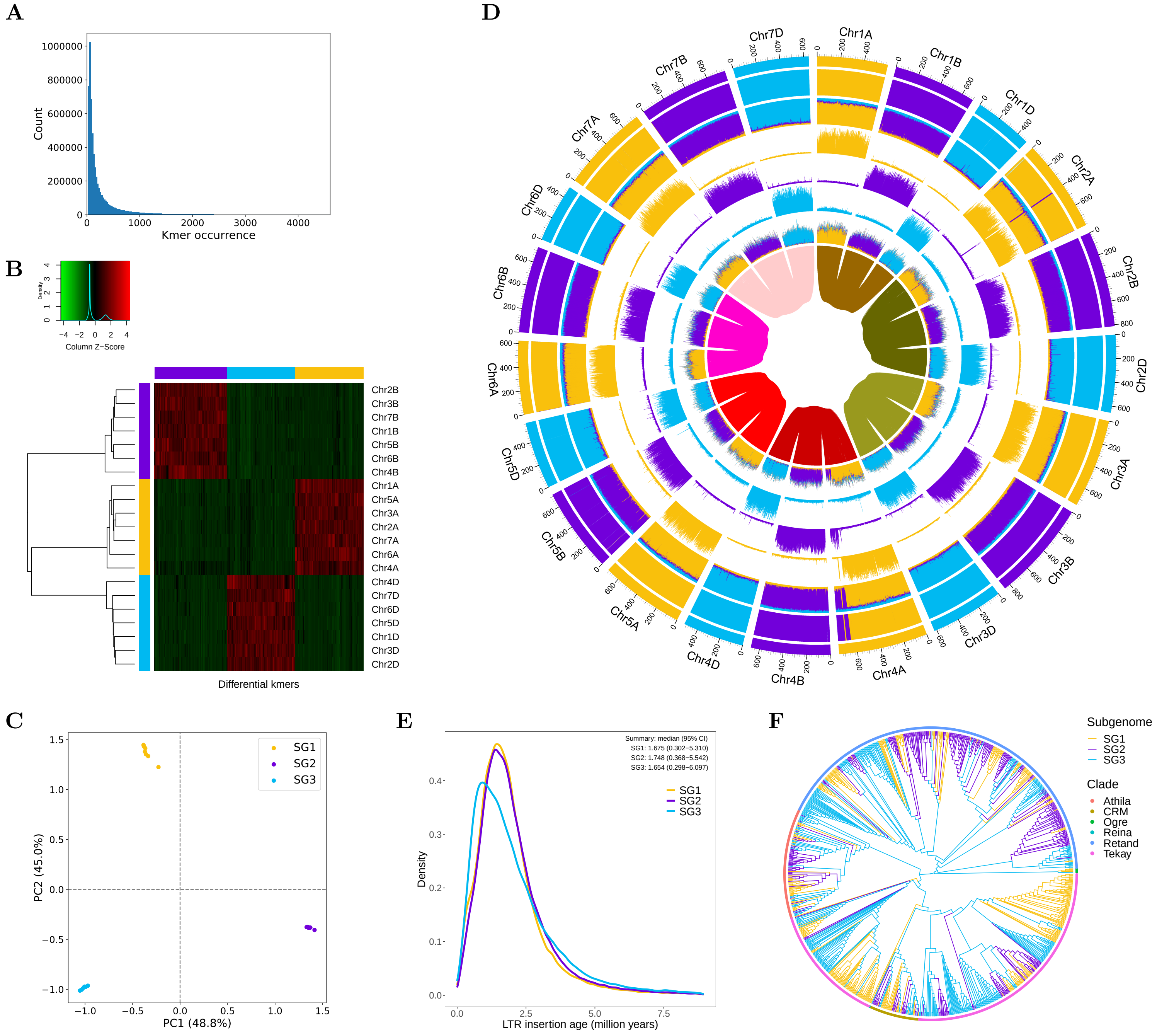

Figure S3. Subgenome phasing of *Triticum aestivum* using SubPhaser. (**A**) Frequency distribution of differential 15-mers among the homoeologous chromosomes. (**B**) Unsupervised hierarchical clustering. The horizontal color bar at the top of the axis indicates to which subgenome the *k*-mer is specific. The vertical color bar on the left of the axis indicates to which subgenome the chromosome is assigned. The heatmap indicates the *Z*-scaled relative abundance of *k*-mers; the larger the *Z* score, the higher the relative abundance of a *k*-mer. (**C**) Principal component analysis (PCA) of differential 15-mers. (**D**) Chromosomal characteristics. From outer to inner circles (1–8): (1) Subgenome assignments based on the *k*-Means algorithm. (2) Significant enrichment of subgenome-specific *k*-mers. Subgenome-specific k-mers with same color as the subgenome are significantly enriched. White areas are not significantly enriched. (3) Normalized proportion (relative) of subgenome-specific *k*-mers. (4–6) Counts (absolute) of each subgenome-specific *k*-mer set. (7) Density of long terminal repeat retrotransposons (LTR-RTs). If the color is consistent with that of the subgenome, it indicates that the LTR-RTs are significantly enriched in those subgenome-specific *k*-mers. Gray indicates non-specific LTR-RTs. (8) Homoeologous blocks. All statistics (2–7) were computed in sliding windows of 1 Mb. (**E**) Insertion times of subgenome-specific LTR-RTs. The 95% confidence interval (CI) is marked in the upper right corner and was used to predict the insertion time boundaries of LTR-RTs on the subgenome. (**F**) Phylogenetic tree of up to 1,000 *Gypsy* LTR-RTs randomly selected from the subgenome-specific LTR-RTs. The branches are colored by subgenome and the terminal nodes are colored by clade. Clades were classified using TEsorter. (B–F) Colors are consistent for subgenomes. SG1 = subgenome A, SG2 =subgenome B, SG3 = subgenome D. The results were adopted from Jia et al (2022).

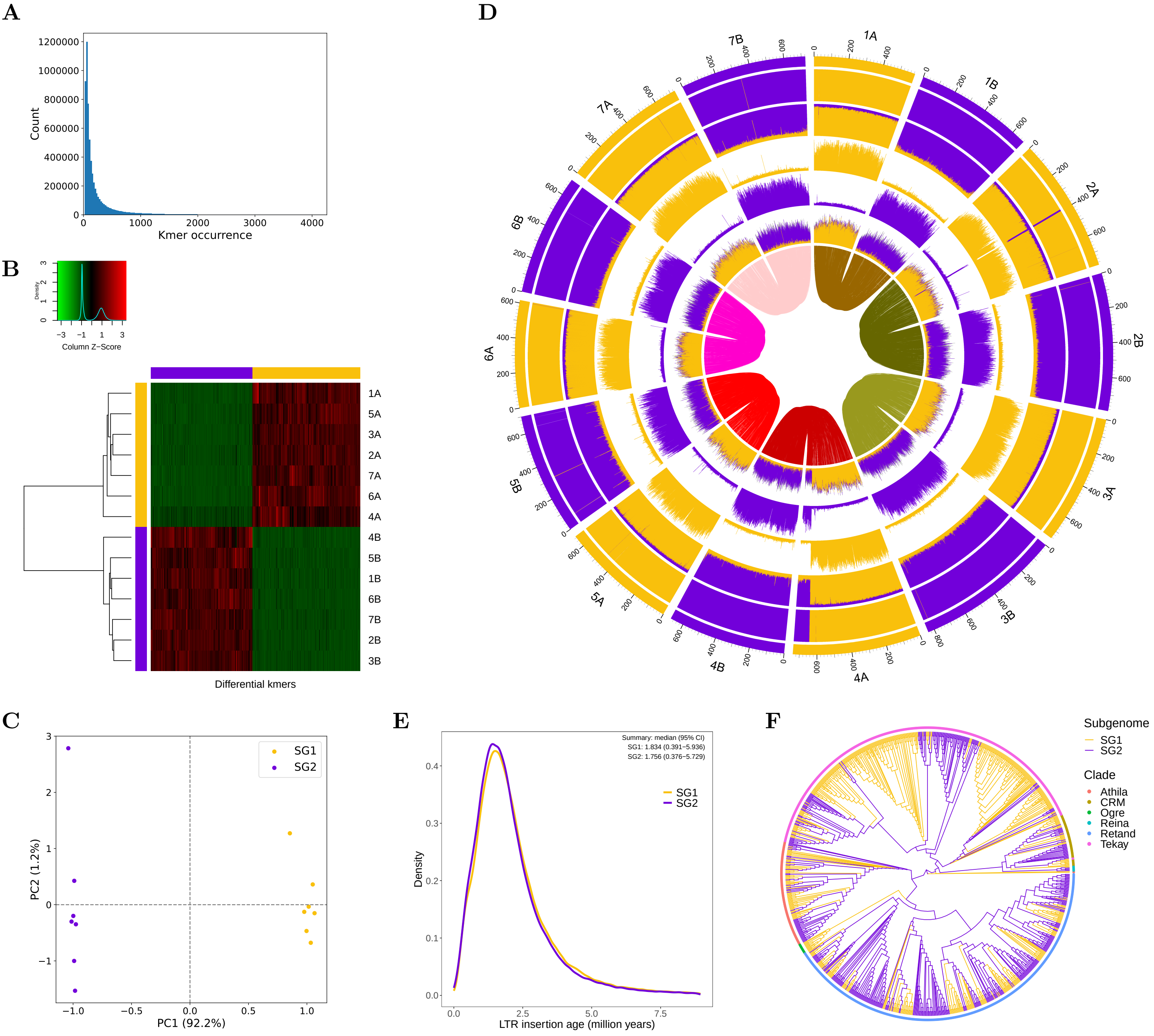

Figure S4. Subgenome phasing of *Triticum turgidum* using SubPhaser. (**A**) Frequency distribution of differential 15-mers among the homoeologous chromosomes. (**B**) Unsupervised hierarchical clustering. The horizontal color bar at the top of the axis indicates to which subgenome the k-mer is specific. The vertical color bar on the left of the axis indicates to which subgenome the chromosome is assigned. The heatmap indicates the Z-scaled relative abundance of k-mers; the larger the Z score, the higher the relative abundance of a k-mer. (**C**) Principal component analysis (PCA) of differential 15-mers. (**D**) Chromosomal characteristics. From outer to inner circles (1–7): (1) Subgenome assignments based on the k-Means algorithm. (2) Significant enrichment of subgenome-specific k-mers. Subgenome-specific k-mers with the same color as the subgenome are significantly enriched. White areas are not significantly enriched. (3) Normalized proportion (relative) of subgenome-specific k-mers. (4–5) Counts (absolute) of each subgenome-specific k-mer set. (6) Density of long terminal repeat retrotransposons (LTR-RTs). If the color is the same as that of the subgenome, the LTR-RTs are significantly enriched in those subgenome-specific k-mers. Gray indicates non-specific LTR-RTs. (7) Homoeologous blocks. All statistics (2–6) were computed in sliding windows of 1 Mb. (**E**) Insertion times of subgenome-specific LTR-RTs. The 95% confidence interval (CI) is marked in the upper right corner and was used to predict the insertion time boundaries of LTR-RTs on the subgenome. (**F**) Phylogenetic tree of up to 1,000 *Gypsy* LTR-RTs randomly selected from the subgenome-specific LTR-RTs. The branches are colored by subgenome and the terminal nodes are colored by clade. Clades were classified using TEsorter. (B–F) Colors are consistent for subgenomes. SG1 = subgenome A, SG2 = subgenome B. The results were adopted from Jia et al (2022).

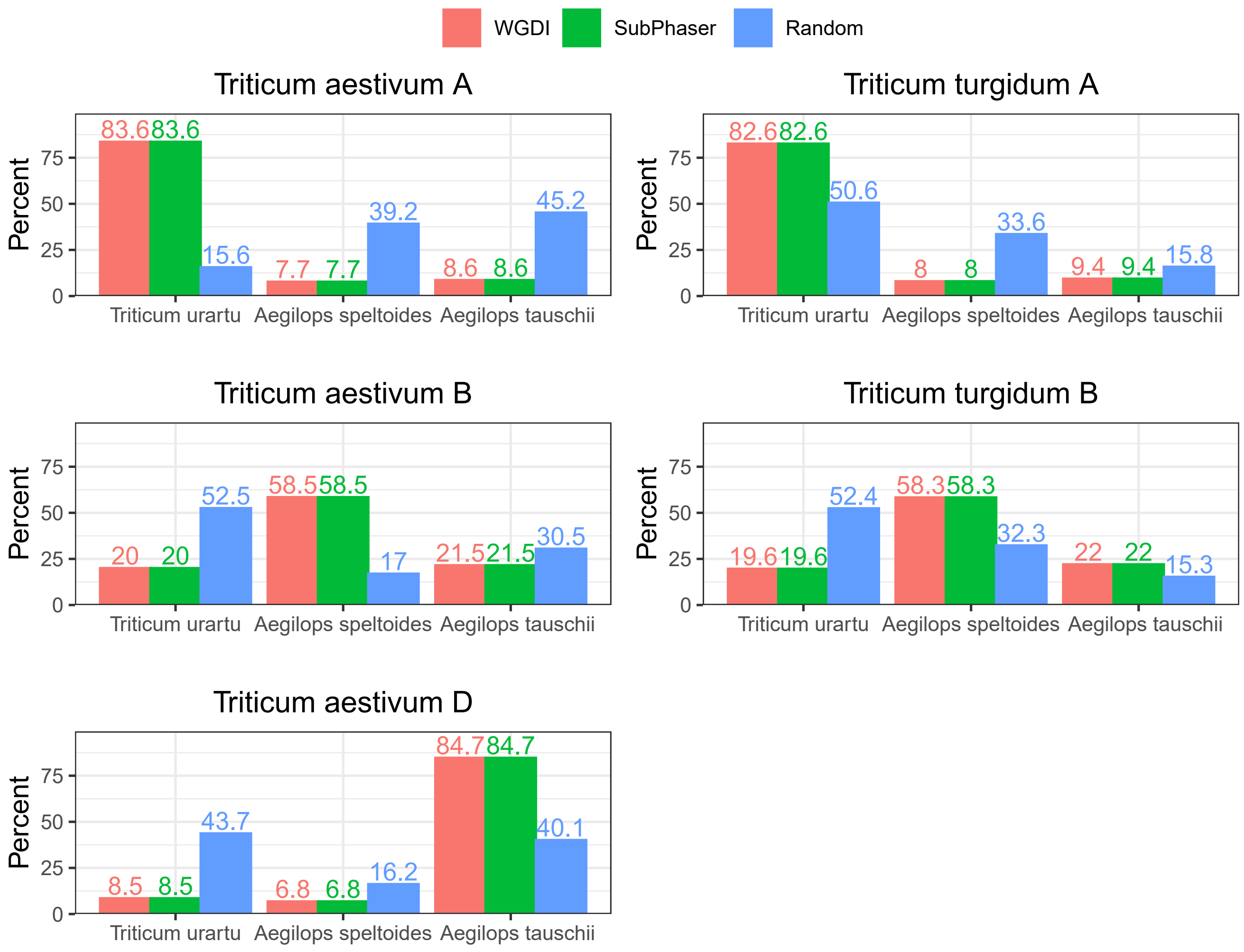

Figure S5. Comparison of similarity between polyploid subgenomes and diploid potential progenitors in the wheat case. Y-axes, percent of best hits from genes of one subgenome to those of multiple diploids. Red, WGDI; green, SubPhaser; blue, random sorting.

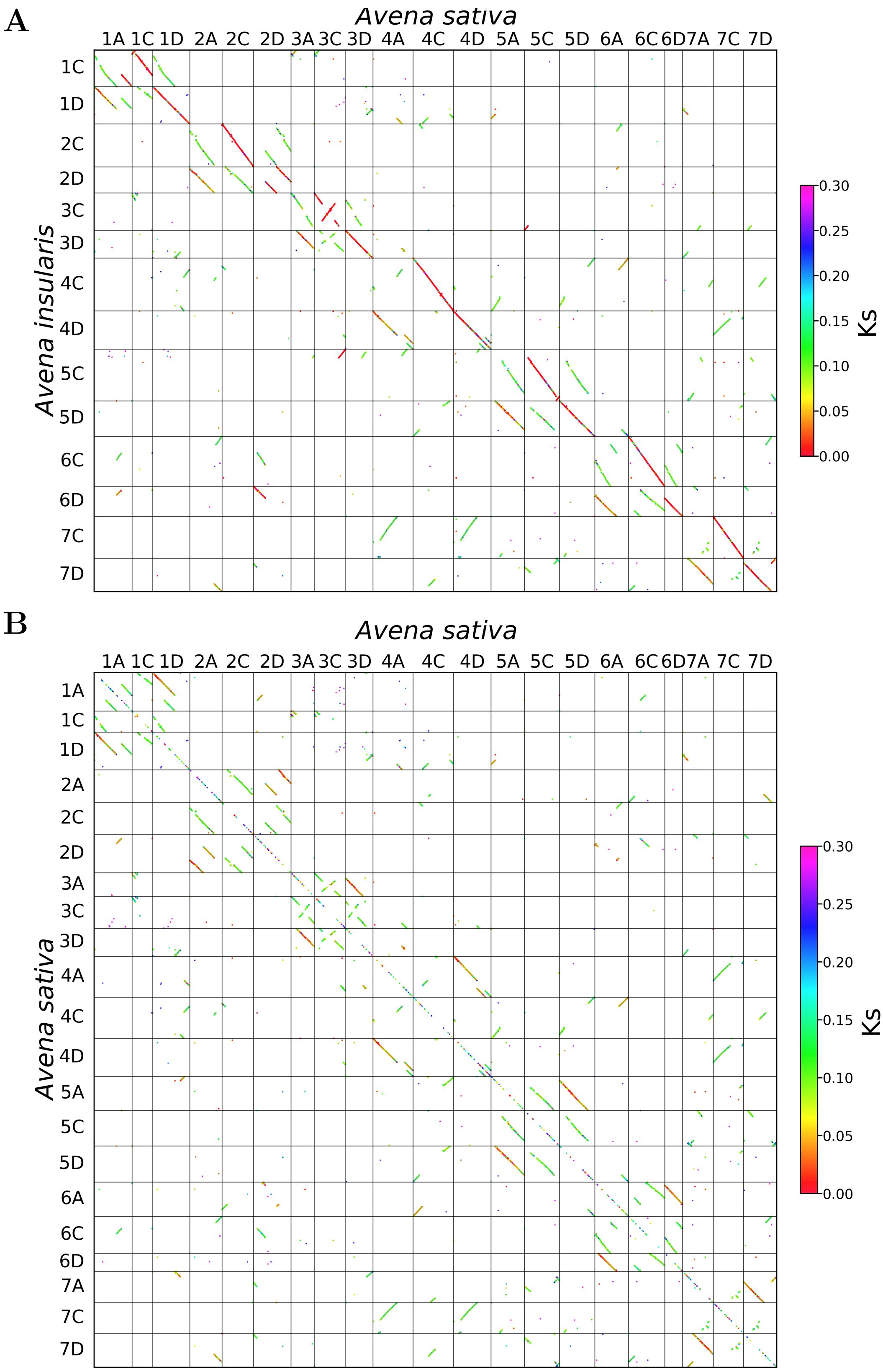

Figure S6. Dot plots colored by Ks distance for syntenic gene pairs (**A**) Synteny between allotetraploid oat (CCDD, *Avena insularis*) and allohexaploid oat (AACCDD, *Avena sativa*). (**B**) Synteny within allohexaploid oat (AACCDD, *Avena sativa*).

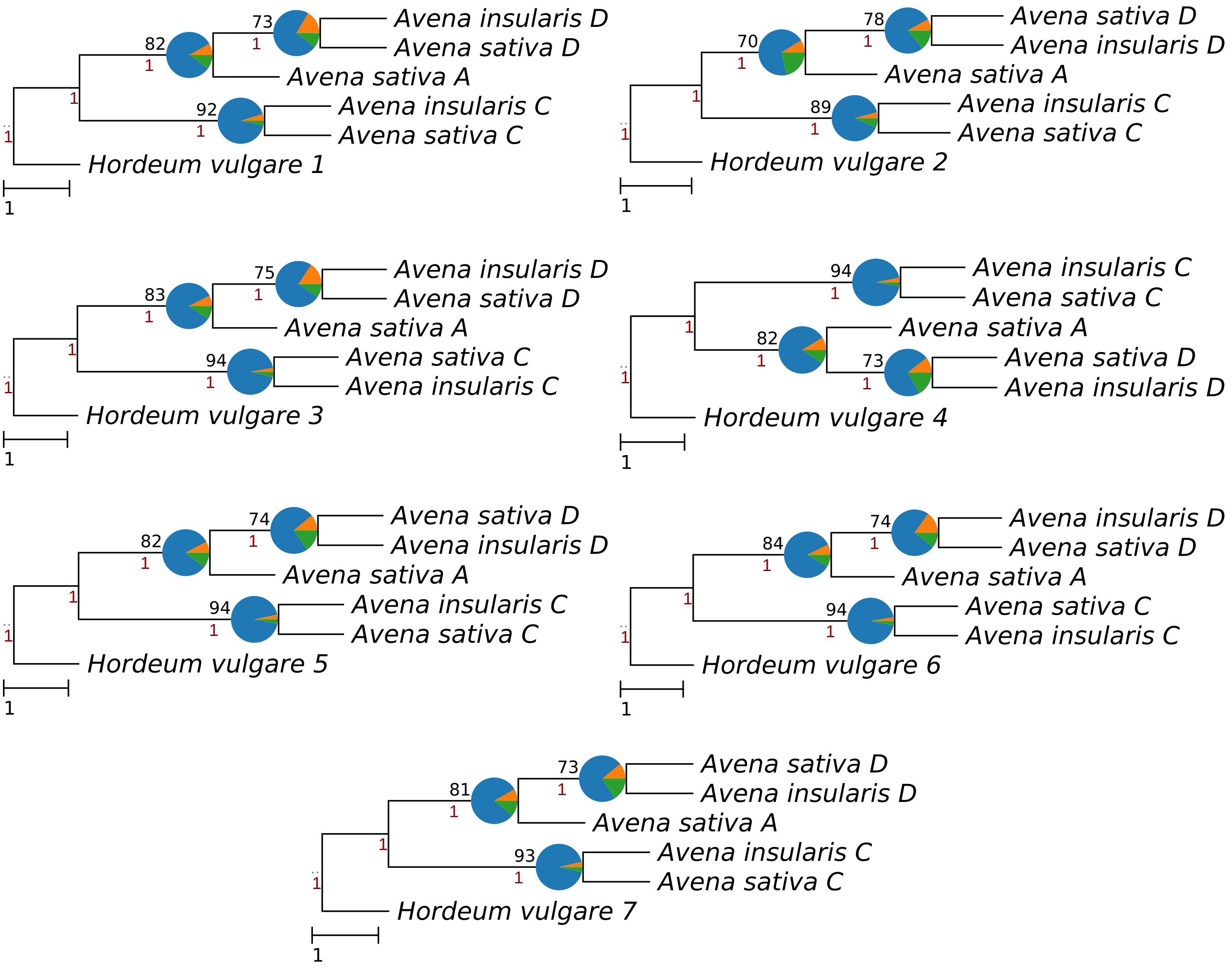

Figure S7. The phylogenetic tree reconstructed from each chromosome of the three subgenomes of the allohexaploid oat (AACCDD, *Avena sativa*), and two subgenomes of the allotetraploid oat (CCDD, *Avena insularis*). *Hordeum vulgare* served as the outgroup. Numbers above the branches represent the percentages of concordance between the gene and species/subgenome trees, and numbers below the branches represent the local posterior probabilities calculated in ASTRAL. Pie plots at the nodes represent the percentages of three gene tree topologies (q1, q2 and q3) calculated in ASTRAL. Bar, 1.0 coalescent units.

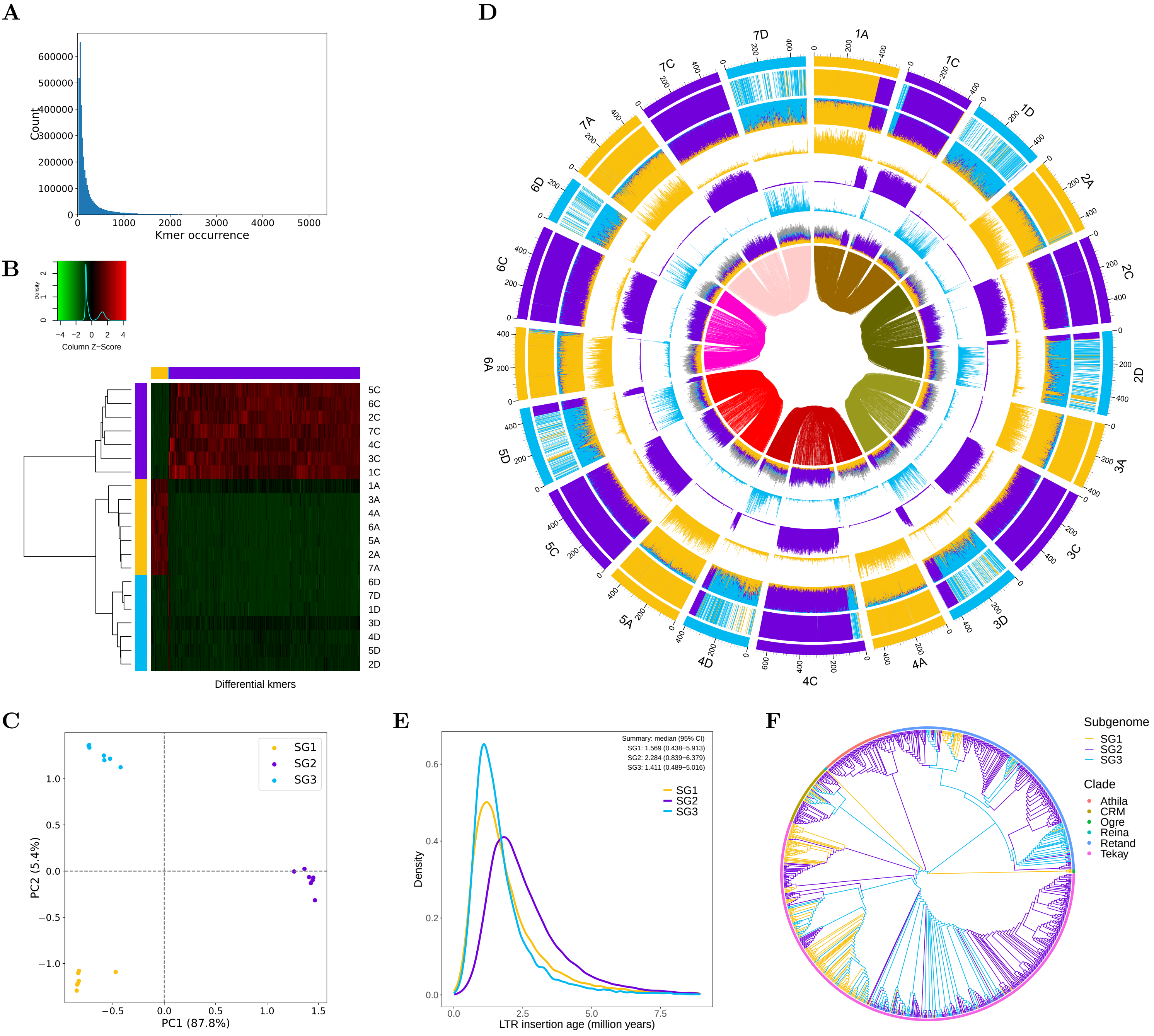

Figure S8. Subgenome phasing of *Avena sativa* using SubPhaser. (**A**) Frequency distribution of differential 15-mers among the homoeologous chromosomes. (**B**) Unsupervised hierarchical clustering. The horizontal color bar at the top of the axis indicates to which subgenome the *k*-mer is specific. The vertical color bar on the left of the axis indicates to which subgenome the chromosome is assigned. The heatmap indicates the *Z*-scaled relative abundance of *k*-mers; the larger the *Z* score, the higher the relative abundance of a *k*-mer. (**C**) Principal component analysis (PCA) of differential 15-mers. (**D**) Chromosomal characteristics. From outer to inner circles (1–8): (1) Subgenome assignments based on the *k*-Means algorithm. (2) Significant enrichment of subgenome-specific *k*-mers. Subgenome-specific k-mers with the same color as the subgenome are significantly enriched. White areas are not significantly enriched. (3) Normalized proportion (relative) of subgenome-specific *k*-mers. (4–6) Counts (absolute) of each subgenome-specific *k*-mer set. (7) Density of long terminal repeat retrotransposons (LTR-RTs). If the color is consistent with that of the subgenome, it indicates that LTR-RTs are significantly enriched in those subgenome-specific *k*-mers. Gray indicates non-specific LTR-RTs. (8) Homoeologous blocks. All statistics (2–7) were computed in sliding windows of 1 Mb. (**E**) Insertion times of subgenome-specific LTR-RTs. The 95% confidence interval (CI) is marked in the upper right corner and was used to predict the insertion time boundaries of LTR-RTs on the subgenome. (**F**) Phylogenetic tree of up to 1,000 *Gypsy* LTR-RTs randomly selected from the subgenome-specific LTR-RTs. The branches are colored by subgenome and the terminal nodes are colored by clade. Clades were classified using TEsorter. (B–F) Colors are consistent for subgenomes. SG1 = subgenome A, SG2 = subgenome C, SG3 = subgenome D.

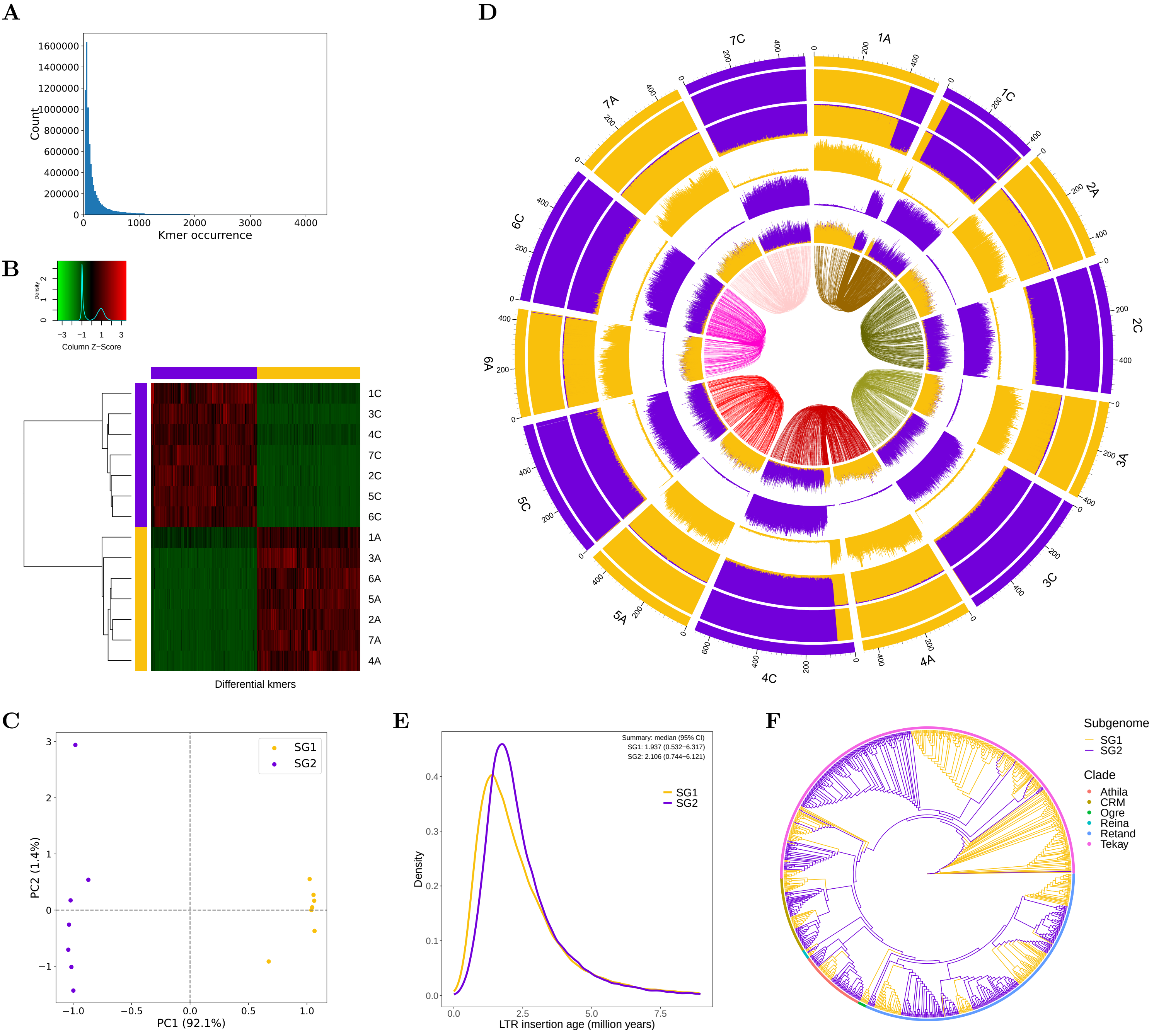

Figure S9. Subgenome phasing of *Avena sativa* A + C subgenomes using SubPhaser. (**A**) Frequency distribution of differential 15-mers among the homoeologous chromosomes. (**B**) Unsupervised hierarchical clustering. The horizontal color bar at the top of the axis indicates to which subgenome the *k*-mer is specific. The vertical color bar on the left of the axis indicates to which subgenome the chromosome is assigned. The heatmap indicates the *Z*-scaled relative abundance of *k*-mers; the larger the *Z* score, the higher the relative abundance of a *k*-mer. (**C**) Principal component analysis (PCA) of differential 15-mers. (**D**) Chromosomal characteristics. From outer to inner circles (1–7): (1) Subgenome assignments based on the *k*-Means algorithm. (2) Significant enrichment of subgenome-specific *k*-mers. Subgenome-specific k-mers with the same color as the subgenome are significantly enriched *k*-mers. White areas are not significantly enriched. (3) Normalized proportion (relative) of subgenome-specific *k*-mers. (4–5) Counts (absolute) of each subgenome-specific *k*-mer set. (6) Density of long terminal repeat retrotransposons (LTR-RTs). If the color is consistent with that of the subgenome, it indicates that the LTR-RTs are significantly enriched in those subgenome-specific *k*-mers. Gray indicates non-specific LTR-RTs. (7) Homoeologous blocks. All statistics (2–6) were computed in sliding windows of 1 Mb. (**E**) Insertion times of subgenome-specific LTR-RTs. The 95% confidence interval (CI) is marked in the upper right corner and was used to predict the insertion time boundaries of LTR-RTs on the subgenome. (**F**) Phylogenetic tree of up to 1,000 *Gypsy* LTR-RTs randomly selected from the subgenome-specific LTR-RTs. The branches are colored by subgenome and the terminal nodes are colored by clade. Clades were classified using TEsorter. (B–F) Colors are consistent for subgenomes. SG1 = subgenome A, SG2 = subgenome C.

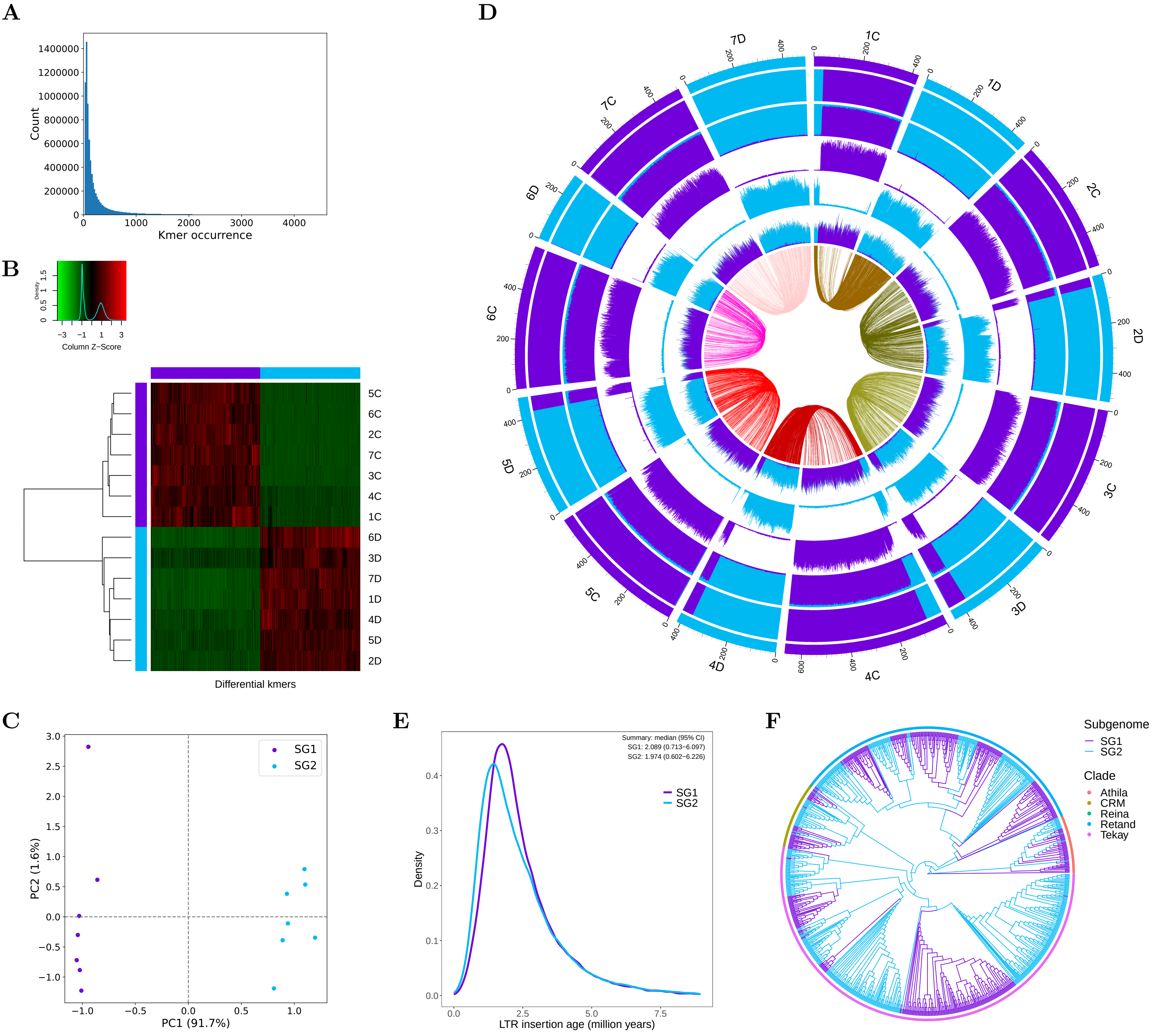

Figure S10. Subgenome phasing of *Avena sativa* D + C subgenomes using SubPhaser. (**A**) Frequency distribution of differential 15-mers among the homoeologous chromosomes. (**B**) Unsupervised hierarchical clustering. The horizontal color bar at the top of the axis indicates to which subgenome the k-mer is specific. The vertical color bar on the left of the axis indicates to which subgenome the chromosome is assigned. The heatmap indicates the Z-scaled relative abundance of k-mers; the larger the Z score, the higher the relative abundance of a k-mer. (**C**) Principal component analysis (PCA) of differential 15-mers. (**D**) Chromosomal characteristics. From outer to inner circles (1–7): (1) Subgenome assignments based on the k-Means algorithm. (2) Significant enrichment of subgenome-specific k-mers. Subgenome-specific k-mers with the same color as the subgenome are significantly enriched. White areas are not significantly enriched. (3) Normalized proportion (relative) of subgenome-specific k-mers. (4–5) Counts (absolute) of each subgenome-specific k-mer set. (6) Density of long terminal repeat retrotransposons (LTR-RTs). If the color is consistent with that of the subgenome, it indicates that the LTR-RTs are significantly enriched in those subgenome-specific k-mers. Gray indicates non-specific LTR-RTs. (7) Homoeologous blocks. All statistics (2–6) were computed in sliding windows of 1 Mb. (**E**) Insertion times of subgenome-specific LTR-RTs. The 95% confidence interval (CI) is marked in the upper right corner and was used to predict the insertion time boundaries of LTR-RTs on the subgenome. (**F**) Phylogenetic tree of up to 1,000 *Gypsy* LTR-RTs randomly selected from the subgenome-specific LTR-RTs. The branches are colored by subgenome and the terminal nodes are colored by clade. Clades were classified using TEsorter. (B–F) Colors are consistent for subgenomes. SG1 = subgenome C, SG2 = subgenome D.

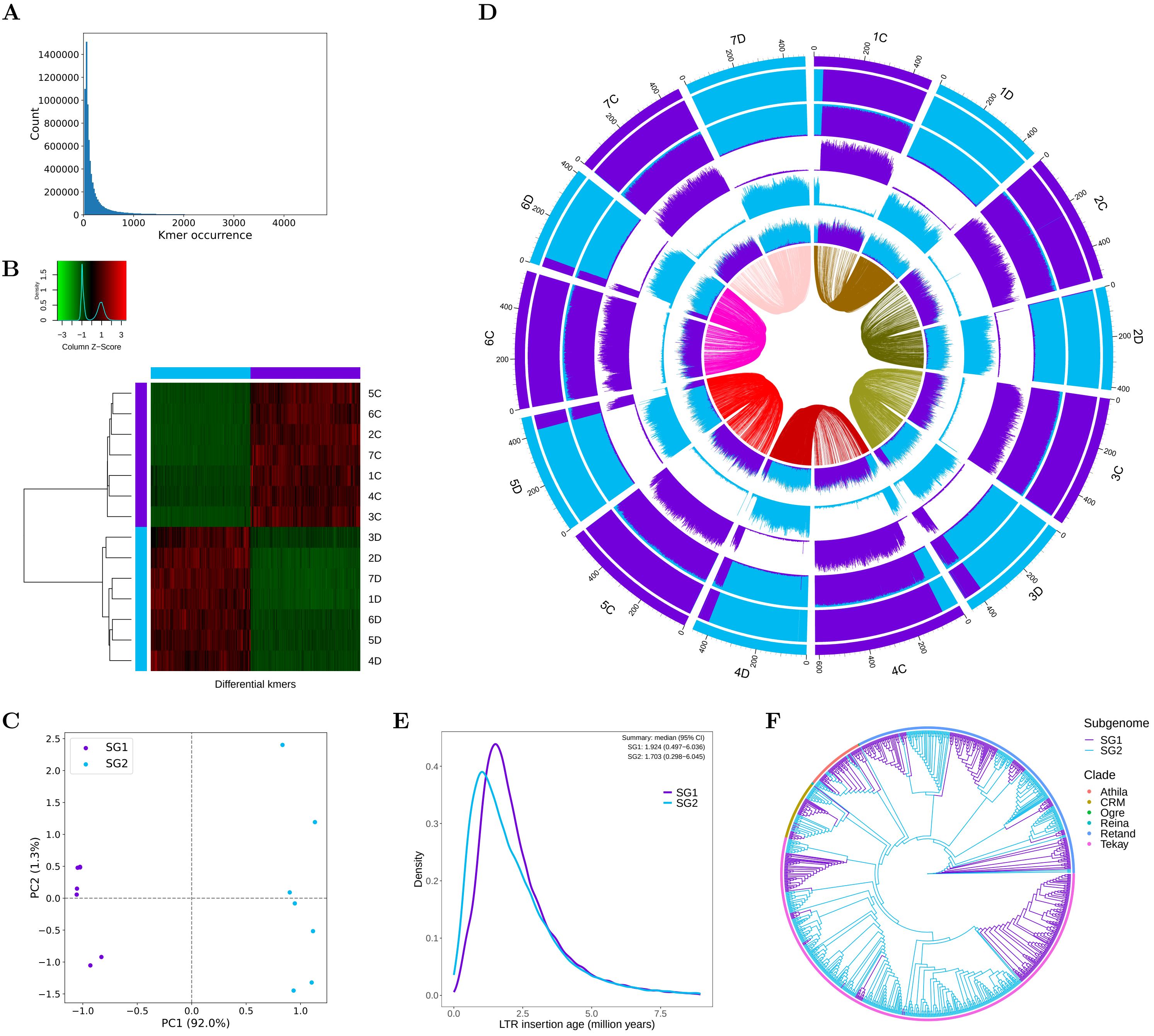

Figure S11. Subgenome phasing of *Avena insularis* using SubPhaser. (**A**) Frequency distribution of differential 15-mers among the homoeologous chromosomes. (**B**) Unsupervised hierarchical clustering. The horizontal color bar at the top of the axis indicates to which subgenome the *k*-mer is specific. The vertical color bar on the left of the axis indicates to which subgenome the chromosome is assigned. The heatmap indicates the *Z*-scaled relative abundance of *k*-mers; the larger the *Z* score, the higher the relative abundance of a *k*-mer. (**C**) Principal component analysis (PCA) of differential 15-mers. (**D**) Chromosomal characteristics. From outer to inner circles (1–7): (1) Subgenome assignments based on the *k*-Means algorithm. (2) Significant enrichment of subgenome-specific *k*-mers. Subgenome-specific k-mers with the same color as the subgenome are significantly enriched. White areas are not significantly enriched. (3) Normalized proportion (relative) of subgenome-specific *k*-mers. (4–5) Counts (absolute) of each subgenome-specific *k*-mer set. (6) Density of long terminal repeat retrotransposons (LTR-RTs). If the color is consistent with that of the subgenome, it indicates that the LTR-RTs are significantly enriched in those subgenome-specific *k*-mers. Gray indicates non-specific LTR-RTs. (7) Homoeologous blocks. All statistics (2–6) were computed in sliding windows of 1 Mb. (**E**) Insertion times of subgenome-specific LTR-RTs. The 95% confidence interval (CI) is marked in the upper right corner and was used to predict the insertion time boundaries of the LTR-RTs on the subgenome. (**F**) Phylogenetic tree of up to 1,000 *Gypsy* LTR-RTs randomly selected from the subgenome-specific LTR-RTs. The branches are colored by subgenome and the terminal nodes are colored by clade. Clades were classified using TEsorter. (B–F) Colors are consistent for subgenomes. SG1 = subgenome C, SG2 = subgenome D.

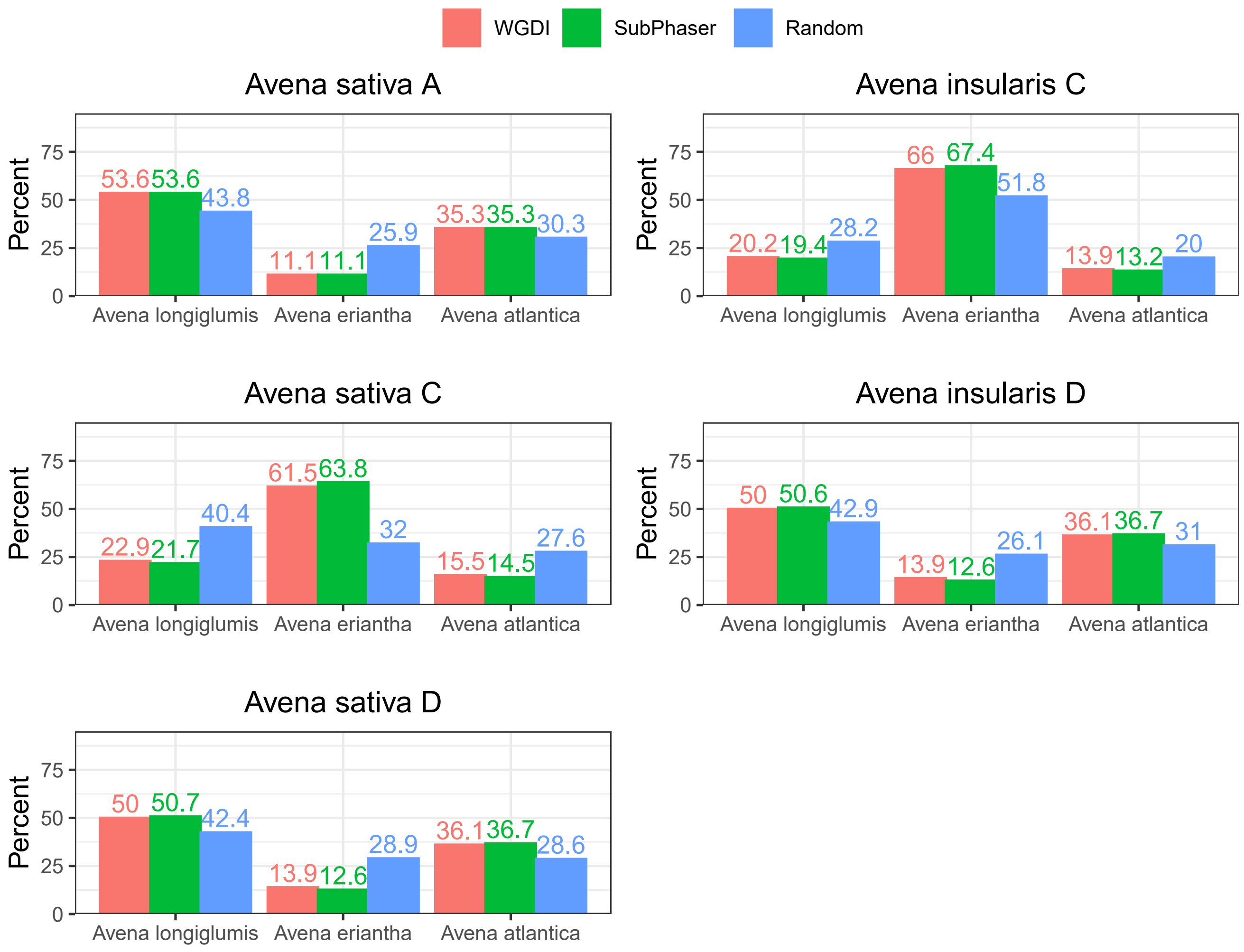

Figure S12. Comparison of similarity between polyploid subgenomes and diploid potential progenitors in the oat case. Y-axes, percent of best hits from genes of one subgenome to those of multiple diploids. Red, WGDI; green, SubPhaser; blue, random sorting.

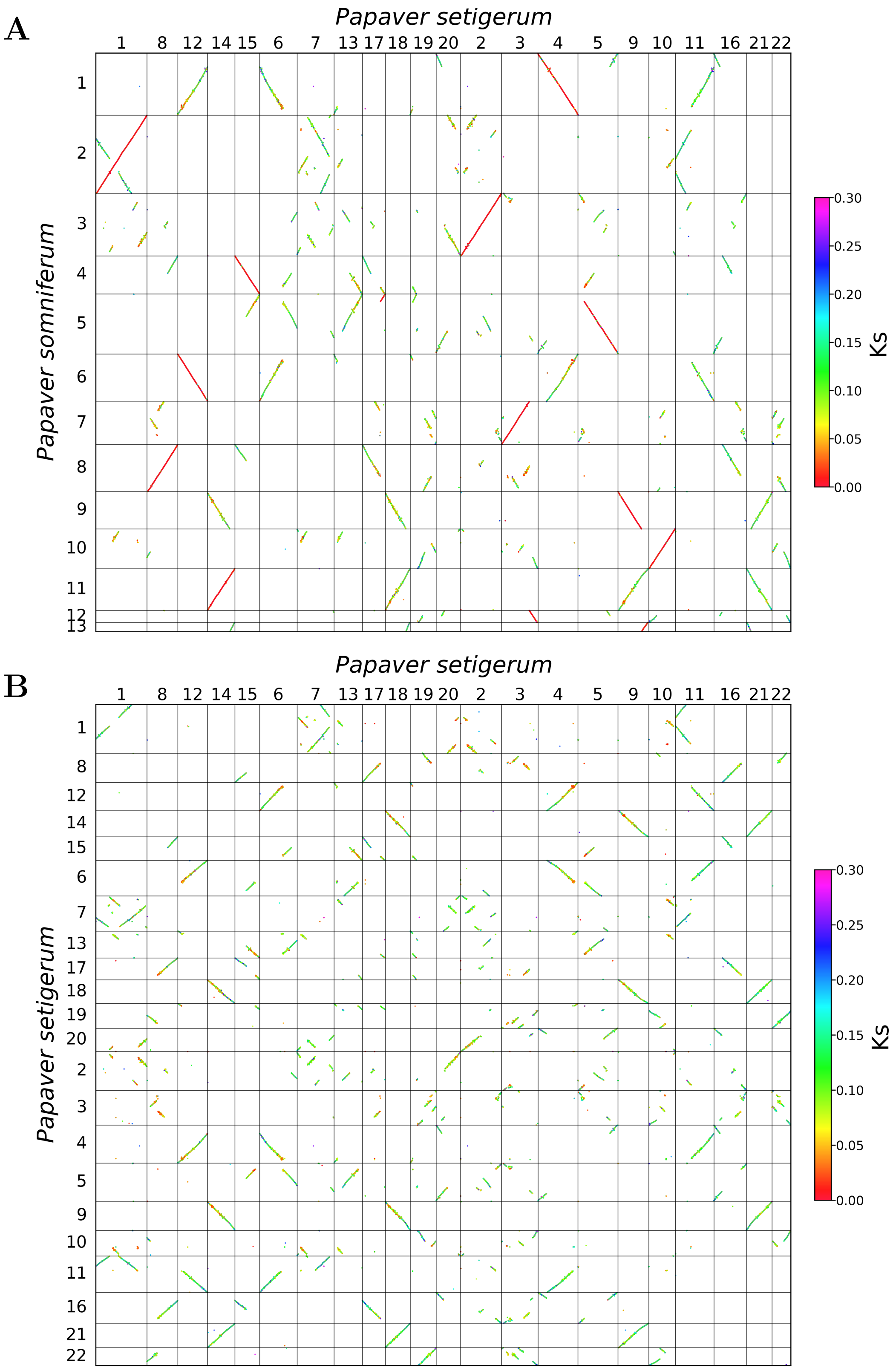

Figure S13. Dot plots colored by Ks distance for syntenic gene pairs. (**A**) Synteny between neoallooctoploid poppy (AABBCCDD, *Papaver setigerum*) and neoallotetraploid poppy (AACC, *Papaver somniferum*). (**B**) Synteny within neoallooctoploid poppy (AABBCCDD, *Papaver setigerum*).

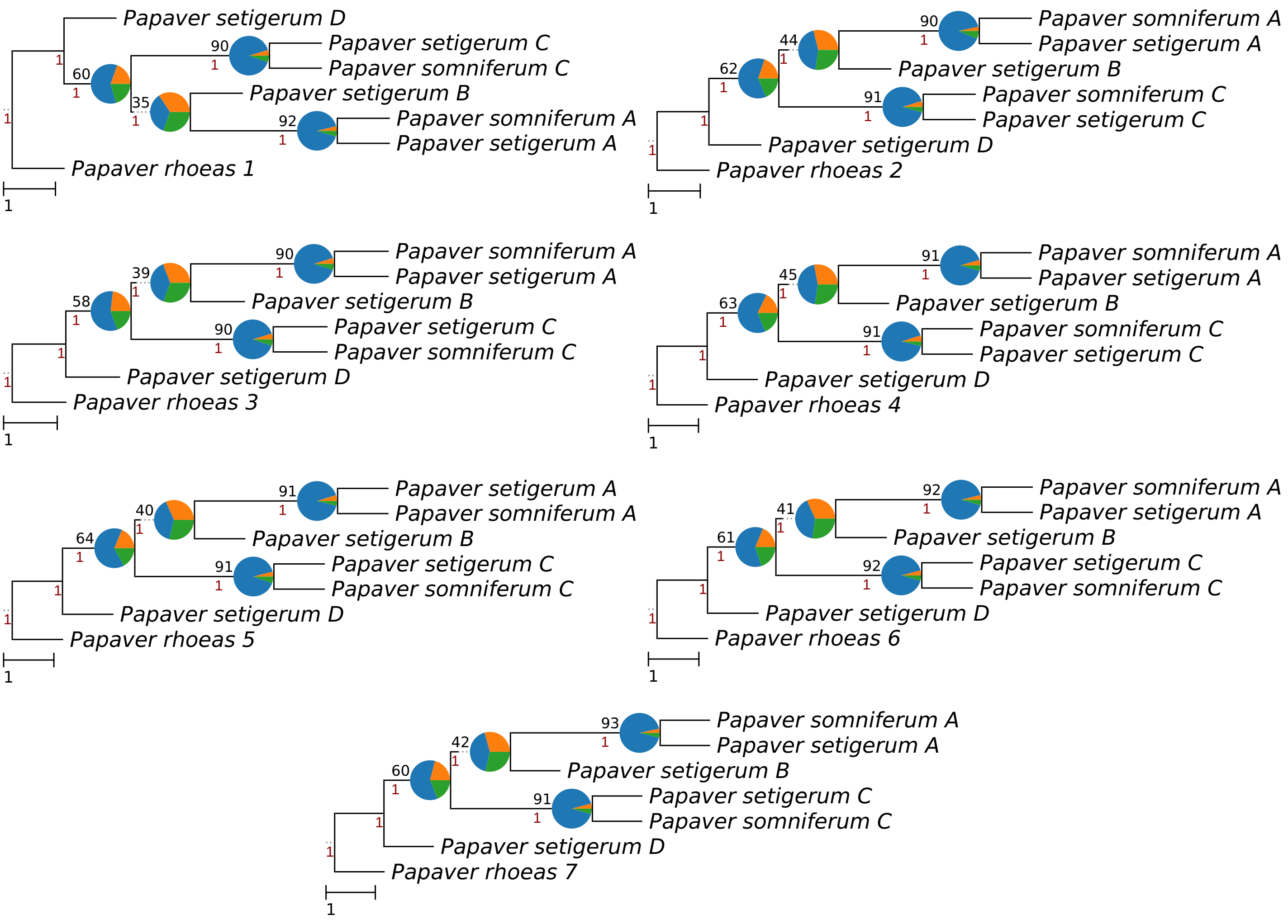

Figure S14. The phylogenetic tree constructed with each chromosome of four subgenomes of neoallooctoploid poppy (AABBCCDD, *Papaver setigerum*), and two subgenomes of neoallotetraploid poppy (AACC, *Papaver somniferum*). *Papaver rhoeas* serves as the outgroup. Numbers above the branches represent the percentages of concordance between gene trees and species/subgenome tree, and numbers below the branch represent the local posterior probabilities calculated in ASTRAL. Pie plots at the nodes represent the percentages of three gene tree topology (q1, q2 and q3) calculated in ASTRAL. Bar, 1.0 coalescent units.

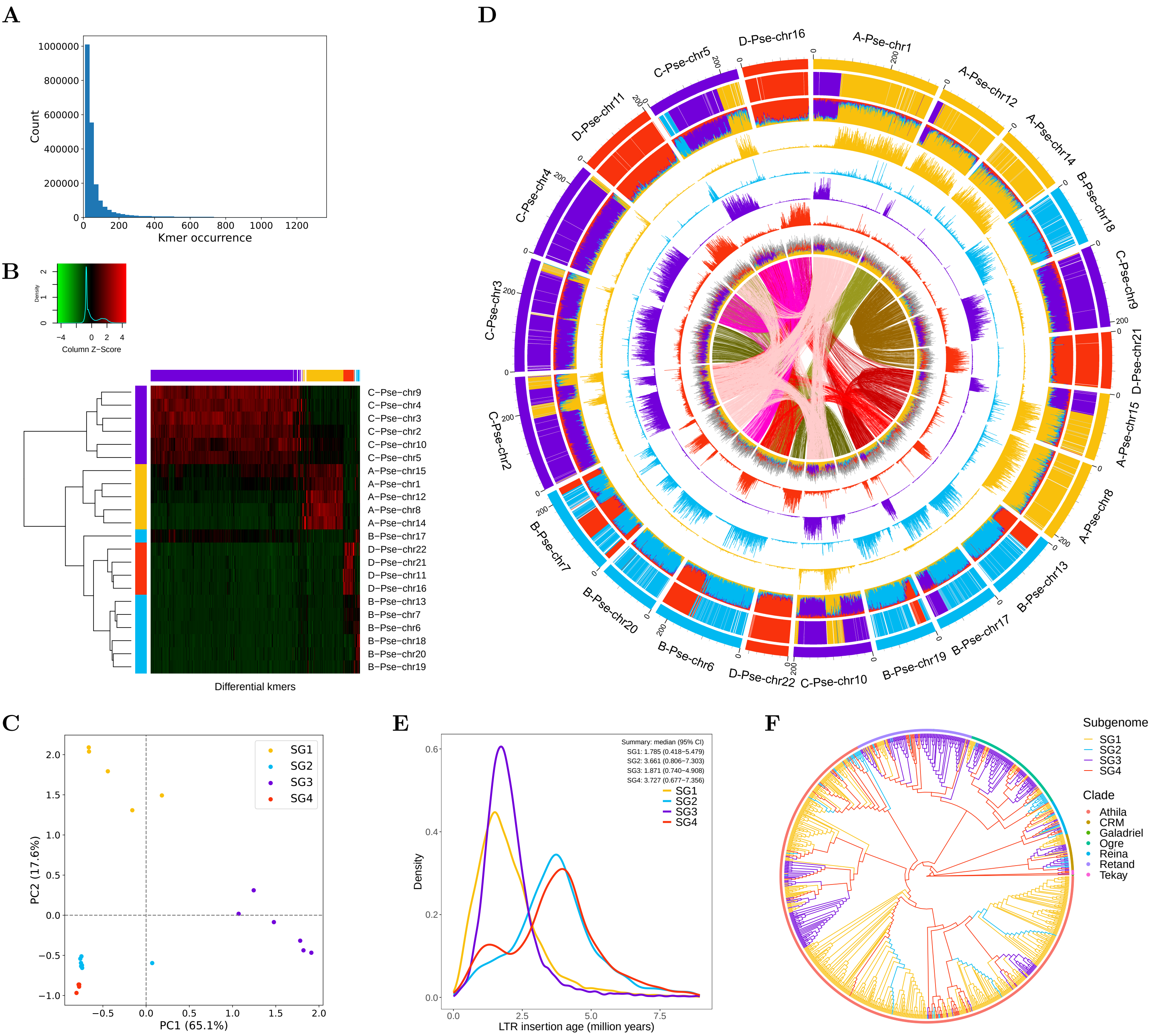

Figure S15. Subgenome phasing of *Papaver setigerum* using SubPhaser. (**A**) Frequency distribution of differential 15-mers among the homoeologous chromosomes. (**B**) Unsupervised hierarchical clustering. The horizontal color bar at the top of the axis indicates to which subgenome the k-mer is specific. The vertical color bar on the left of the axis indicates to which subgenome the chromosome is assigned. The heatmap indicates the Z-scaled relative abundance of k-mers; the larger the Z score, the higher the relative abundance of a k-mer. (**C**) Principal component analysis (PCA) of differential 15-mers. (**D**) Chromosomal characteristics. From outer to inner circles (1–9): (1) Subgenome assignments based on the k-Means algorithm. (2) Significant enrichment of subgenome-specific k-mers. Subgenome-specific k-mers with the same color as the subgenome are significantly enriched. White areas are not significantly enriched. (3) Normalized proportion (relative) of subgenome-specific k-mers. (4–7) Counts (absolute) of each subgenome-specific k-mer set. (8) Density of long terminal repeat retrotransposons (LTR-RTs). If the color is consistent with that of the subgenome, it indicates that the LTR-RTs are significantly enriched in those subgenome-specific k-mers. Gray indicates non-specific LTR-RTs. (9) Homoeologous blocks. All statistics (2–8) were computed in sliding windows of 1 Mb. (**E**) Insertion times of subgenome-specific LTR-RTs. The 95% confidence interval (CI) is marked in the upper right corner and was used to predict the insertion time boundaries of LTR-RTs on the subgenome. (**F**) Phylogenetic tree of up to 1,000 *Gypsy* LTR-RTs randomly selected from the subgenome-specific LTR-RTs. The branches are colored by subgenome and the terminal nodes are colored by clade. Clades were classified using TEsorter. (B–F) Colors are consistent for subgenomes. SG1 = subgenome A, SG2 = subgenome B, SG3 = subgenome C, SG4 = subgenome D. The results were adopted from Zhang et al (2023).

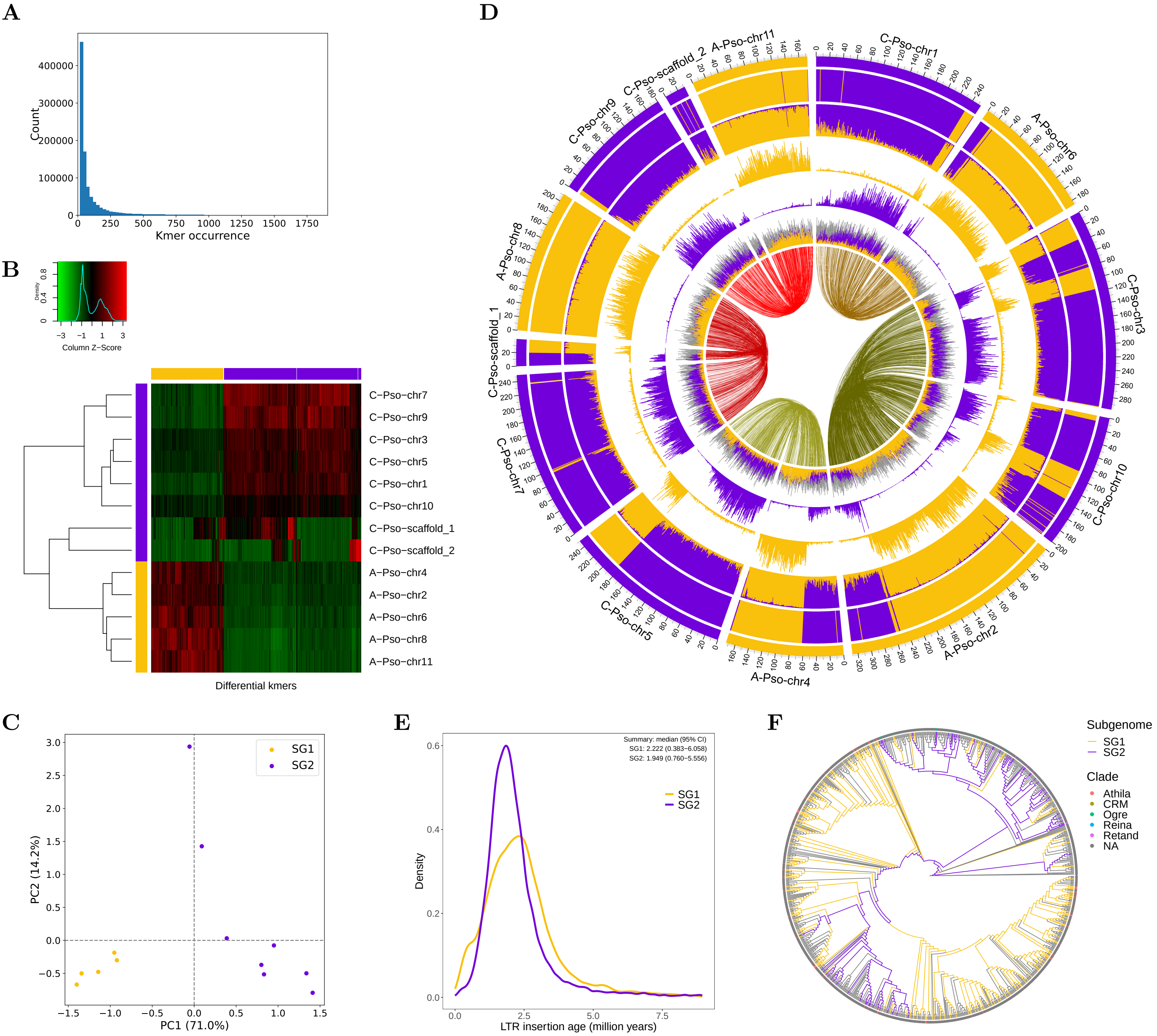

Figure S16. Subgenome phasing of *Papaver somniferum* using SubPhaser. (**A**) Frequency distribution of differential 15-mers among the homoeologous chromosomes. (**B**) Unsupervised hierarchical clustering. The horizontal color bar at the top of the axis indicates to which subgenome the k-mer is specific. The vertical color bar on the left of the axis indicates to which subgenome the chromosome is assigned. The heatmap indicates the Z-scaled relative abundance of k-mers; the larger the Z score, the higher the relative abundance of a k-mer. (**C**) Principal component analysis (PCA) of differential 15-mers. (**D**) Chromosomal characteristics. From outer to inner circles (1–7): (1) Subgenome assignments based on the k-Means algorithm. (2) Significant enrichment of subgenome-specific k-mers. Subgenome-specific k-mers with the same color as the subgenome are significantly enriched. White areas are not significantly enriched. (3) Normalized proportion (relative) of subgenome-specific k-mers. (4–5) Counts (absolute) of each subgenome-specific k-mer set. (6) Density of long terminal repeat retrotransposons (LTR-RTs). If the color is consistent with that of the subgenome, it indicates that the LTR-RTs are significantly enriched in those subgenome-specific k-mers. Gray indicates non-specific LTR-RTs. (7) Homoeologous blocks. All statistics (2–6) were computed in sliding windows of 1 Mb. (**E**) Insertion times of subgenome-specific LTR-RTs. The 95% confidence interval (CI) is marked in the upper right corner and was used to predict the insertion time boundaries of LTR-RTs on the subgenome. (**F**) Phylogenetic tree of up to 1,000 *Gypsy* LTR-RTs randomly selected from the subgenome-specific LTR-RTs. The branches are colored by subgenome and the terminal nodes are colored by clade. Clades were classified using TEsorter. (B–F) Colors are consistent for subgenomes. SG1 = subgenome A, SG2 = subgenome C. The results were adopted from Zhang et al (2023).

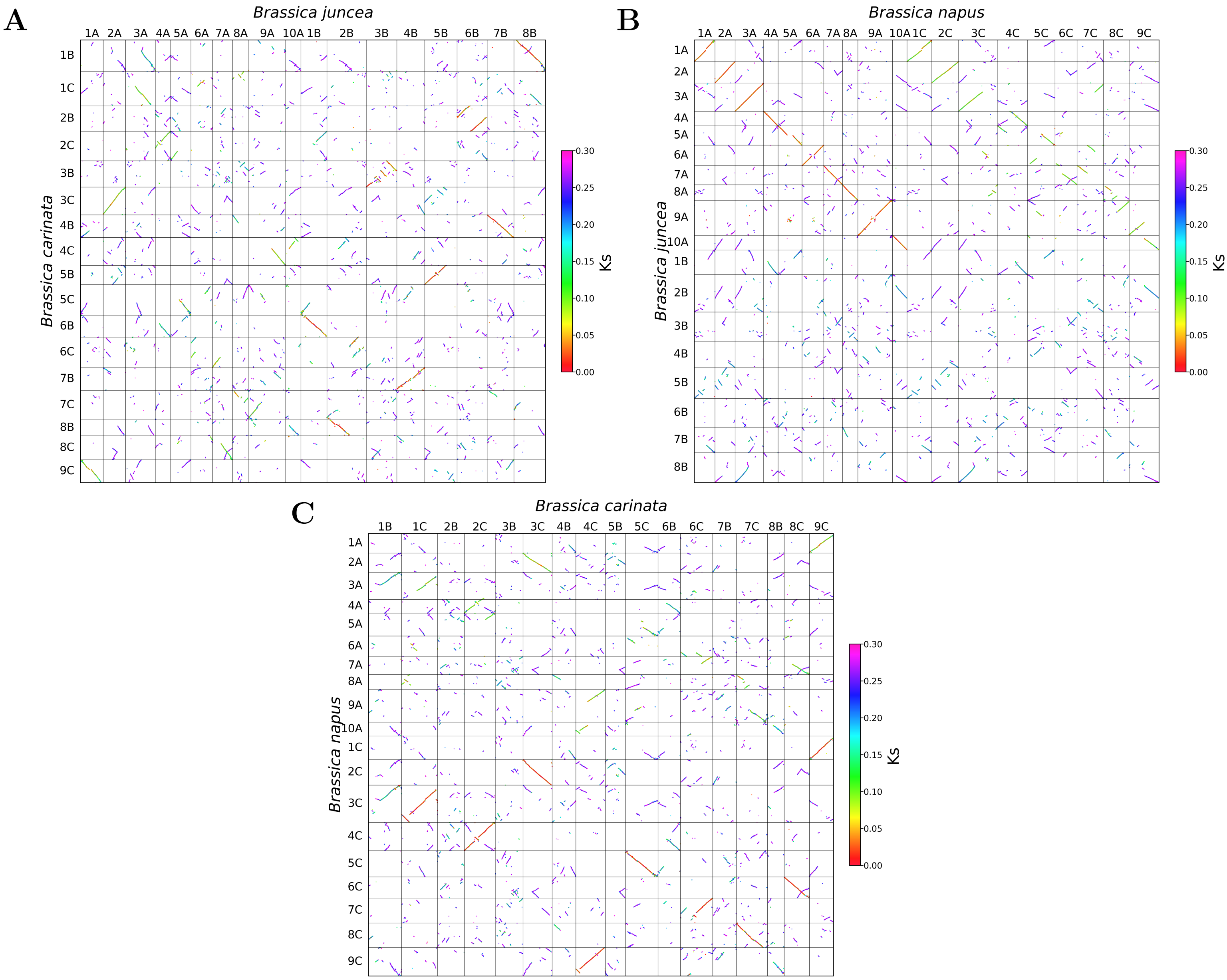

Figure S17. Dot plots colored with Ks distance for syntenic gene pairs. (**A**) Synteny between the allotetraploid *Brassica juncea* (AABB, 2n = 4x = 36) and the allotetraploid *Brassica carinata* (BBCC, 2n=4x=34). (**B**) Synteny between the allotetraploid *Brassica juncea* (AABB, 2n = 4x = 36) and the allotetraploid *Brassica napus* (AACC, 2n=4x=38). (**C**) Synteny between the allotetraploid *Brassica napus* (AACC, 2n = 4x = 38) and the allotetraploid *Brassica carinata* (BBCC, 2n = 4x = 34).

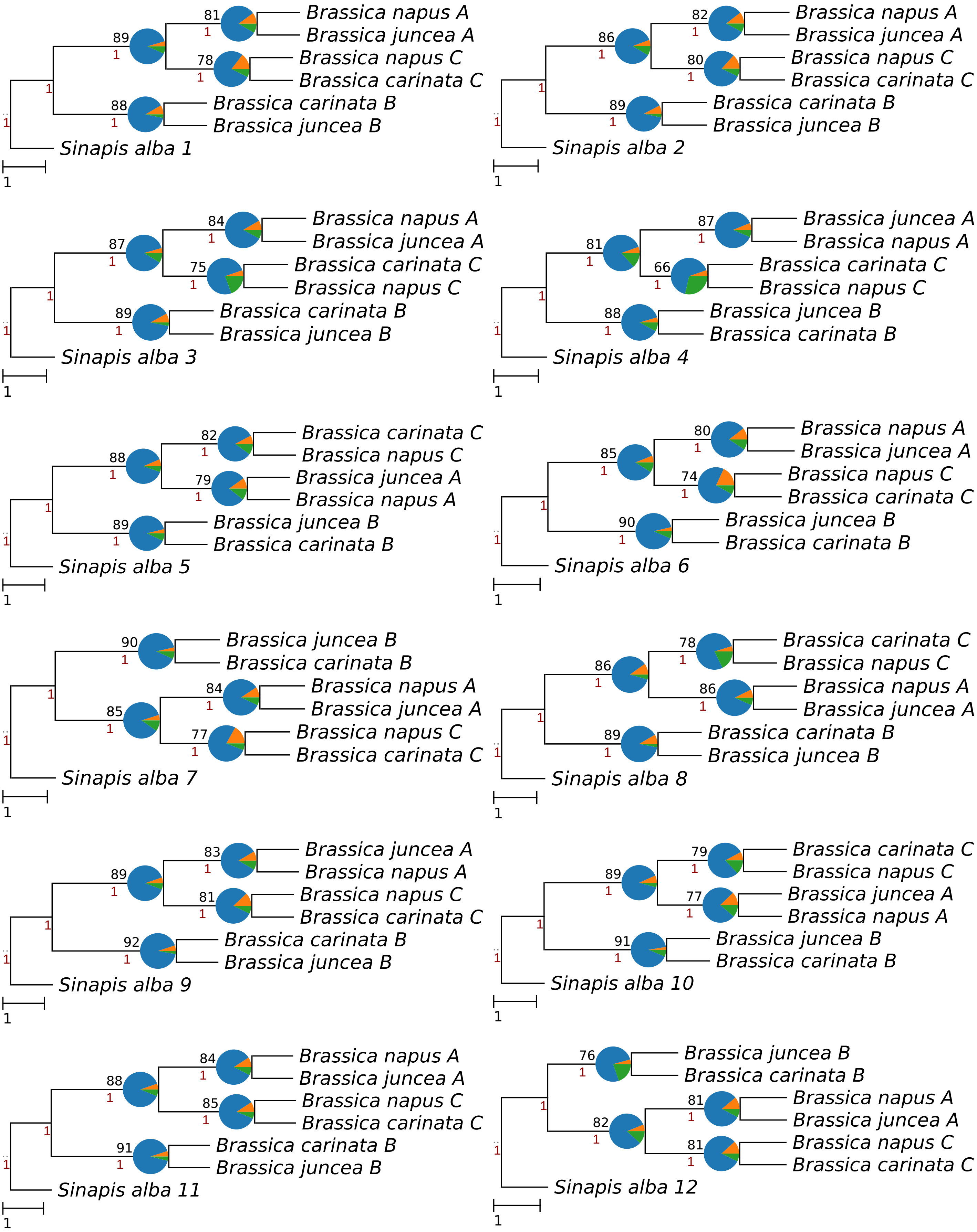

Figure S18. The phylogenetic tree constructed with each chromosome of the allotetraploid *Brassica juncea* (AABB, 2n = 4x = 36), the allotetraploid Brassica carinata (BBCC, 2n = 4x = 34) and allotetraploid *Brassica napus* (AACC, 2n = 4x = 38). *Sinapis alba* served as the outgroup. Numbers above the branches represent the percentages of concordance between gene and species/subgenome trees, and numbers below the branches represent the local posterior probabilities calculated in ASTRAL. Pie plots at the nodes represent the percentages of three gene tree topologies (q1, q2 and q3) calculated in ASTRAL. Bar, 1.0 coalescent units.

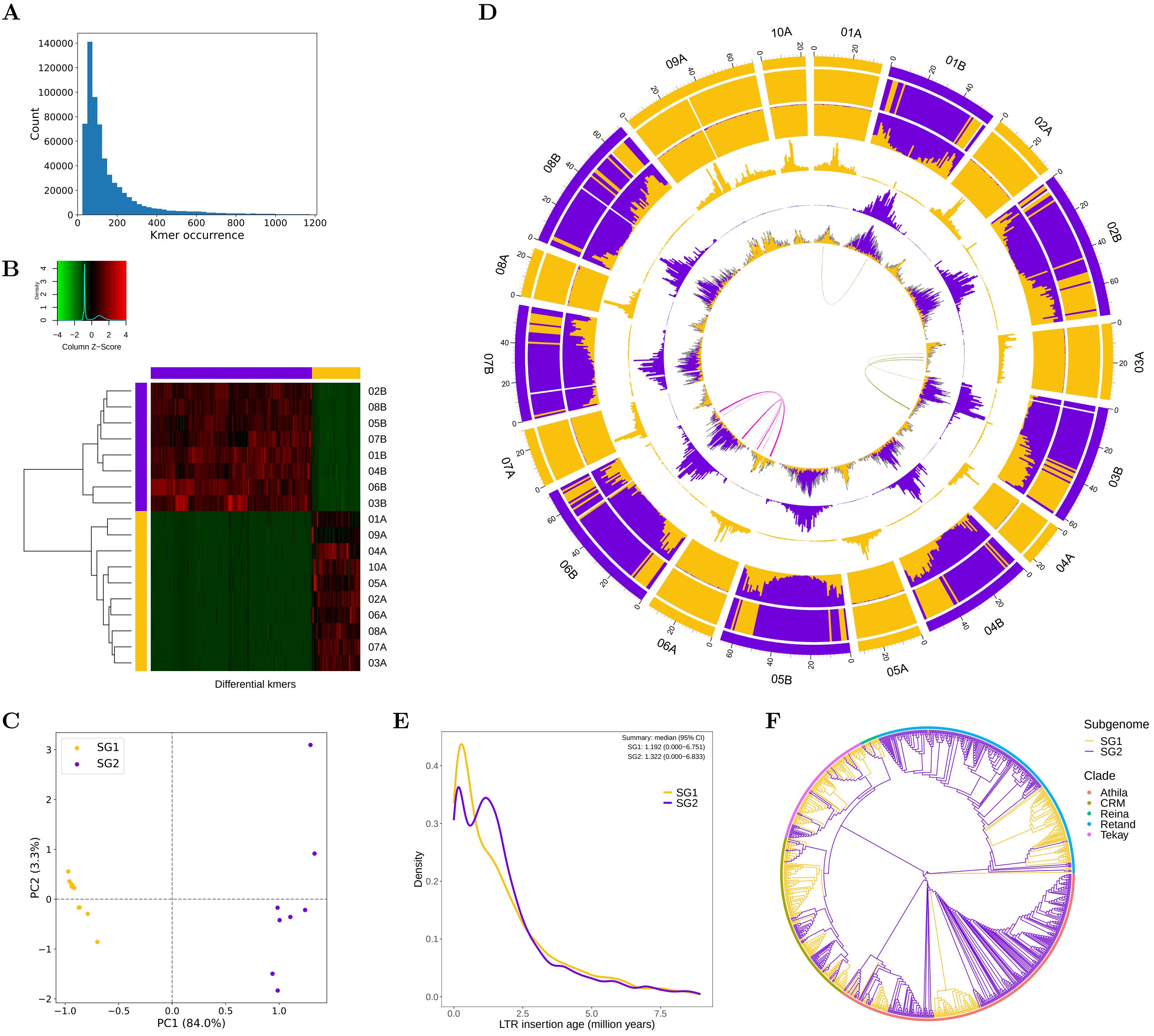

Figure S19. Subgenome phasing of *Brassica juncea* using SubPhaser. (**A**) Frequency distribution of differential 15-mers among the homoeologous chromosomes. (**B**) Unsupervised hierarchical clustering. The horizontal color bar at the top of the axis indicates to which subgenome the *k*-mer is specific. The vertical color bar on the left of the axis indicates to which subgenome the chromosome is assigned. The heatmap indicates the *Z*-scaled relative abundance of *k*-mers; the larger the *Z* score, the higher the relative abundance of a *k*-mer. (**C**) Principal component analysis (PCA) of differential 15-mers. (**D**) Chromosomal characteristics. From outer to inner circles (1–7): (1) Subgenome assignments based on the *k*-Means algorithm. (2) Significant enrichment of subgenome-specific *k*-mers. Subgenome-specific k-mers with the same color as the subgenome significantly enriched. White areas are not significantly enriched. (3) Normalized proportion (relative) of subgenome-specific *k*-mers. (4–5) Counts (absolute) of each subgenome-specific *k*-mer set. (6) Density of long terminal repeat retrotransposons (LTR-RTs). If the color is consistent with that of the subgenome, it indicates that LTR-RTs are significantly enriched in those subgenome-specific *k*-mers. Gray indicates non-specific LTR-RTs. (7) Homoeologous blocks. All statistics (2–6) were computed in sliding windows of 1 Mb. (**E**) Insertion times of subgenome-specific LTR-RTs. The 95% confidence interval (CI) is marked in the upper right corner and was used to predict the insertion time boundaries of LTR-RTs on the subgenome. (**F**) Phylogenetic tree of up to 1,000 *Gypsy* LTR-RTs randomly selected from the subgenome-specific LTR-RTs. The branches are colored by subgenome and the terminal nodes are colored by clade. Clades were classified using TEsorter. (B–F) Colors are consistent for subgenomes. SG1 = subgenome A, SG2 = subgenome B.

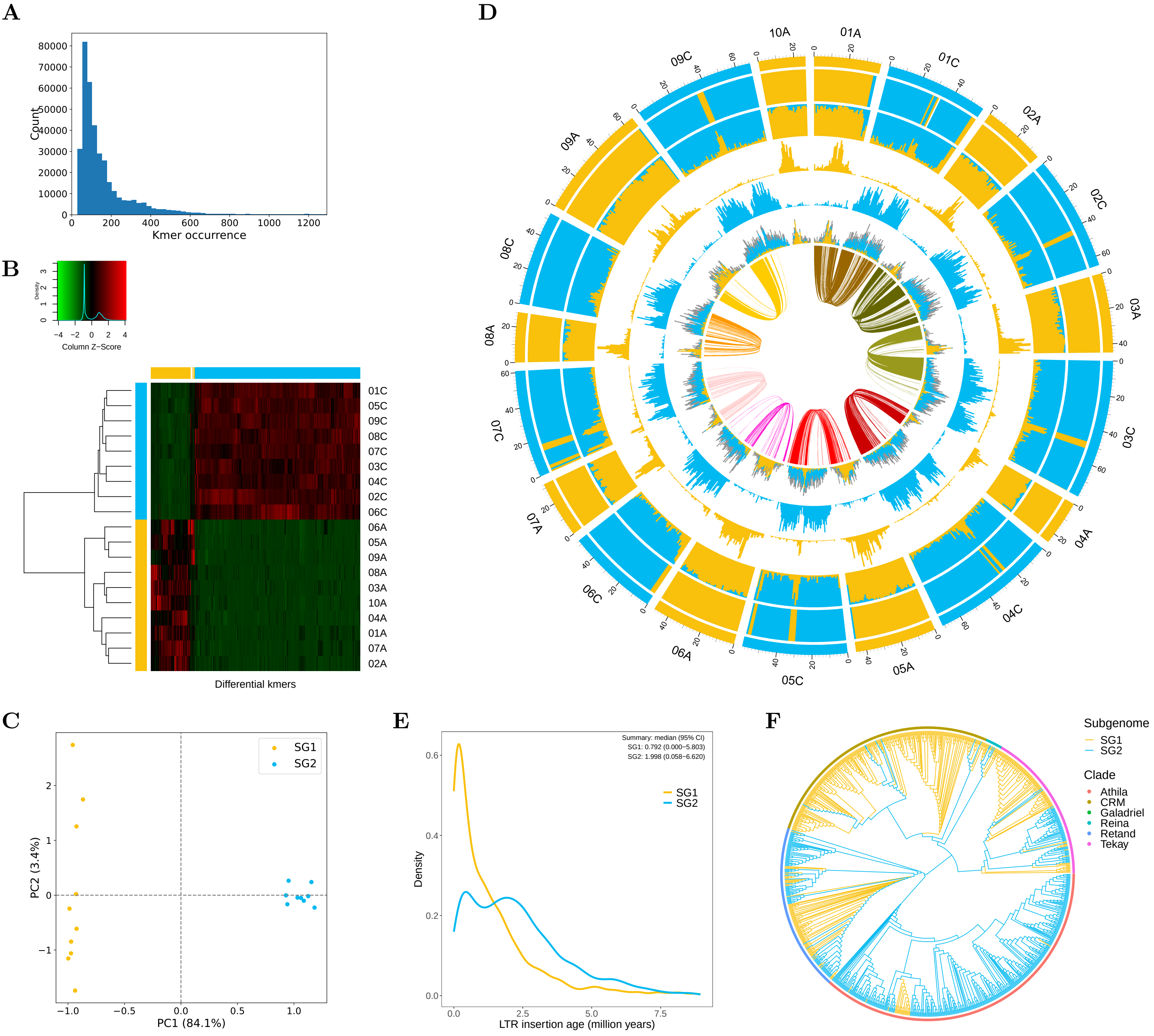

Figure S20. Subgenome phasing of *Brassica napus* using SubPhaser. (**A**) Frequency distribution of differential 15-mers among the homoeologous chromosomes. (**B**) Unsupervised hierarchical clustering. The horizontal color bar at the top of the axis indicates to which subgenome the *k*-mer is specific. The vertical color bar on the left of the axis indicates to which subgenome the chromosome is assigned. The heatmap indicates the *Z*-scaled relative abundance of *k*-mers; the larger the *Z* score, the higher the relative abundance of a *k*-mer. (**C**) Principal component analysis (PCA) of differential 15-mers. (**D**) Chromosomal characteristics. From outer to inner circles (1–7): (1) Subgenome assignments based on the *k*-Means algorithm. (2) Significant enrichment of subgenome-specific *k*-mers. Subgenome-specific k-mers with the same color as the subgenome are significantly enriched. White areas are not significantly enriched. (3) Normalized proportion (relative) of subgenome-specific *k*-mers. (4–5) Counts (absolute) of each subgenome-specific *k*-mer set. (6) Density of long terminal repeat retrotransposons (LTR-RTs). If the color is consistent with that of the subgenome, it indicates that the LTR-RTs are significantly enriched in those subgenome-specific *k*-mers. Gray indicates non-specific LTR-RTs. (7) Homoeologous blocks. All statistics (2–6) were computed in sliding windows of 1 Mb. (**E**) Insertion times of subgenome-specific LTR-RTs. The 95% confidence interval (CI) is marked in the upper right corner and was used to predict the insertion time boundaries of LTR-RTs on the subgenome. (**F**) Phylogenetic tree of up to 1,000 *Gypsy* LTR-RTs randomly selected from the subgenome-specific LTR-RTs. The branches are colored by subgenome and the terminal nodes are colored by clade. Clades were classified using TEsorter. (B–F) Colors are consistent for subgenomes. SG1 = subgenome A, SG2 = subgenome C.

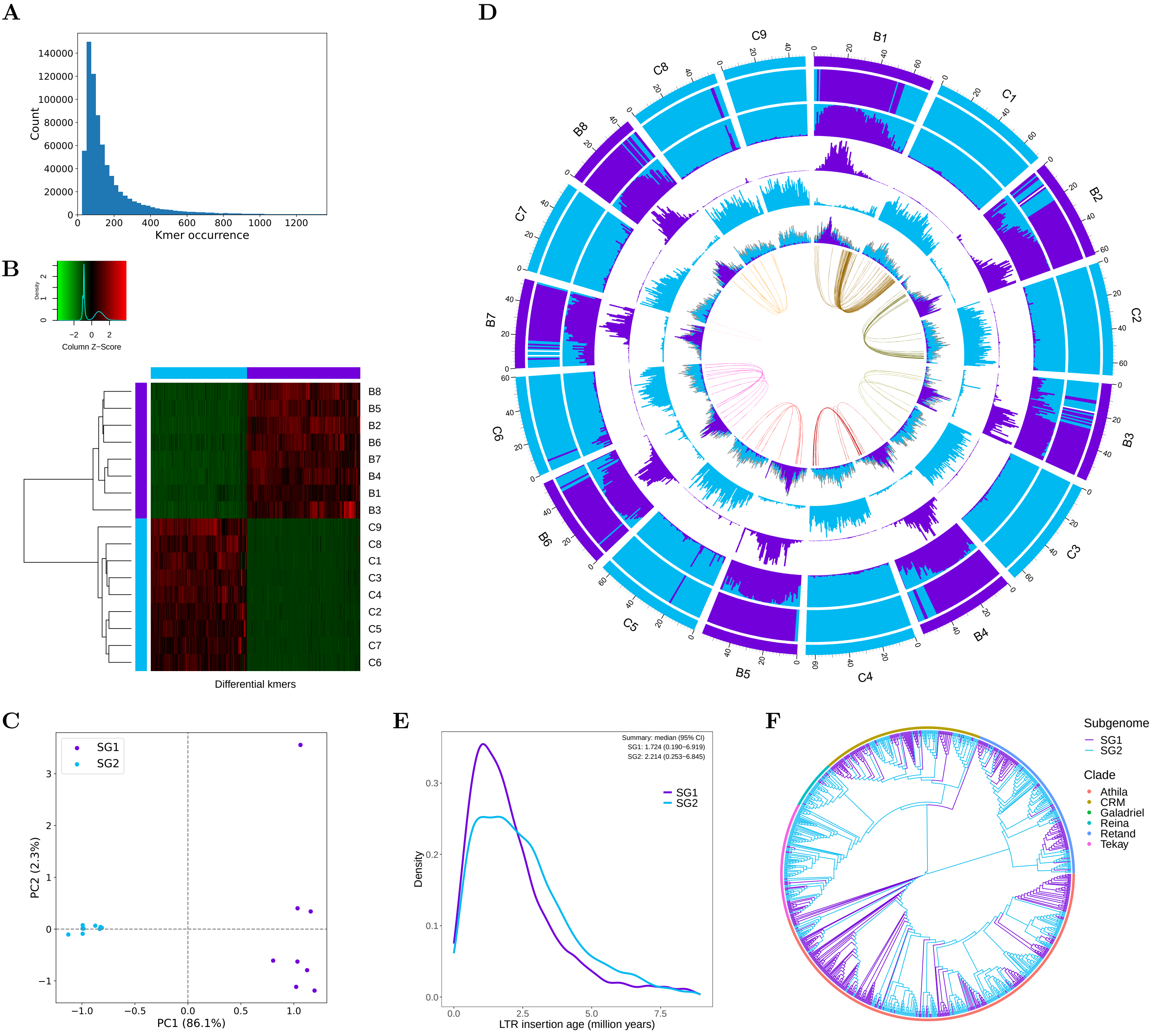

Figure S21. Subgenome phasing of *Brassica carinata* using SubPhaser. (**A**) Frequency distribution of differential 15-mers among the homoeologous chromosomes. (**B**) Unsupervised hierarchical clustering. The horizontal color bar at the top of the axis indicates to which subgenome the k-mer is specific. The vertical color bar on the left of the axis indicates to which subgenome the chromosome is assigned. The heatmap indicates the Z-scaled relative abundance of k-mers; the larger the Z score, the higher the relative abundance of a k-mer. (**C**) Principal component analysis (PCA) of differential 15-mers. (**D**) Chromosomal characteristics. From outer to inner circles (1–7): (1) Subgenome assignments based on the k-Means algorithm. (2) Significant enrichment of subgenome-specific k-mers. Subgenome-specific k-mers with the same color as the subgenome are significantly enriched. White areas are not significantly enriched. (3) Normalized proportion (relative) of subgenome-specific k-mers. (4–5) Counts (absolute) of each subgenome-specific k-mer set. (6) Density of long terminal repeat retrotransposons (LTR-RTs). If the color is consistent with that of the subgenome, it indicates that the LTR-RTs are significantly enriched in those subgenome-specific k-mers. Gray indicates non-specific LTR-RTs. (7) Homoeologous blocks. All statistics (2–6) were computed in sliding windows of 1 Mb. (**E**) Insertion times of subgenome-specific LTR-RTs. The 95% confidence interval (CI) is marked in the upper right corner and was used to predict the insertion time boundaries of LTR-RTs on the subgenome. (**F**) Phylogenetic tree of up to 1,000 *Gypsy* LTR-RTs randomly selected from the subgenome-specific LTR-RTs. The branches are colored by subgenome and the terminal nodes are colored by clade. Clades were classified using TEsorter. (B–F) Colors are consistent for subgenomes. SG1 = subgenome B, SG2 = subgenome C. The results were adopted from Jia et al (2022).

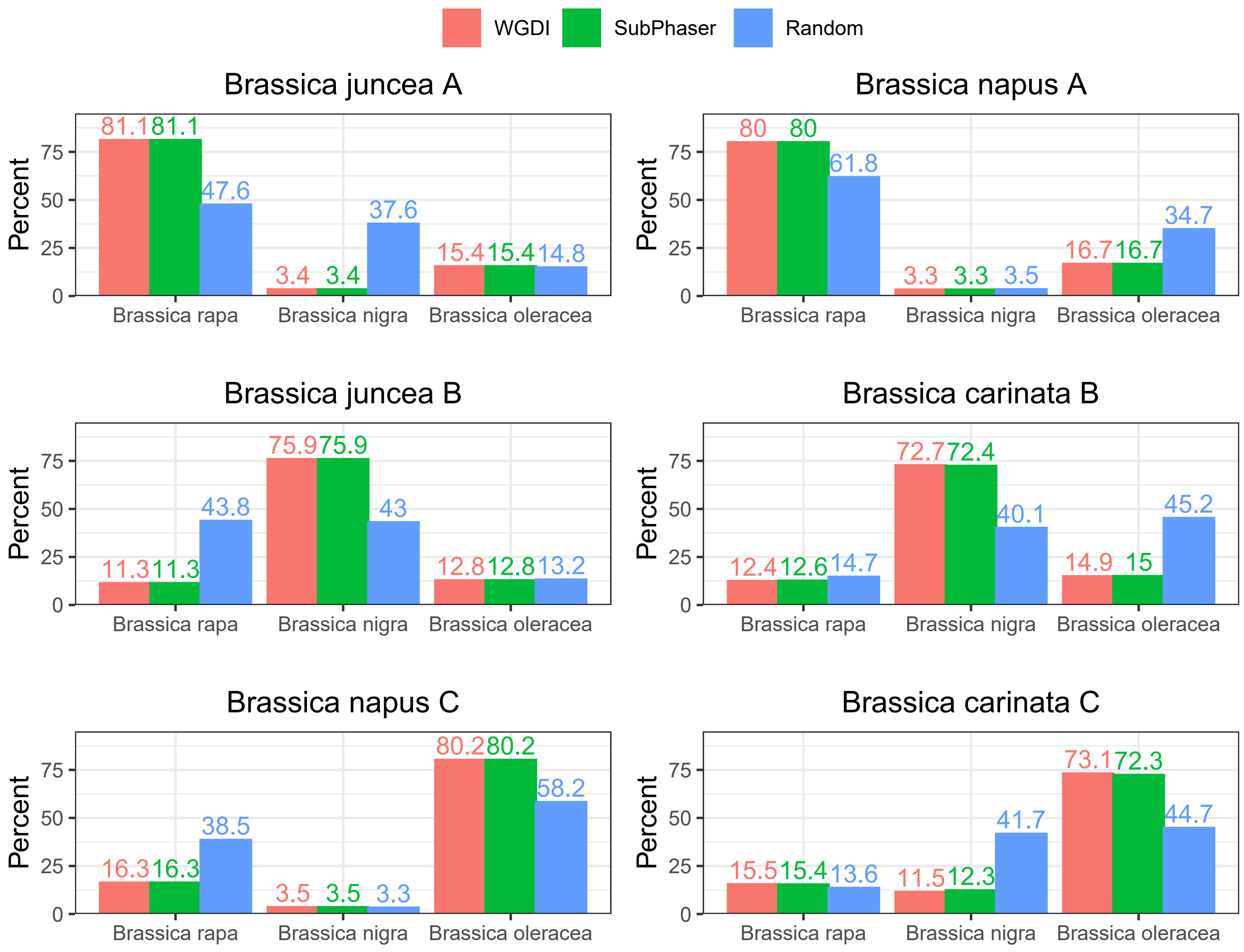

Figure S22. Comparison of similarity between polyploid subgenomes and diploid potential progenitors in the *Brassica* case. Y-axes, percent of best hits from genes of one subgenome to those of multiple diploids. Red, WGDI; green, SubPhaser; blue, random sorting.

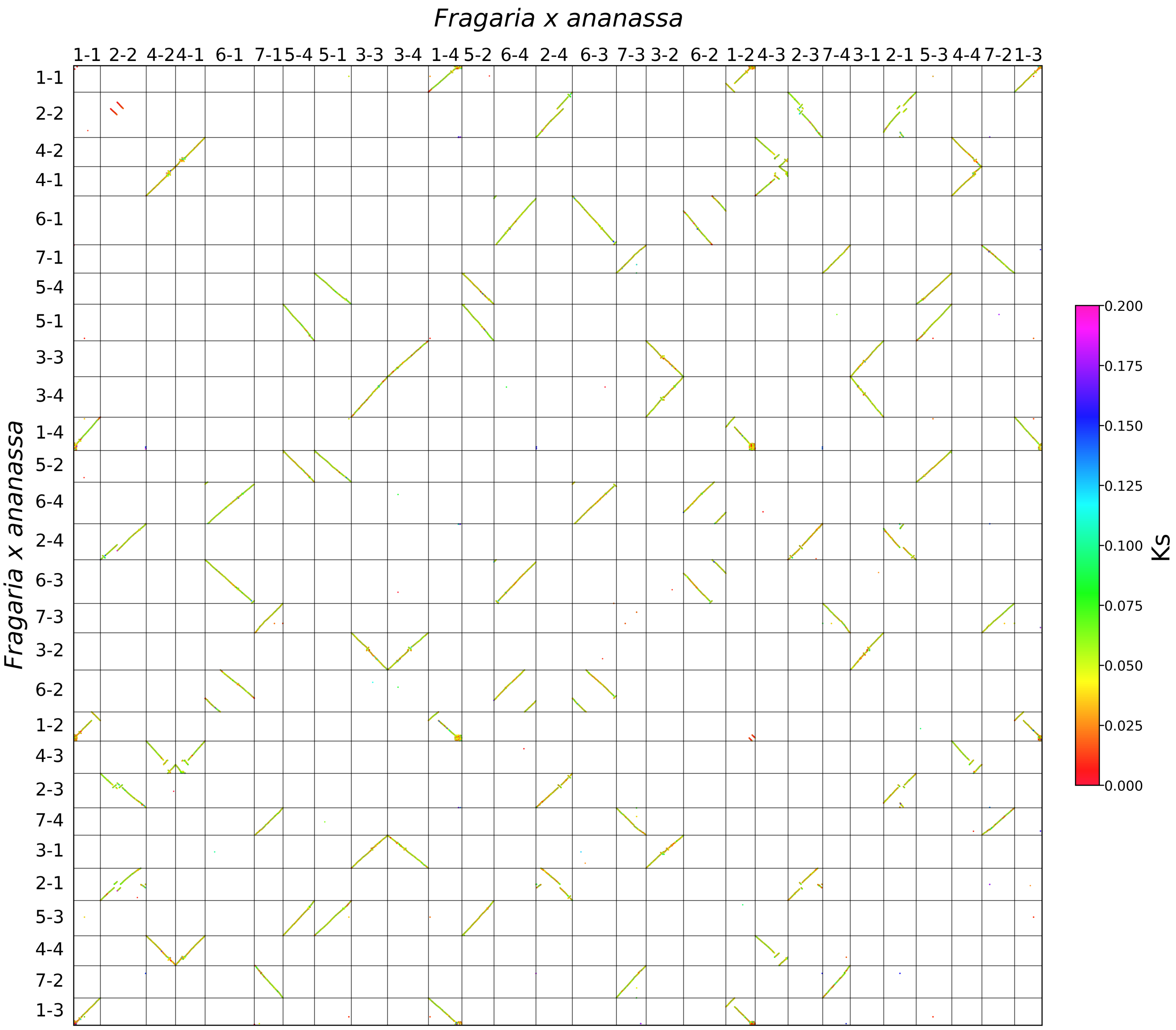

Figure S23. Dot plot colored with Ks distance for syntenic gene pairs within allooctoploid cultivated strawberry (*Fragaria* × *ananassa*).

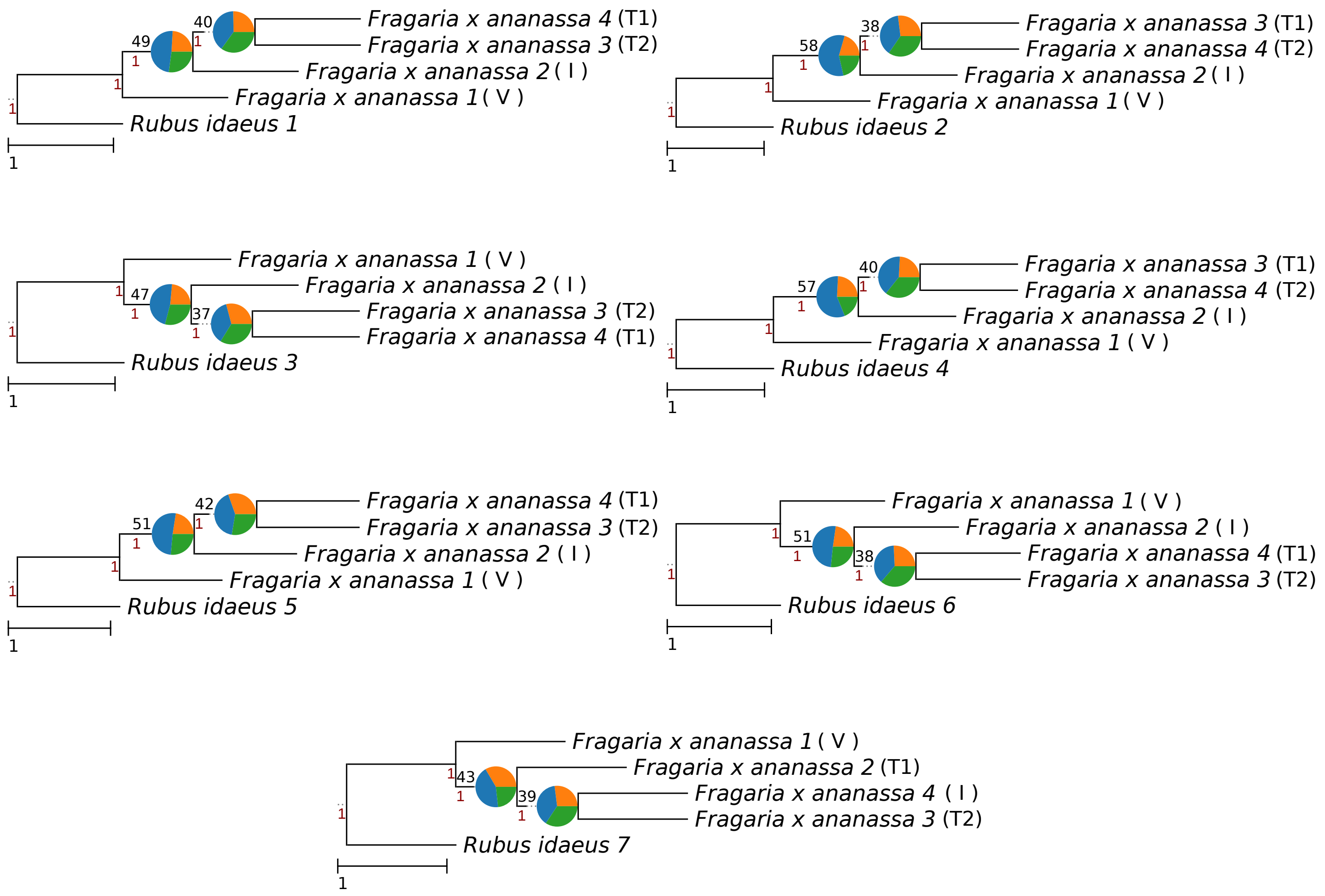

Figure S24. The phylogenetic tree constructed with each chromosome of four subgenomes of allooctoploid cultivated strawberry (*Fragaria* × *ananassa*)).*Rubus idaeus* served as the outgroup. Numbers above the branches represent the percentages of concordance between gene and species/subgenome trees, and numbers below the branches represent the local posterior probabilities calculated in ASTRAL. Pie plots at the nodes represent the percentages of three gene tree topologies (q1, q2 and q3) calculated in ASTRAL. Bar, 1.0 coalescent units. V, I, T1 and T2 was labeled according to subgenome assignments of Session et al. (2023).

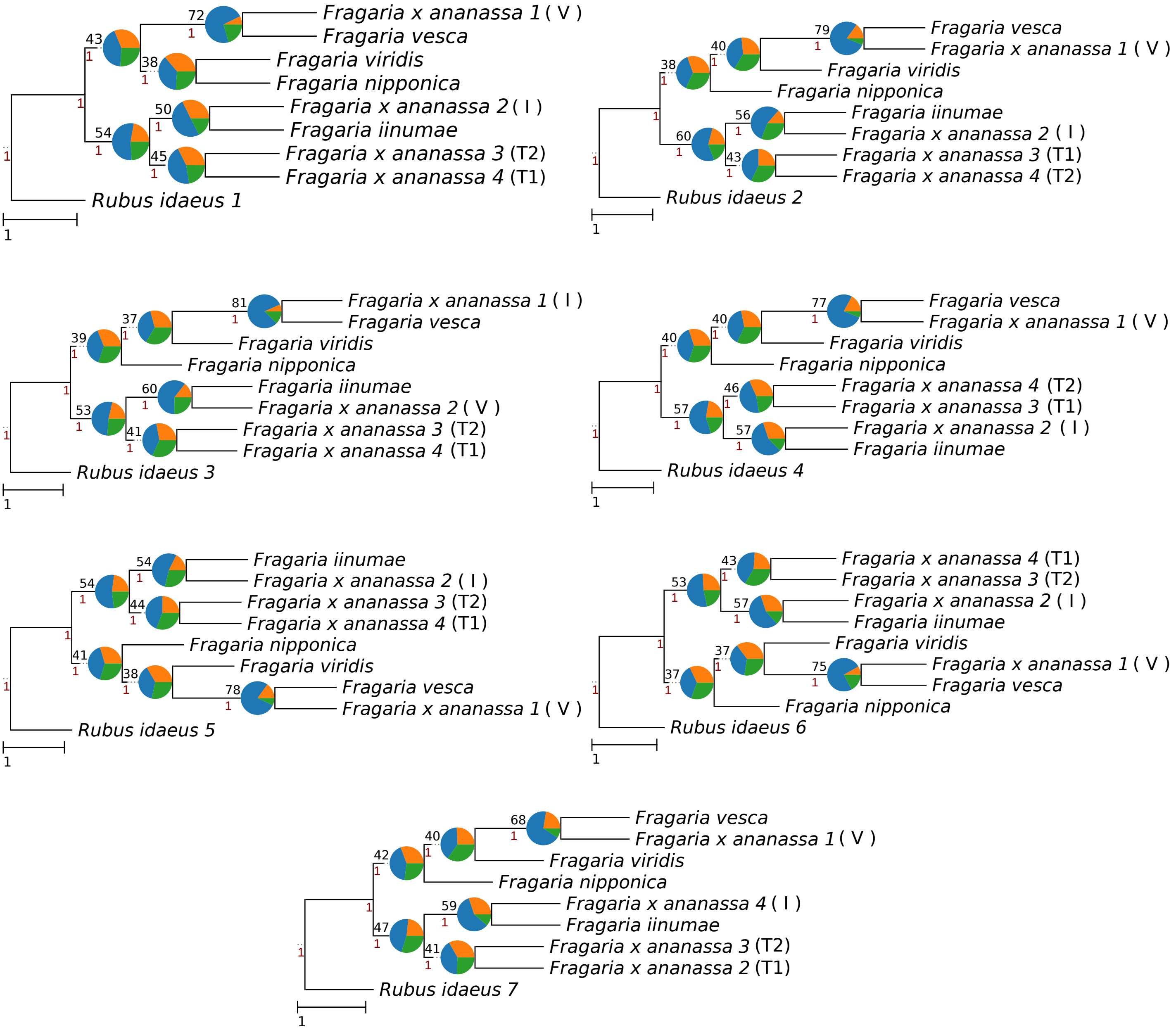

Figure S25. The phylogenetic tree constructed with each chromosome of four subgenomes of allooctoploid cultivated strawberry (*Fragaria* × *ananassa*)), *Fragaria iinumae*, *Fragaria nipponica*, *Fragaria viridis*, and *Fragaria vesca*. *Rubus idaeus* served as the outgroup. Numbers above the branches represent the percentages of concordance between gene trees and species/subgenome tree, and numbers below the branches represent the local posterior probabilities calculated in ASTRAL. Pie plots at the nodes represent the percentages of three gene tree topologies (q1, q2 and q3) calculated in ASTRAL. Bar, 1.0 coalescent units. V, I, T1 and T2 was labeled according to subgenome assignments of Session et al. (2023).

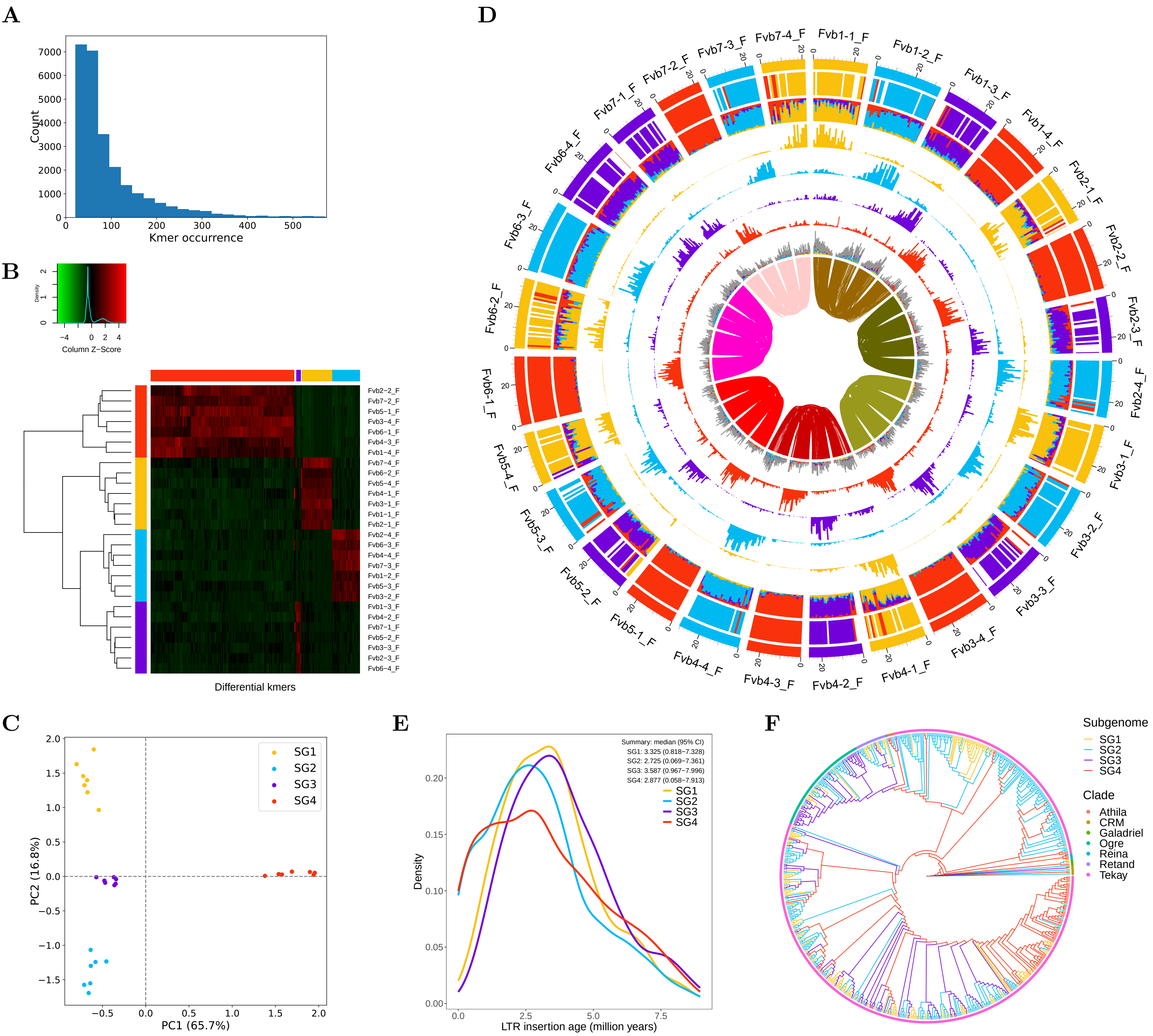

Figure S26. Subgenome phasing of *Fragaria* × *ananassa* using SubPhaser. (**A**) Frequency distribution of differential 15-mers among the homoeologous chromosomes. (**B**) Unsupervised hierarchical clustering. The horizontal color bar at the top of the axis indicates to which subgenome the k-mer is specific. The vertical color bar on the left of the axis indicates to which subgenome the chromosome is assigned. The heatmap indicates the Z-scaled relative abundance of k-mers; the larger the Z score, the higher the relative abundance of a k-mer. (**C**) Principal component analysis (PCA) of differential 15-mers. (**D**) Chromosomal characteristics. From outer to inner circles (1–9): (1) Subgenome assignments based on the k-Means algorithm. (2) Significant enrichment of subgenome-specific k-mers. Subgenome-specific k-mers with the same color as the subgenome are significantly enriched for those subgenome-specific k-mers. White areas are not significantly enriched. (3) Normalized proportion (relative) of subgenome-specific k-mers. (4–7) Counts (absolute) of each subgenome-specific k-mer set. (8) Density of long terminal repeat retrotransposons (LTR-RTs). If the color is consistent with that of the subgenome, it indicates that the LTR-RTs are significantly enriched in those subgenome-specific k-mers. Gray indicates non-specific LTR-RTs. (9) Homoeologous blocks. All statistics (2–8) were computed in sliding windows of 1 Mb. (**E**) Insertion times of subgenome-specific LTR-RTs. The 95% confidence interval (CI) is marked in the upper right corner and was used to predict the insertion time boundaries of LTR-RTs on the subgenome. (**F**) Phylogenetic tree of up to 1,000 *Gypsy* LTR-RTs randomly selected from the subgenome-specific LTR-RTs. The branches are colored by subgenome and the terminal nodes are colored by clade. Clades were classified using TEsorter. (B–F) Colors are consistent for subgenomes. SG1 = subgenome 4 (T1), SG2 = subgenome 2 (I), SG3 = subgenome 3 (T2), SG4 = subgenome 1 (V).

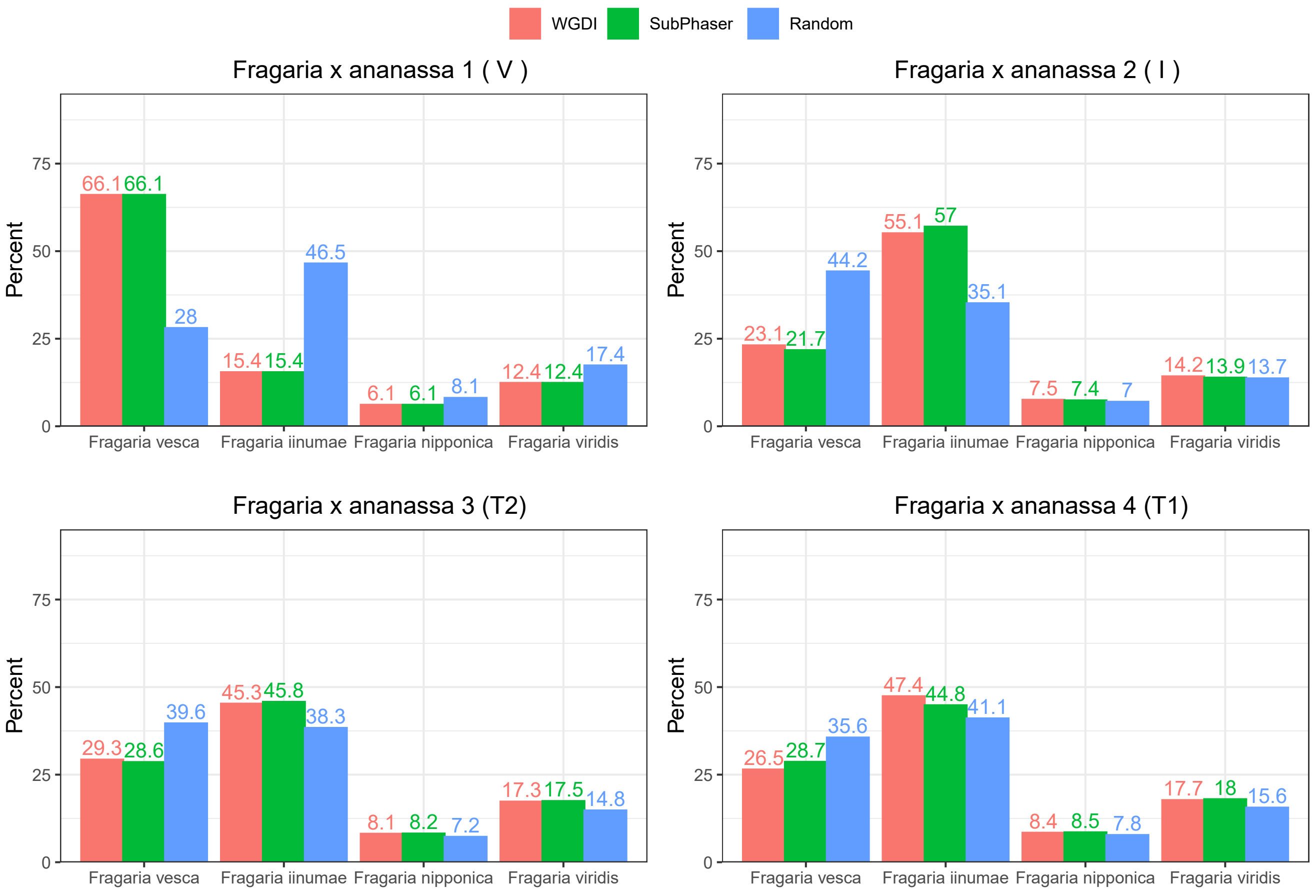

Figure S27. Comparison of similarity between polyploid subgenomes and diploid potential progenitors in the strawberry case. Y-axes, percent of best hits from genes of one subgenome to those of multiple diploids. Red, WGDI; green, SubPhaser; blue, random sorting.

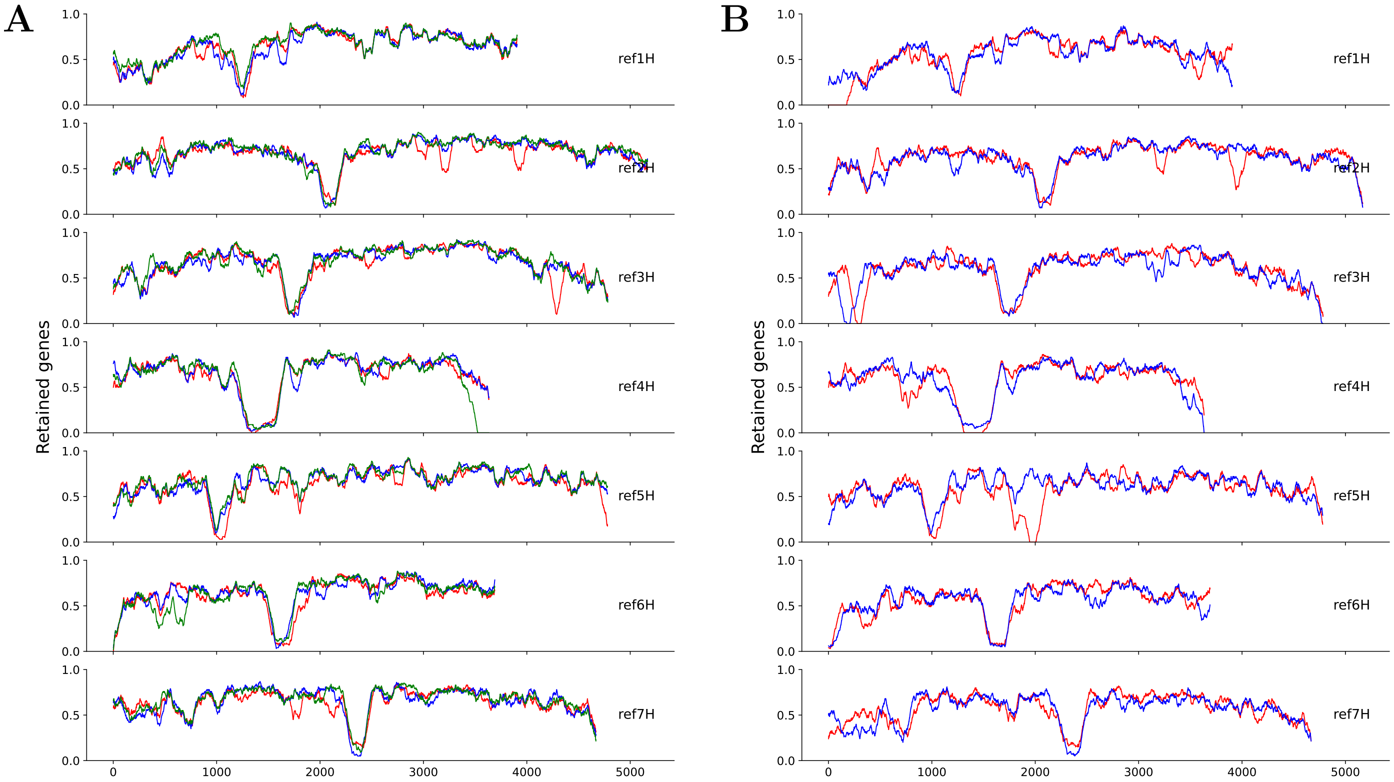

Figure S28. Gene retention of subgenomes of *Triticum aestivum* (A) and *Triticum turgidum* (B) with *Hordeum vulgare* as a reference. Red represents subgenome A, blue represents subgenome B, and green represents subgenome D.

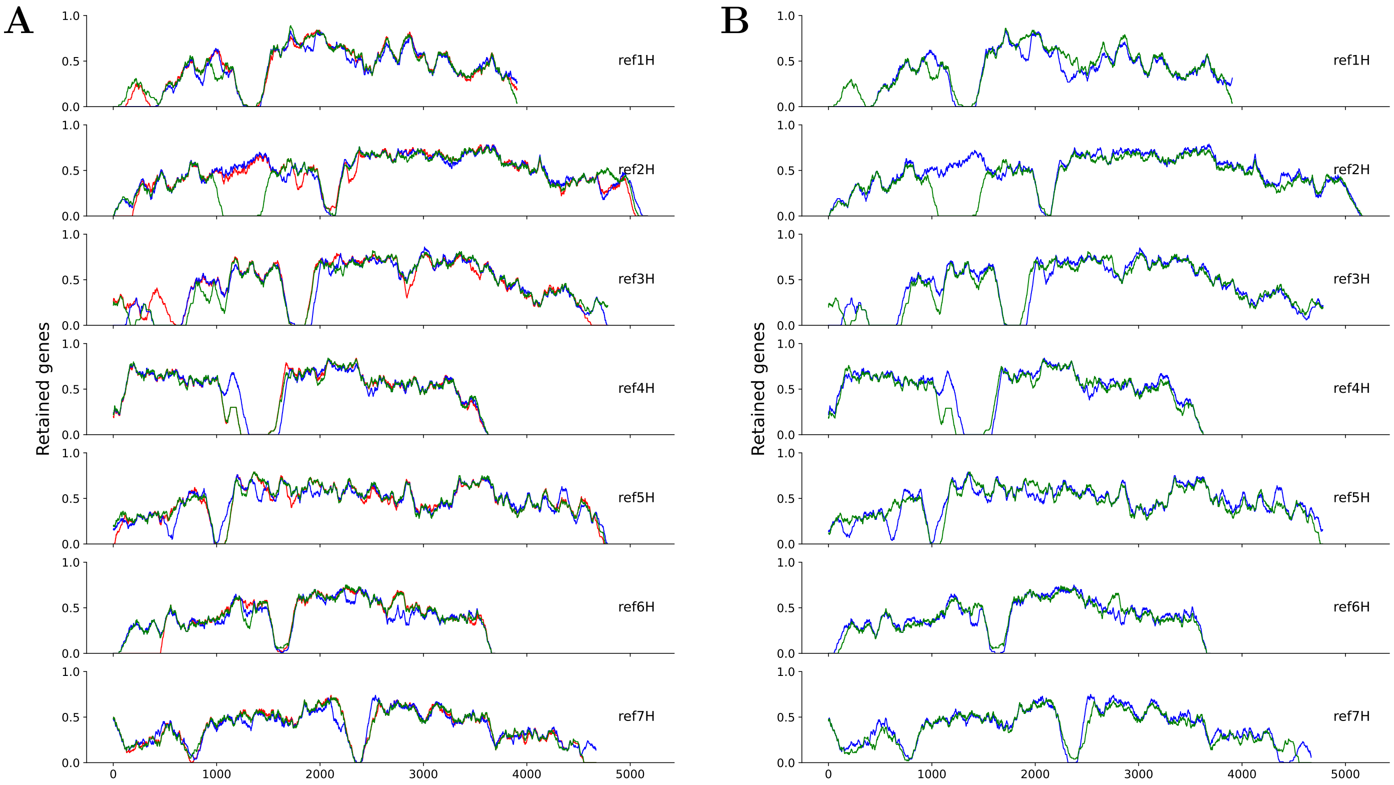

Figure S29. Gene retention of subgenomes of *Avena sativa* (A) and *Avena insularis* (B) with *Hordeum vulgare* as a reference. Red represents subgenome A, blue represents subgenome C, green represents subgenome D.

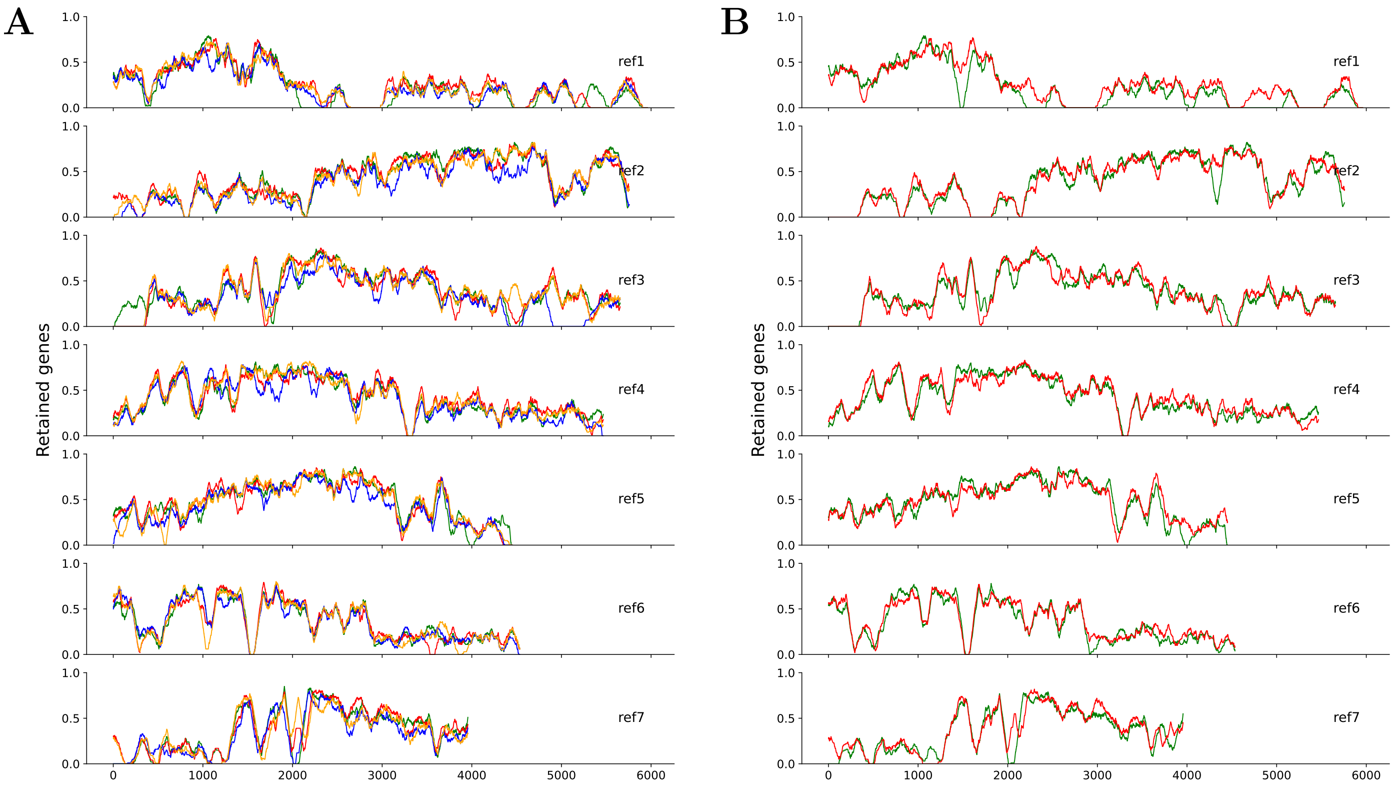

Figure S30. Gene retention of subgenomes of *Papaver setigerum* (A) and *Papaver somniferum* (B) with *Papaver rhoeas* as a reference. Red represents subgenome A, blue represents subgenome B, green represents subgenome C, and orange represents subgenome D.

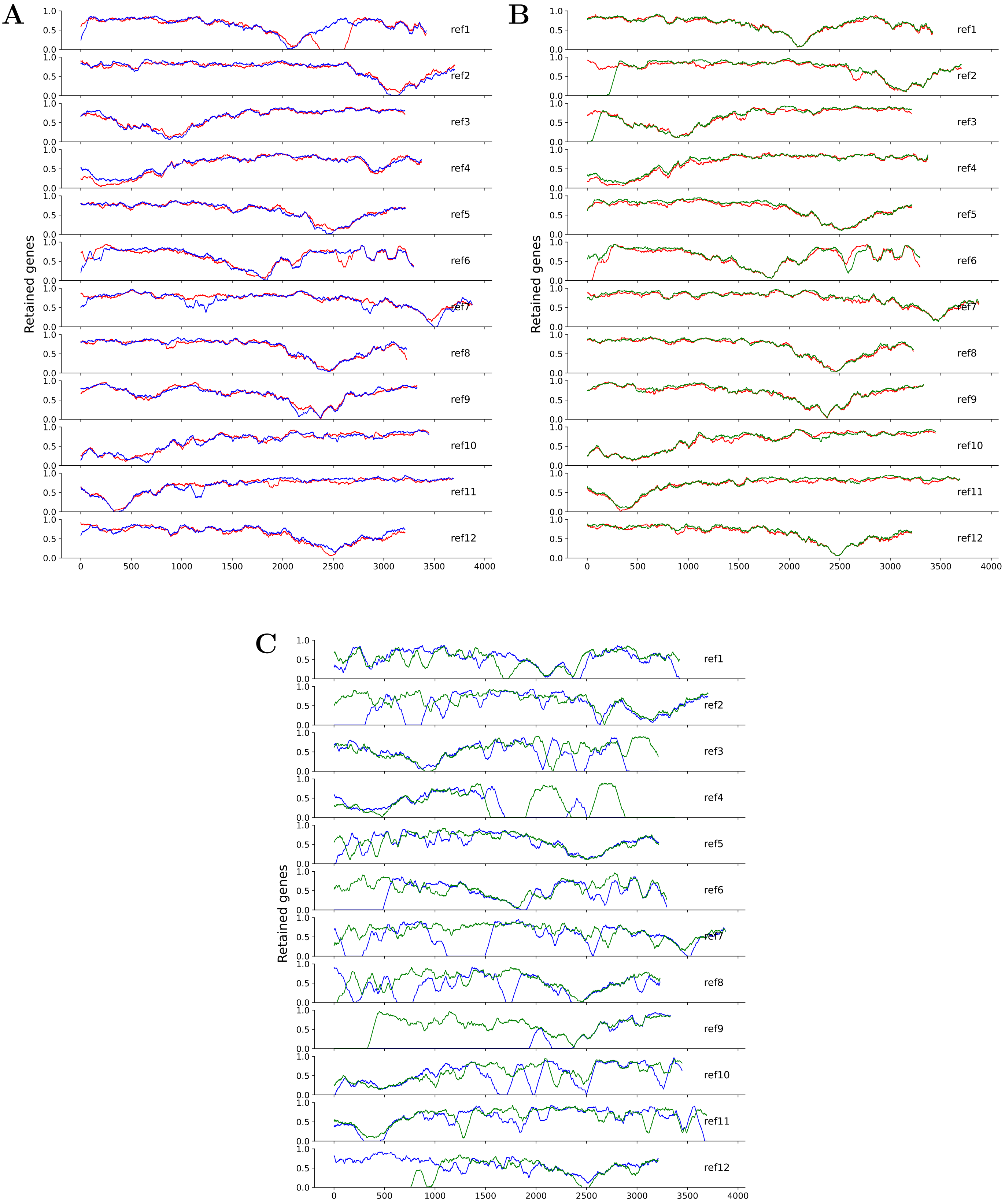

Figure S31. Gene retention of subgenomes of *Brassica juncea* (A), *Brassica napus* (B) and *Brassica carinata* (C) with *Sinapis alba* as a reference. Red represents subgenome A, blue represents subgenome B, and green represents subgenome C.

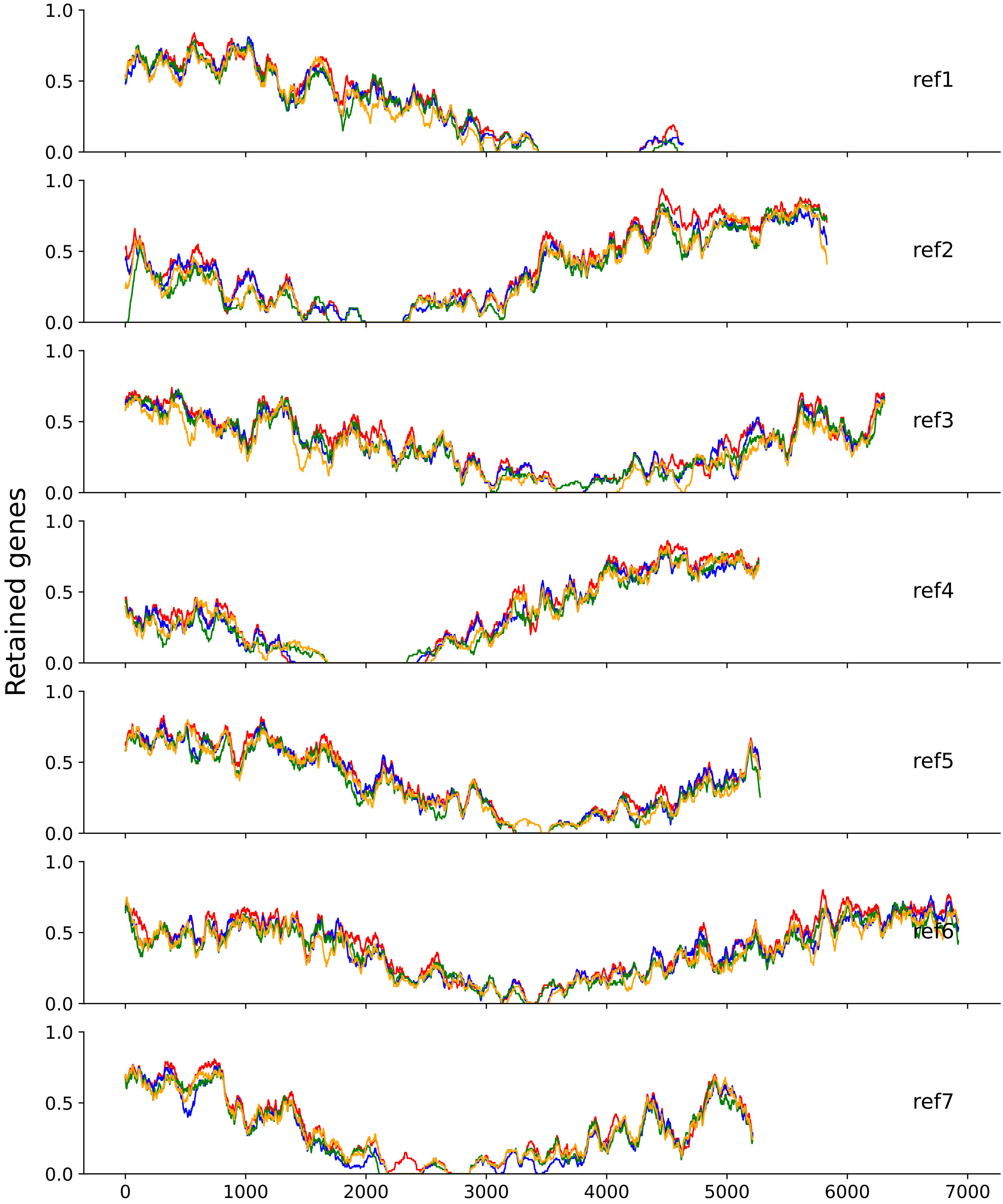

Figure S32. Gene retention of subgenomes of allooctoploid cultivated strawberry (*Fragaria* × *ananassa*) with *Rubus idaeus* as a reference. Red represents subgenome 1 (V), blue represents subgenome 2 (I), green represents subgenome 3 (T2), orange represents subgenome 4 (T1).

Table S1. Genome sources used in this study.

Species	Karyotype	Genotype	Source (URL)
Triticum aestivum	2n=6x=42	AABBDD	https://urgi.versailles.inra.fr/download/iwgsc/
Triticum turgidum	2n=4x=28	AABB	http://ftp.ncbi.nlm.nih.gov/genomes/all/GCA/900/231/445/ GCA 900231445.1 Svevo.v1
Triticum urartu	2n=14	AA	http://ftp.ncbi.nlm.nih.gov/genomes/all/GCF/003/073/215/ GCF 003073215.2 Tu2.1
Aegilops speltoides	2n=14	BB	http://download.big.ac.cn/gwh/Plants/Aegilops_speltoides_ Aegilops speltoides GWHBFXR00000000.1/
Aegilops tauschii	2n=14	DD	http://ftp.ncbi.nlm.nih.gov/genomes/all/GCF/001/957/025/ GCF 001957025.1 Aet MR 1.0
Hordeum vulgare	2n=14	HH	http://ftp.ncbi.nlm.nih.gov/genomes/all/GCF/904/849/725/ GCF 904849725.1 MorexV3 pseudomolecules assembly
Avena sativa	2n=6x=42	AACCDD	https://edal.ipk-gatersleben.de/
Avena insularis	2n=4x=28	CCDD	https://edal.ipk-gatersleben.de/
Avena longiglumis	2n=14	AA	https://edal.ipk-gatersleben.de/
Avena atlantica	2n=14	AA	https://genomevolution.org/coge/GenomeInfo.pl?gid=5333
Avena eriantha	2n=14	CC	https://genomevolution.org/coge/GenomeInfo.pl?gid=5338
Papaver somniferum	2n=4x=14	AACC	https://github.com/xjtuomics/Papaver-Genomics/
Papaver setigerum	2n=8x=44	AABBCCDD	https://github.com/xjtuomics/Papaver-Genomics/
Papaver rhoeas	2n=14	-	https://github.com/xjtuomics/Papaver-Genomics/
Brassica juncea	2n=4x=36	AABB	http://www.oilseedhunan.net/download.html
Brassica napus	2n=4x=38	AACC	http://cbi.hzau.edu.cn/bnapus/
Brassica carinata	2n=4x=34	BBCC	http://ftp.ncbi.nlm.nih.gov/genomes/all/GCA/016/771/965/ GCA 016771965.1 ASM1677196v1
Brassica rapa	2n=20	AA	http://brassicadb.cn/#/Download/
Brassica nigra	2n=16	BB	https://doi.org/10.6084/m9.figshare.21442935.v1
Brassica oleracea	2n=18	CC	https://www.genoscope.cns.fr/externe/plants/
Sinapis alba	2n=24	-	https://doi.org/10.6084/m9.figshare.21442935.v1
Fragaria x ananassa	2n=8x=56	$VVIIT_1T_1T_2T_2$	https://www.rosaceae.org/Analysis/13738092
Fragaria vesca	2n=14	VV	http://eplantftp.njau.edu.cn/Fragaria/F._vesca/F._vesca_v6.0/
Fragaria iinumae	2n=14	II	https://www.rosaceae.org/species/fragaria_iinumae/genome v1.0
Fragaria viridis	2n=14	-	https://www.rosaceae.org/Analysis/9155217
Fragaria nipponica	2n=14	-	https://www.rosaceae.org/Analysis/166
Rubus idaeus	2n=14	-	https://www.rosaceae.org/Analysis/16630509
